# A Triple‐Nanoparticle System for Controlled Graphene Nanosheet Stacking: Enabling K/Na‐Ion Battery Anodes with Ultra‐Fast Charging Exceeding Petroleum Vehicle Refueling

**DOI:** 10.1002/advs.202524370

**Published:** 2026-05-10

**Authors:** Shukai Ding, Le Zhang, Hang Li, Yang Zhang, Guoquan Suo, Bin Han, Dongfeng Sun, Wenqi Zhao, Gaohui Du, Qingmei Su, Bingshe Xu, Zhen Xu, Wei Wang

**Affiliations:** ^1^ School of Physics & Information Science Shaanxi University of Science and Technology Xi'an China; ^2^ School of Materials Science & Engineering Shaanxi University of Science and Technology Xi'an China; ^3^ School of Chemistry and Chemical Engineering Northwestern Polytechnical University Xi'an China; ^4^ Key Laboratory of Interface Science and Engineering in Advanced Materials Ministry of Education Taiyuan University of Technology Taiyuan Shanxi China; ^5^ Shanxi‐Zheda Institute of Advanced Materials and Chemical Engineering Taiyuan Shanxi China; ^6^ School of Metallurgical and Ecological Engineering University of Science and Technology Beijing Beijing China

**Keywords:** carbon anodes, graphene nanosheets, K‐ion batteries, Na‐ion batteries, ultra‐fast charging

## Abstract

Large‐ion (K, Na) battery systems mitigate uneven global lithium distribution, while their ability to attain recharge time shorter than refueling would remove the final barrier for secondary batteries to replace petroleum vehicles. However, their large‐ion chemistry makes ultra‐fast charging an even significant challenge. Controlling and designing the stacking of chemically modified graphene nanosheets (GNS) to tailor multi‐dimensional structures offers great potential in this aspect, which is attributed to the large interlayer distance and topological geometry structure for shortening the ion and electron transfer path and strengthening the absorption of ions. Conventional synthesis methods are confined to pristine 2D sublattices, lacking uniform molecular structures and clear self‐assembly mechanisms. Herein, a triple‐nanoparticles (Tri‐NPs) system is proposed to obtain multi‐dimensional, well‐defined, and accurately stacked GNS structures, including 3D GNS‐sieves, 2D GNS‐holey nanosheets, and 1D GNS‐hollow spheres. Consequently, the 1D GNS‐hollow spheres demonstrate a recharging time comparable to refueling petroleum‐powered vehicles—merely 3.76 min over 1250 cycles at 15.96 C in potassium‐ion batteries (PIBs) and 3.36 min over 3000 cycles at 17.86 C in sodium‐ion batteries (SIBs). This opens new perspectives for addressing the long‐standing criticism of electric vehicles over prolonged charging times through the development of battery systems featuring high‐rate charge–discharge performance and low‐cost materials.

## Graphical abstract

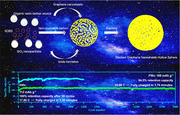

## 1 Introduction

Large‐ion (K, Na) battery systems with ultra‐fast recharge time (shorter than refueling) are elegant and will remove the final barrier to electric vehicles replacing petroleum vehicles. Multi‐dimensional superlattices composed of ordered nanoparticles [1, 2, 3] offer an effective strategy for tailoring multi‐dimensional structures and synergistic functions in inorganic or organic materials [4, 5, 6]. Thus, stacking graphene sublattices into superlattices to fabricate fast‐charging carbon anodes represents the first step toward this goal [7]. However, conventional synthesis methods are limited to pristine 2D sublattices prepared via CVD or mechanical exfoliation, posing a significant practical challenge when stacking chemically modified graphene sublattices—such as polar groups‐modified graphene nanosheets (GNS) [8]. Multi‐type nanoparticle systems, including GNS nanoscale precursors, open an avenue for controlled stacking via self‐assembly [9]. For instance, Zhao et al. explored an approach by mixing “soft micelles” (triblock copolymer Pluronic F127/polydopamine composites) with “hard silica spheres” to tune the microstructure of carbon superlattices. Nevertheless, Pluronic F127—a conventional polymeric surfactant—only converts to non‐crystalline carbon, limiting the practical value of this strategy [10].

To address this issue, polymeric nanoparticles that can transform into GNS are imperative. Over the past two decades, carbon fibers have outperformed graphene in both scientific research and industrial applications. This superiority stems primarily from the molecularly precise transformation and processability of polyacrylonitrile (PAN)‐based carbon fiber precursors [11]. In contrast, research on graphene‐based materials remains confined to CVD and mechanical exfoliation methods, which essentially focus on fabrication processes rather than molecular structure design of starting materials [12]. This approach may be inherently misaligned: it is constrained not only by complex protocols but also by a disconnect between high‐cost infrastructure and practical utility, alongside limited translational potential. Drawing on the success of carbon fibers, redefining and rethinking the physical states and molecular structures of starting materials holds greater scientific and technological promise for graphene‐based materials.

Our group developed an Organic Nano Carbon Source (ONCS) that enables metal‐free, low‐temperature (800–1300°C) synthesis of high‐conductivity GNS‐based materials [13, 14, 15, 16, 17, 18]. ONCS endows the nanocolloidal gel state with processability and scalability advantages by integrating a nano‐polymeric solid phase with a nano‐oily liquid phase. Herein, we envisage a triple‐nanoparticle (Tri‐NPs) system comprising ONCS, SDBS micelles, and SiO_2_ nanoparticles, which exhibits controlled stacking of GNS across multiple dimensions (1D, 2D, 3D) and synergistic functions. Using this strategy, elaborately stacked GNS not only exhibit a high surface‐to‐volume ratio and abundant edge/chemical defects in their porous structure, but also a loose crystal structure [13, 14, 15, 19]. The 1D GNS‐hollow spheres prepared by the Tri‐NPs system (wall thickness: 10.05 nm, inner diameter: 32.5 nm) show the ultrafast surface‐dominated Na^+^ and K^+^ storage at high cycling stability. As a result, the anode delivers a record‐high electrochemical performance of 188 mAh g^−^^1^ at 3 A g^−^^1^ over 1250 cycles—i.e., merely 3.76 min recharge time in PIBs and 112 mAh g^−^^1^ after 2000 cycles at 2 A g^−^^1^ —i.e., merely 3.36 min recharge time in SIBs. The Tri‐NPs system provides new insights into the synthesis of GNS‐based materials for energy storage grids and chip‐level devices in the future.

## 2 Controllability of the Tri‐NPs System for GNS Stacking

**FIGURE 1:**
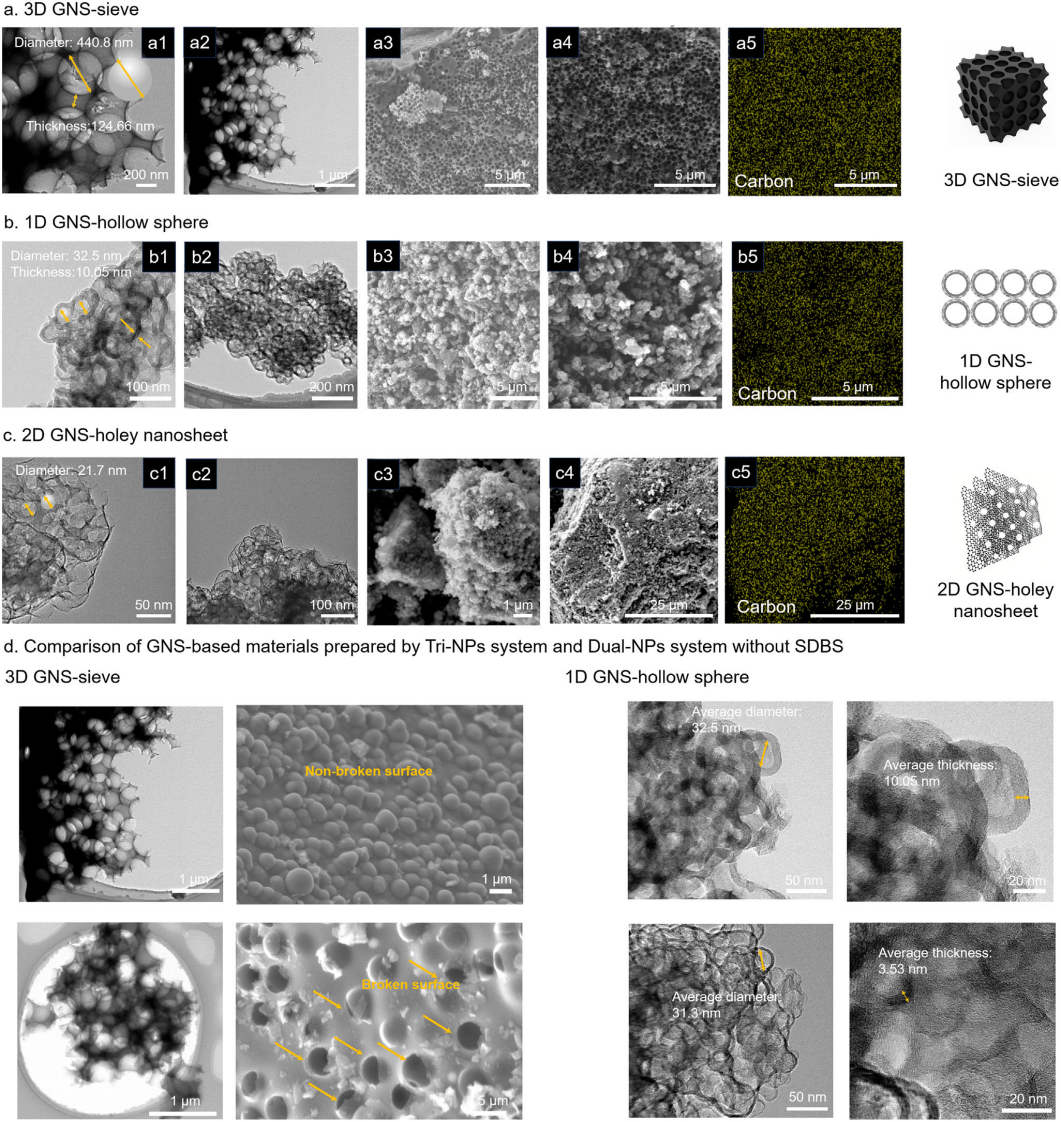
GNS‐based materials prepared by the Tri‐NPs system (a) 3D GNS‐sieves, (b) 1D GNS‐hollow spheres, (c) 2D GNS‐holey nanosheets, and (d) comparison of GNS‐based materials prepared by the Tri‐NPs system and dual‐NPs system without SDBS. In detail, the top‐left panel: 3D GNS‐sieves prepared by Tri‐NPs system; bottom‐left panel: 3D GNS‐sieves prepared by Dual‐NPs system without SDBS; the top‐right panel: 1D GNS‐hollow spheres prepared by Tri‐NPs system; the bottom‐right panel: 1D GNS‐hollow spheres prepared by Dual‐NPs system without SDBS.

The Tri‐NPs system is designed for controlled GNS stacking. Upon calcination and HF etching, ONCS transform into GNS, which facilitates electron transfer. SDBS micelles convert into non‐crystalline carbon, which tunes chemical defects and structural stability [14]. SiO_2_ nanoparticles (as hard templates, in sizes of 500, 100, and 20 nm) are removed, leaving pores for ion transfer. Figure 1 illustrates the three unique GNS‐based materials obtained by the Tri‐NPs system:

Figure 1 *a* *shows the 3D GNS‐sieves (synthesized using 500 nm SiO_2_ nanoparticles in the Tri‐NPs system)*: Within the bulk material (≈100 µm in size), pores with an average size of 440.8 nm (standard deviation: 49.5 nm) are orderly arranged (Figure S1a–c). Intact spherical protrusions on the surface (Figure S1d) indicate the formation of a stable GNS layer with a thickness of 124.66 nm (Figure S1e–g). Compared to graphite‐derived 3D porous graphene architectures, the ONCS‐derived 3D GNS‐sieves exhibit more homogeneous pores and a non‐wrinkled sheet structure, which is attributed to the controllable self‐assembly of ONCS [20]. The 3D GNS‐sieves hold significant potential in ion‐selective applications and water treatment fields [21].

Figure 1*b* *presents the 1D GNS‐hollow spheres (synthesized using 100 nm SiO_2_ nanoparticles in the Tri‐NPs system)*: The flower cluster in Figure S2a–e is formed by the aggregation of 1D GNS‐hollow spheres with an average diameter of 32.5 nm (Figure S2f) and a shell thickness of 10.05 nm (Figure S2g). Over the past two decades, carbon hollow spheres (CHS) have emerged as an important branch of carbon materials, owing to their balanced surface‐to‐volume ratio and high‐volume density [22, 23].

Figure 1*c* *depicts the 2D GNS‐holey nanosheets (synthesized using 20 nm SiO_2_ nanoparticles in the Tri‐NPs system)*: The flower cluster in Figure S3a–d is formed by the aggregation of 2D GNS‐holey nanosheets with widths of 180.07 and 82.49 nm (Figure S3e). The uniform holes have an average size of 24.38 nm (Figure 1c). Generally, chemical and physical etching are employed for preparing holey graphene, which exhibits uncontrollable complexity [24, 25]. To our knowledge, the bottom‐up strategy via a self‐assembling nanoparticle system has primarily been used for preparing holey graphene nanosheets to date, except for the synthesis of small organic molecules [26, 27, 28].

All the GNS materials exhibit morphological homogeneity and uniformity (Figure 1a2,3‐c2,3), which indicates that the SDBS micelle and ONCS homogeneously involve the self‐assembly process without phase separation. Elemental analysis confirms the presence of pure carbon with no silicon residues (Figure 1a4,5‐c4,5). These results validate the Tri‐NPs system as a robust strategy for constructing GNS architectures. By comparison, conventional graphite‐derived strategies (e.g., for preparing holey graphene and graphene fibers) yield GNS materials with uncontrollable structures, owing to the heterogeneous microstructure of pristine graphite.

## 3 Self‐Assembly Mechanism of Tri‐NPs System for GNS Stacking

To further clarify the interactions within the Tri‐NPs system, a dual‐nanoparticles (Dual‐NPs) system—comprising ONCS and SiO_2_ nanoparticles—is employed. After calcination, this system yielded a 3D GNS‐sieves, 1D GNS‐hollow spheres, and 1D GNS‐nanotubes (Figure S4–S8). The nearly identical structures indicate that the GNS structural skeleton is transformed from ONCS to perfectly replicate the self‐assembled morphology of SiO_2_ nanoparticles. It is attributed to a strong hydrophobic‐hydrophobic interaction between ONCS (attributing to the hydrophobic oil phase of Labrafac WL 1349 (HLB = 1)) and SiO_2_ nanoparticles (attributing to the modification by octadecyltrimethoxysilane (ODS)) [29, 30]. The strong interaction between ONCS and SiO_2_ nanoparticles is further evidenced by the integrated encapsulation, as shown by comparing the morphologies before and after HF etching (Figure S9).

The comparison between the GNS‐based material prepared by Tri‐NPs and the Dual‐NPs system exhibits that the Dual‐NPs system gives a thinner and more fragile carbon layer in Figure 1d. The 3D GNS‐sieves prepared by the Dual‐NPs system display holes on the surface in Figure 1d left (Figures S5 and S6). Similarly, the 1D GNS‐hollow spheres (Figure 1d right (Figure S7a–f in detail) have a thinner GNS layer at ∼3.53 nm, which is only one‐third of that (10.05 nm) observed in the 1D GNS‐hollow spheres from the Tri‐NPs system. Furthermore, carbon materials calcinated by a Dual‐NPs system, consisting of SDBS micelles and SiO_2_ nanoparticles, show irregular and heterogeneous aggregation (Figures S10a–c and S11a–c), and the carbon layer has an uncertain open or closed state at the nanoscopic level (Figures S10d–f and S11d–f). It indicates that the weak interaction of SiO_2_ nanoparticles and SDBS micelles results in the separated phases during calcination, which stems from the more hydrophilic surface of SDBS micelles due to the sulfonate group. Moreover, A single‐nanoparticle system of SDBS micelles results in a disorganized carbon material structure, including sheets or bulks in Figure S12, which indicates that the single SDBS micelles hasn't ability for a stable structure. These results demonstrate that the role of SDBS micelles is to fill the gaps between SiO_2_ nanoparticles and ONCS for a thicker carbon layer.

Furthermore, the Dual‐NPs system of SDBS micelles and ONCS (Figure S13) produces GNS‐based materials with a homogeneous sheet structure or an ultra‐thin layer at both low and high concentrations of SDBS. It further supports the structural skeleton effect of ONCS for the homogeneous self‐assembly process. For types of carbon materials, pure ONCS yields a clear GNS‐based sheet state in a previous report [13]. The blurred state of the carbon materials prepared by SDBS micelles indicates a non‐crystalline carbon state.

## 4 Gradient Molecular Pyrolysis Mechanism for ONCS‐to‐GNS Transformation

**FIGURE 2:**
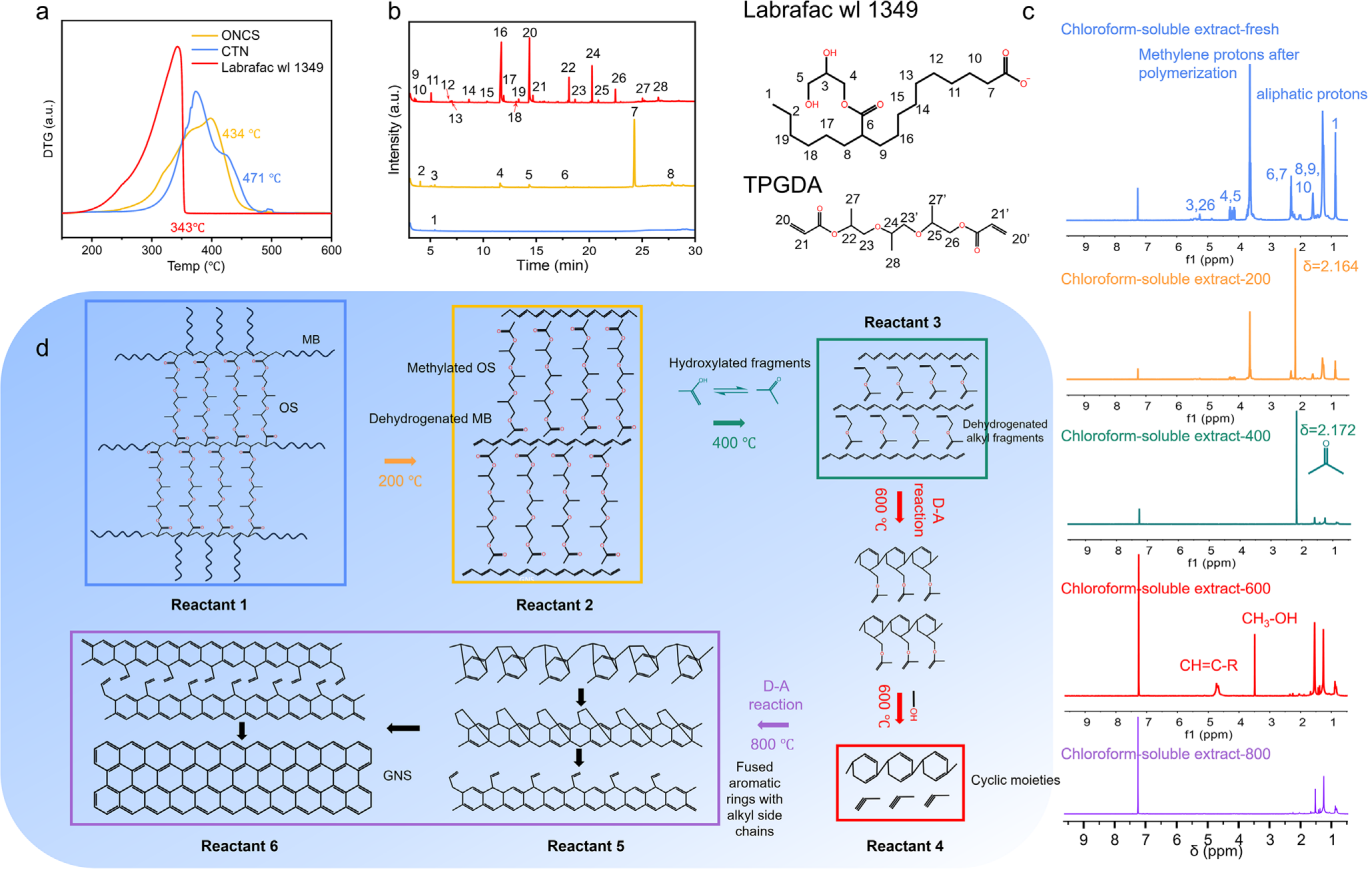
(a) Derivative thermogravimetry and (b) GC‐MS spectrum of calcinated ONCS, calcinated Labrafac WL 1349, and calcinated CTN, (c) NMR characterization of the chloroform‐soluble extract at different calcination temperatures (the label "extract‐number" denotes the chloroform‐soluble extract obtained after calcination at the corresponding temperature (e.g., extract‐600 represents the sample calcined at 600°C)), and (d) the gradient molecular pyrolysis transformation of ONCS to GNS.

Interpretation of the ONCS degradation mechanism is of great significance, as it paves the way for the bottom‐up synthesis of GNS through polymeric molecular design. ONCS represent a nanoscopic co‐existence state of liquid and solid organic precursors. It is composed of solid crosslinked tripropylene glycol diacrylate (CTN) and liquid medium‐chain triglycerides (Caprylic/capric triglyceride, Labrafac WL 1349), enabling the metal‐free, low‐temperature (800–1300°C) synthesis of GNS [17, 18]. The nanoscopic co‐existence of liquid and solid states is evidenced by the comparison of the irregular liquid membranes and solid molecules observed by TEM (Figure S14a,b) and the solid CTN phase self‐assembles into hollow spheres (Figure S14c,d) after the removal of the liquid phase via ethanol dialysis. The phenomenon indicates that the CTN is composed of molecular fragments within the Labrafac WL 1349 nanodroplets, which endow it with the controllable self‐assembly capacity [15, 16]. Derivative thermogravimetric analysis (DTG) of ONCS reveals that its end temperature of degradation increases to 434°C—up from 343°C (the degradation temperature of Labrafac WL 1349)—and is lower than that of CTN (471°C) (Figure 2a). The single peak in the DTG curve indicates that the liquid and solid phases in ONCS form a single phase.

Furthermore, the degradation of Labrafac WL 1349 and CTN yields only alkyl compounds (Figure 2b, Figure S15), whereas ONCS exhibits strong aromatization (compound 7 in Figure 2b) in gas chromatography‐mass spectrometry (GC‐MS). These degradation differences highlight that the nanoscale liquid–solid phase coexistence is responsible for GNS formation. To gain insight into the degradation process, NMR and FTIR are employed to analyze chloroform‐soluble extracts and calcinates prepared under different calcination temperatures, respectively (Figure 2c, Figure S16). Comparison with 1H NMR spectra of TPGDA, Labrafac WL 1349, and fresh ONCS (Figure 2c, Figures S17 and S18) confirms excellent mixing of the solid and liquid phases. The disappearance of the terminal alkenyl signals in TPGDA (peaks 20 and 21 in Figure S18) and the increased broad methylene proton signals (δ = 3–4) linking to the carbonyl group in fresh ONCS extracts indicate complete polymerization and formation of a crosslinked molecular network (Figure 2c). A network polymer with a methylene backbone (MB) and oxygen‐rich side chains of R_1_─C═O─O─R_3_─O─R_4_─O─R_5_─O─C═O─R_2_ (OS) is shown in Figure 2d (**Reactant 1**).

During the calcination process, increasing the temperature to 200°C causes degradation of the ─MB─ R_1_─C═O─O─•••─O─C═O─R_2_─MB─ structure, evidenced by intensified carbonyl signals (1H NMR, δ = 2.16) and double bonds (Figure 2d, **Reactant 1→2**; i.e., dehydrogenated MB and methylated R_1_−OS−R_2_). Concurrently, Labrafac WL 1349 undergoes the same pyrolysis (Figure S19), resulting in degradation of polar carbonyl and hydroxyl groups, increased hydrophobicity of the liquid phase, and transformation of ONCS from a gel to semi‐solid state. The degradation reaction of the MB‐R_1_─C═O─O─R_2_─OS structure is supported by a significant decline in proton content at δ = 1.0–4.0 (Table S1).

At 400°C, the MB is fully carbonized and becomes insoluble, as confirmed by the disappearance of the MB proton signal, while cleavage of ether and ester bonds in the methylated R_1_−OS−R_2_ produces downfield‐shifted carbonyl signals (δ = 2.17) (Figure 2d, **Reactant 2→3**; i.e., hydroxylated fragments and dehydrogenated alkyl fragments). Calcination at 600°C further converts dehydrogenated MB and dehydrogenated alkyl fragments to cyclic moieties (**Reactant 3→4**) due to a plentiful of double bonds, presumably via Diels–Alder cycloaddition. At 800°C, no small polar molecules are detectable by NMR. Aromatization yields a polycyclic aromatic hydrocarbon (PAH) backbone consisting of fused aromatic rings with alkyl side chains (**Reactant 4→5**). Cycloaddition of PAHs results in the GNS structure in Figure 2d.

Consequently, gradient molecular pyrolysis of the ─MB─OS─MB─ network structure is demonstrated by the low‐temperature degradation of ─MB─ R_1_─C═O─O─•••─O─C═O─R_2_─MB─ to form dehydrogenated MB, and high‐temperature degradation of the methylated R_1_−OS−R_2_ to form dehydrogenated alkyl fragments, respectively. This is further supported by IR spectral evolution (Figure S16), which shows the release of carbonyl and hydroxyl compounds. Specific small molecules are only detected at specific calcination temperatures, indicating that ONCS pyrolysis is strictly thermodynamically constrained. Following this pyrolysis pathway, PAH and GNS form through reactions between dehydrogenated MB and dehydrogenated alkyl fragments—indicating that these dehydrogenated alkyl fragments are trapped within dehydrogenated MB for the final cycloaddition step

Two reasons are proposed for the observed gradient molecular pyrolysis transformation of ONCS to GNS: either the polymeric network forming a sheet structure in liquid nanodroplets (potentially an anisotropic mechanism) or physical environment confinement in nanodroplets (Figure 2d). no anisotropic properties of ONCS are observed via a micro‐polariscope (Figure S20). The result proves that the molecular fragments of CTN in nanodroplets is dissociated state, not a sheet structure. There isn't a molecular interaction between the molecular fragments of CTN in nanodroplets to limit the pyrolysis and release of small molecules. On the other side, the calculated Laplace pressure in liquid nanodroplets increases rapidly with decreasing sphere radius (Figure S21), especially for radii<100 nm. Thus, it is suspected that the liquid molecule supports a super‐high‐pressure environment for the CTN pyrolysis due to nano‐physical confinement. It not only elevates degradation temperature, traps alkyl fragments within MB, but also amplifies differences in the thermodynamic stability of chemical bonds—thereby inducing gradient molecular pyrolysis. Consequently, the −MB−OS−MB− network structure and a super high‐pressure pyrolysis environment are critical for introducing double bonds into MB and side chains. This proposed mechanism is expected to enable efficient, cost‐effective, and controllable GNS synthesis.

**FIGURE 3:**
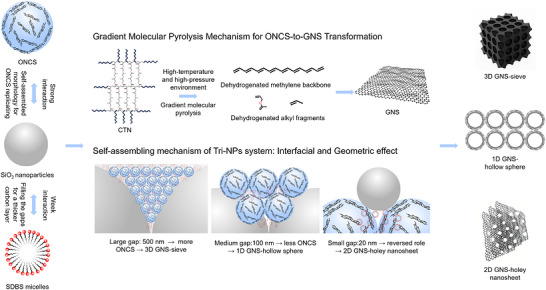
Schematic illustration from the Tri‐NPs system to GNS‐based materials driven by the gradient molecular pyrolysis and the self‐assembly mechanism.

Overall, the whole process is interpreted in Figure 3. The gradient molecular pyrolysis mechanism of ONCS supports a basic block of GNS. Interfacial interactions of the Tri‐NPs system account for both morphological replication and carbon layer thickness. Furthermore, the geometric effect plays a critical role in the GNS structural skeleton during multi‐dimensional self‐assembly. Specifically, the large (500 nm) and medium (100 nm) gaps between SiO_2_ nanoparticles are filled by ONCSs (30 nm) and SDBS micelles (4–6 nm). Larger gaps can accommodate more ONCS and SDBS micelles. Consequently, as the number of ONCS decreases, the continuous structure of the 3D GNS‐sieves transforms into 1D GNS hollow spheres. For the 2D GNS‐holey nanosheets, SiO_2_ nanoparticles reverse their role to fill the gaps between ONCS, owing to the reversed geometric relationship.

## 5 GNS‐Based Materials for High‐Performance Large‐Ion Batteries

Sodium‐ion batteries (SIBs) and potassium‐ion batteries (PIBs) have attracted significant attention as sustainable alternatives to lithium‐ion batteries (LIBs), primarily owing to the abundant raw materials [31, 32, 33, 34]. However, the large ionic radii of Na^+^ and K^+^ induce slow ion dynamic transfer, inhomogeneous metal deposition, and severe structural stress, leading to a time‐consuming charging process and unstable cycling. Carbon‐based anodes with precisely tailored nanostructures and doping exhibit long cycling stability at current densities below 10 C (e.g., carbon onions or heteroatom‐doped carbons) [35, 36]. For example, Qiu et al. demonstrate the long cycling at 7.75 C in PIBs [37]. Hu demonstrated the hard carbon anode at 6.5 C in SIBs, which is full‐charged in 9 min [38]. He et al. pointed out that maintaining stable high‐capacity operation at elevated charge/discharge rates for SIBs is vital for practical implementations coping with the current trajectory of global electrification [39, 40, 41, 42, 43, 44, 45, 46, 47].

**FIGURE 4:**
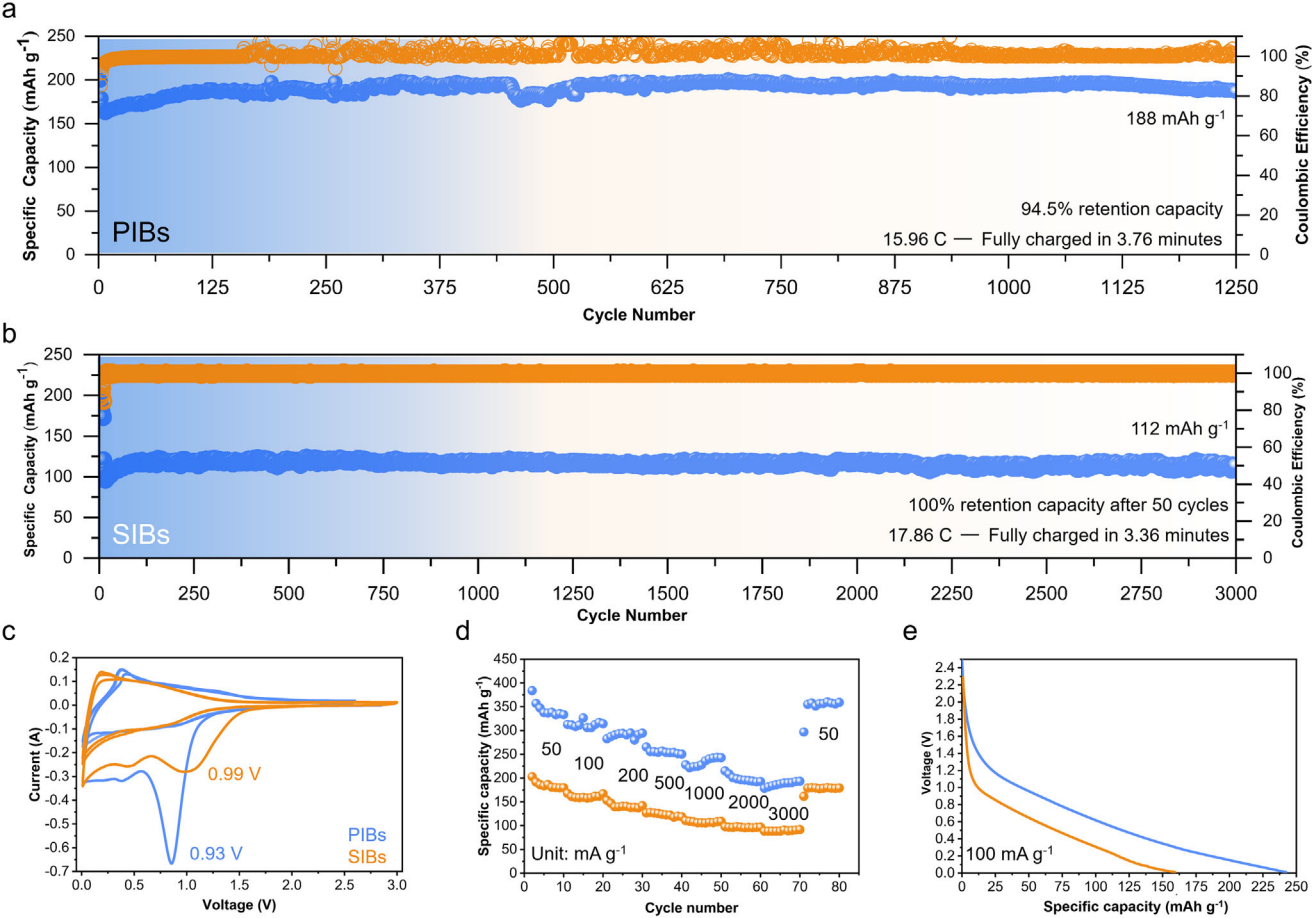
(a,b) Long cycling performance of 1D GNS‐hollow spheres at current density of 3000 mA g^−1^ in PIBs and 2000 mA g^−1^ in SIBs, and (c–e) CV curve, rate performance, and capacity‐voltage curve of 1D GNS‐hollow spheres in PIBs and SIBs.

Herein, various GNS‐based nanomaterials are assembled into PIB half‐cells and tested at a current density of 50 mA g^−^^1^ (Figure S22a). As a control, the anode derived from the calcination of pure SDBS micelles exhibits the lowest specific capacity and fastest fading. The 2D GNS‐holey nanosheets deliver a specific capacity of up to 274 mAh g^−^^1^, approaching the theoretical capacity of the intercalation compound KC_8_. This indicates superior electrochemical performance at low current densities compared to other holey graphene anodes prepared via graphite‐or CVD‐driven strategies [48, 49, 50, 51]. In contrast, the 3D GNS‐sieves show rapid capacity fading (Figure S22b). Cycling tests at 500 mA g^−^^1^ for Tri‐NPs‐derived GNS nanomaterials revealed stable capacities of 137–170 mAh g^−^^1^ (Figure S23a), with the 1D GNS‐hollow spheres exhibiting the highest specific capacity. At a higher current density of 1 A g^−^^1^ (Figure S23b), the 2D GNS‐holey nanosheets and 3D GNS‐sieves fade completely after 500 and 800 cycles, respectively. By comparison, the 1D GNS‐hollow spheres retain a specific capacity of 140 mAh g^−^^1^ after 1000 cycles at 1 A g^−^^1^. Notably, at a high current density of 3000 mA g^−^^1^, it maintains a specific capacity of 188 mAh g^−^^1^ over 1250 cycles with 94.5% retention capacity in PIBs (Figure 4a). Similarly, the 1D GNS‐hollow spheres shows long cycling stability over 3000 cycles at 2000 mA g^−^^1^ (112 mAh g^−^^1^; Figure 4b). After a slow fade in initial 50 cycles, the 100% retention capacity keeps up to end. Such high current density gives a full charge in 3.76 and 3.36 min for PIBs and SIBs, respectively, which have overwhelmed the petroleum‐powered vehicles at refuel time. The advantage of the 1D GNS‐hollow spheres in charge time has been demonstrated in Table S2. CV measurements identify the broad reversible charge–discharge voltage range in PIBs and SIBs, which indicates an ion adsorption‐dominated storage mechanism (Figure 4c).

During the first discharge process, the irreversible reduction peaks located at approximately 0.93 V (PIBs) and 0.99 V (SIBs) are attributed to the formation of the solid electrolyte interphase (SEI) [42]. Subsequently, a broad reduction peak repeatedly appears in the potential range of 0–0.5 V for stable cycling. For rate performance in Figure 4d, it retains nearly 100% of its initial capacity up to 3000 mA g^−^^1^ in PIBs and SIBs. The smooth slope of the charge–discharge curves (Figure 4e) further supported the ion adsorption mechanism for the 1D GNS‐hollow spheres. The voltage vs. specific capacity curve in high current density shows high reversibility in Figure S24. Finally, the direct microscopic observation of the 1D GNS‐hollow spheres anode in PIBs (millimeter scale) through quartz windows (Figure S25) and videos in the extended data show stable and reversible surface morphology after charge–discharge at high current densities (200–3000 mA g^−^^1^).

Based on these electrochemical results, the 1D GNS‐hollow spheres (excellent high‐current‐density performance) and 2D GNS‐holey nanosheets (excellent low‐current‐density performance) are selected as the focus for subsequent structural and chemical characterization, as well as mechanistic investigation.

To unravel the origin of their distinct electrochemical performance, the microstructural and chemical properties of the 1D GNS‐hollow spheres and 2D GNS‐holey nanosheets are systematically characterized. Raman spectrum analysis (Figure S26a) shows a high I_D_/I_G_ ratio, indicating abundant defects—where the D band (representing chemical and edge defects) at 1350 cm^−^^1^ and the G band at 1590 cm^−^^1^ [52]. The 1D GNS‐hollow spheres have a lower I_D_/I_G_ ratio (0.93) than the 2D GNS‐holey nanosheets (1.03), suggesting fewer defects. Corresponding XRD patterns (Figure S26b) reveal that the 1D GNS‐hollow spheres had a smaller d‐space (0.3502 nm) and larger La (1.687 nm) compared to the 2D GNS‐holey nanosheets (d‐space = 0.3597 nm, La = 1.05 nm), indicating denser stacking and higher crystallinity. Additionally, the SiO_2_ nanoparticle (a non‐catalytic template) and low calcination temperature (500∼900°C), resulting in high graphitization, highlight the advantage of organic nanocarbon sources (ONCS) for GNS synthesis [22].

Elemental and chemical bond analysis (Figure S26c) shows that the 1D GNS‐hollow spheres have a high carbon–oxygen ratio in the total spectrum, with a distinct C─O─O bond at 288.3 eV (Figure S26d). In contrast, the 2D GNS‐holey nanosheets exhibit a peak at 290.3 eV, indicating more π–π* stackings [53]. BET adsorption–desorption curves (Figure S26e) reveal that the 1D GNS‐hollow spheres have type IV isotherms with a surface area of 59.8 m^2^ g^−1^ and pore volume of 0.31 cm^3^ g^−^^1^ (weak N_2_‐GNS interaction), and their mediocre surface area suggested ultra‐high electrochemical performance is attributed to surface chemistry and stable structure [54]. The 2D GNS‐holey nanosheets had type III isotherms, a higher surface area (145.4 m^2^ g^−^^1^), and pore volume (0.68 cm^3^ g^−^^1^), with a micro‐meso porous hybrid structure (Figure S26f), while the 1D GNS‐hollow spheres show a narrower mesoporous distribution [55].

Further analysis of the electrochemical mechanism is conducted to clarify the structure‐performance relationship of the two high‐performance samples. The capacitive‐controlled and diffusion‐controlled processes are analyzed (Figure S26g–l): CV curves at different scan rates (0.2∼1 mV s^−^^1^) show similar shapes, indicating consistent potassium storage processes (Figure S26g and j). A high ratio of capacitive‐controlled to diffusion‐controlled processes confirms dominant potassium ion adsorption/desorption on the GNS surface, with capacitive contribution increasing with scan rate. The 1D GNS‐hollow spheres exhibit higher capacitive contribution (especially at low scan rates) and the increase of capacitive contribution faster than that of the 2D GNS‐holey nanosheets (Figure S26h–k). Generally, the potassium‐storage mechanism can be analyzed by the relationship between peak current and scan rate, as Formulas S1 and S2. The b value in Figure S27a,b is determined by the slope of the log(v)‐log(i) plots. In detail, the b value approaching 1.0 manifests a capacitive‐controlled process, whereas approaching 0.5 represents a diffusion‐controlled intercalation process [56]. The b value of cathodic and anodic peaks in the 1D GNS‐hollow spheres is 0.867 and 0.826, respectively, which indicates potassium ion storage is dominated by the capacitive‐controlled behavior. The 2D GNS‐holey nanosheets show the values of 0.96 and 0.93, respectively. Furthermore, a significantly larger first semi‐circle in the electrochemical impedance spectroscope (EIS) of 2D GNS‐holey nanosheets at the fresh state shows a high resistance for transfer from the electrolyte to the electrode, which indicates poor wetting in Figure S27c. Different from a 2D‐GNS holey nanosheet, 1D GNS‐hollow spheres at fresh and at 10^th^ give a small semi‐circle, which represents a facilitated electron transfer from the surface of the electrode. It is attributed to the fact that more C─O─O polar bonds and topological geometry structure including appropriate pore size and distribution and more continuous GNS structure promote the electron and ion transfer [56].

**FIGURE 5:**
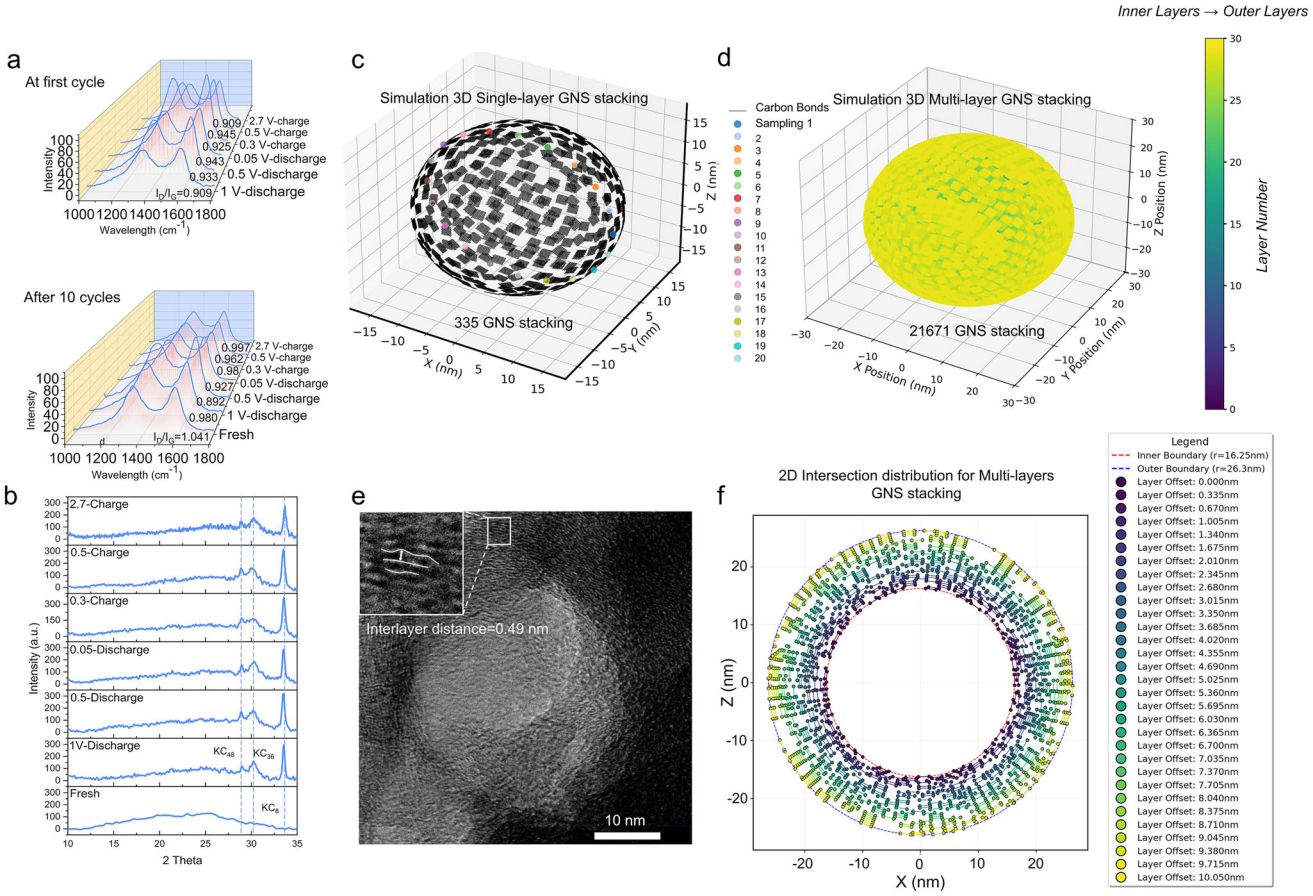
(a) Ex‐Raman spectrum, (b) Ex‐XRD pattern of 1D GNS‐hollow spheres at 3000 mA g^−1^ in PIBs, (c) 3D graph of simulating the single‐layer GNS stacking for the hollow sphere, (d) 3D graph of simulating the multi‐layer GNS stacking for the hollow sphere, (e) HRTEM of cross‐section of 1D GNS‐hollow spheres, and (f) intersection lines distribution for multi‐layer GNS stacking.

To further demonstrate the potassiation/depotassiation mechanism of a 1D GNS‐hollow spheres at 3000 mA g^−1^, the selected six distinct voltage locations (discharging at 1, 0.5, and 0.05 V et charge at 0.3, 0.5, and 2.7 V), corresponding in Figure 4c are tested at the ex situ Raman spectra at the first cycle and the stabilization state after 10 cycles in Figure 5a. It is necessary to consider the inhomogeneity of the electrode surface during the analysis of the Raman measurements. Based on this, the multi‐testing is measured at a given voltage following the overall trend selected. In the first cycle, the I_D_/I_G_ ratio decreases from 1.041 in the fresh state to 0.909 at 1 V discharge, calculated in Table S3, which indicates a solvation effect of 3 M KTFSI. During the first discharge, the increase from 0.909 to 0.933 of the I_D_/I_G_ ratios at 0.5 V discharge comes from the SEI formation. Decreasing the voltage of discharge to 0.05 V, the intercalation of potassium ions increases to the I_D_/I_G_ ratio at 0.943 again. Furthermore, the recovery from 0.943 to 0.909 of the I_D_/I_G_ ratios for the initial state. After 10 cycles in Figure 5a, the cycling performance is stable and the I_D_/I_G_ ratio transforms from 0.980 at 1 V to complete intercalation and adsorption for 0.927 at 0.05 V during the discharging, then recovering to 0.997 at 2.7 V charging, which is explained to the decrease of edge defects by potassium ion storage capacitive‐dominated in Table S4 [40, 57]. To further distinguish capacitive‐dominated processes, ex situ XRD spectra in Figure 5b show the phase of the intercalation compound of KC_8_ (2θ = 33.47), KC_36_(2θ = 30.25), and KC_48_(2θ = 28.89) keep stable after 10 cycles, which indicates the intercalation process doesn't involve the potassium storage at 3000 mA g^−1^ in following cycle [58]. Finally, the electrochemical performance of 1D GNS‐hollow spheres achieves an unprecedented cycling performance at such high current density compared to other typical pure carbon materials in Figure S28. It demonstrates that the ultra‐fast adsorption process can be achieved by the sophisticated design of the stacked GNS structure.

However, while C─O─O defects of surface chemistry and porous structure of the 1D GNS‐hollow spheres have been confirmed for high electrochemical performance by simulations and experiments in other reports (e.g., on onion carbon) [43, 59]. These factors alone are insufficient to explain why the 1D GNS‐hollow spheres outperform reported carbon anodes. Thus, a Python program is developed to simulate GNS stacking and unveil the actual topological geometry structure. During calcination, ONCS transforms into GNSs (1.75×1.8 nm; Figure S29; structural parameters calculated from XRD data in Figure S26) via a random, simultaneous process—unlike catalysis growth process. The simulation randomly and simultaneously deposits process for GNSs onto a 32.5 nm‐diameter spherical surface (structural parameters from TEM in Figure 1b) is achieved using a Fibonacci sphere algorithm, which maintains distances within the Van der Waals radius limit (0.335 nm). This yielded 335 GNSs, confirming that spontaneous, simultaneous ONCS transformation prevents aligned, dense stacking in Figure 5c. The observed 335 GNSs are far below the theoretical value of 1058 (calculated by dividing the spherical surface area by the area of a single GNS: 3,316.6/3.135). In addition, a simulated dense stacking of GNS in a 2D plane with the same area show total of 516 GNS in Figure S30, which is over higher than 335 GNSs randomly stacked in surface of a sphere.

In detail, 20 random 5×5 nm XZ areas on the equatorial plane are analyzed (Figure S31) for single‐layer GNS stacking in Figure 5c: 12 areas contain one GNS, 1 area contains none, and 7 areas contain two GNSs in Figure S31. The shortest distance between two GNSs is calculated as 0.85 nm (Figure S31). Furthermore, the multi‐layer GNS stacking in a hollow sphere with a diameter of 32.5 nm and a thickness of 10.05 nm is simulated for the same state as a 1D GNS‐hollow sphere in Figure 5d. 21671 GNSs are used for stacking the structure. Cross‐section HRTEM of 1D GNS‐hollow spheres in Figure 5e shows the stacking GNS structure, interlayer distance at some locations is larger (0.49 nm) than that of graphite (0.335 nm), although it doesn't reflect the actual stacking state of the GNS. A 2D intersection distribution of 1746 intersecting lines at y = 0 of simulated 1D GNS‐hollow spheres in Figure 5f corresponds to 3492 edges and directly demonstrates a lot of voids for ion transfer. Consequently, the Tri‐NPs System results in loose stacking of GNS in the 1D GNS‐hollow spheres due to a random and simultaneous synthesis process, with abundant intra‐layer voids.

In practice, the active materials loading, the negative/positive materials ratio, and the manufacturing process have a significant influence on the full‐cell. However, the active materials loading is set over 1 mg cm^−2^ to balance the influence of the lab and manufacturing process due to the lack of a calendering process in the lab electrode preparation process. Thus, Figure S32 shows the slight decay of electrochemical performance at 2 mg cm^−2^ compared to the performance in Figure 4. 80 mAh g^−1^ at 1000 cycles in current density of 3000 mA g^−1^ in PIBs and 70 mAh g^−1^ at 1500 cycles in current density of 2000 mA g^−1^ in SIBs have been observed, which is attributed to the lack of a continuous calendering‐dry process. In addition, the potassium ion and sodium ion full cell of 1D GNS‐hollow spheres as anode is built by the 3,4,9,10‐perylenetetracarboxylic dianhydride (PTCDA) and Na_3_V_2_(PO_4_)_3_ as cathode. Figure S33a shows the PTCDA can support a specific capacity of 53 mAh g^−1^ during 2.0∼3.0 V. After assembled to the full cell, a broad discharge and charge voltage range of 0.5∼3.5 are observed, the specific capacity is obtained at around 20 mAh g^−1^ with N/P ratio of 1.05 in Figure S33c. Figure S33b shows the Na_3_V_2_(PO_4_)_3_ can support a specific capacity of 120 mAh g^−1^ at 3.4 V. After assembled to the full cell, a broad discharge and charge voltage range of 2.7–3.4 are observed, the specific capacity is obtained at around 35 mAh g^−1^ with N/P ratio of 1.05 in Figure S33d. The results show 1D GNS‐hollow spheres has potential as anode in practice. But the sophisticated manufacturing process and design are needed to achieve high electrochemical performance. In addition, the results show the SIBs have higher potential in practice compared to the PIBs. Also, translating the ultra‐fast charging performance of 1D GNS‐hollow spheres anodes to practical full‐cell K/Na‐ion batteries remains challenged by cathode matching in ultra‐high‐rate adaptability. Conventional cathodes lack fast ionic/electronic kinetics to tolerate ultra‐fast charging rates matching the anode, leading to severe polarization, irreversible phase transitions, and kinetic mismatch. Future efforts will focus on (e.g., fluorinated polyanions) and engineering cathode surfaces with artificial CEI to enable stable, high‐rate cation de/intercalation for full‐cell ultra‐fast charging.

## 6 Conclusion

We demonstrate a triple‐nanoparticle (Tri‐NPs) system for tailoring the multi‐dimensional architectures and synergistic functions of graphene nanosheets (GNSs), enabled by the successful use of organic nanocarbon sources (ONCS) as GNS precursors. Through the synergistic integration of ONCS, silica nanoparticles, and SDBS micelles, precise control over GNS morphology is achieved, yielding 1D hollow spheres, 2D holey nanosheets, and 3D sieves. The interfacial interactions and geometric effects of the Tri‐NPs system endow controllability over GNS morphology, layer thickness, and crystallinity. Notably, the nanoreactor effect elevates the degradation temperature and induces gradient molecular pyrolysis during calcination—key to GNS synthesis—thus establishing a novel bottom‐up strategy for GNS‐based materials. When applied as anodes in large‐ion batteries (potassium‐ion and sodium‐ion batteries), the 1D GNS‐hollow spheres exhibit exceptional performance, including long‐term stable cycling at ultra‐high current density. This outperformance is attributed to the loose GNS stacking, hollow nanostructure, and polar chemical bonds that facilitate ion adsorption. While this work provides new insights into controllable GNS synthesis and their application as anodes in large‐ion batteries, it is essential to acknowledge the remaining challenges that must be addressed for future practical implementation and commercialization. For instance, source for preparation of ONCS such as Labrafac WL 1349 and Kolliphor ELP require a chemical industrial formulation as a replacement to reduce costs from several hundred thousand dollars to several thousand dollars. Additionally, a microfluidic system should be designed to maintain homogeneous UV‐polymerization at a scale of tons per day. Second, optimizing electrode density is another key challenge to overcome. The loose stacking of GNSs, which facilitates excellent ion diffusion and cycling stability, also results in relatively low electrode density. Balancing the loose stacking structure (critical for efficient ion transport) and high electrode density (necessary for superior volumetric performance) demands further optimization of GNS assembly methods, as well as the introduction of appropriate binders or composite components. Future research should focus on resolving these challenges, specifically exploring scalable synthesis strategies and developing density‐tunable GNS architectures. Addressing these limitations will further advance the Tri‐NPs‐mediated bottom‐up strategy, unlocking the full potential of GNS‐based materials in large‐ion batteries and other emerging energy storage applications.

## Conflicts of Interest

The authors declare no conflicts of interest.

## Ethics Statement

This research does not involve human participants, human data, or human tissue. All chemical reagents used in the experiments were handled in accordance with standard laboratory safety procedures.

## Supporting information




**Supporting File 1**: advs75661‐sup‐0001‐SuppMat.docx.

**Supporting File 2**: advs75661‐sup‐0002‐Python_Stacking_GNS.zip.

**Supporting File 3**: advs75661‐sup‐0003‐Videos.zip.

## Data Availability

The data that support the findings of this study are available from the corresponding author upon reasonable request.
