# Supplementary material for: A Triple‐Nanoparticle System for Controlled Graphene Nanosheet Stacking: Enabling K/Na‐Ion Battery Anodes with Ultra‐Fast Charging Exceeding Petroleum Vehicle Refueling
Source: Adv Sci (Weinh). 2026 May 10;13(44):e24370. doi: 10.1002/advs.202524370 (PMC13335946; doi:10.1002/advs.202524370)
Supplement: Supplementary file 1 [file ADVS-13-e24370-s001.docx]

**A Triple-Nanoparticle System for Controlled Graphene Nanosheet Stacking: Enabling K/Na-Ion Battery Anodes with Ultra-Fast Charging Exceeding Petroleum Vehicle Refueling**

Shukai Ding^1*^, Le Zhang^1,2, ┼^, Hang Li^1,2,┼^, Yang Zhang^3^, Guoquan Suo^2*^, Bin Han^1^, Dongfeng Sun^1^, Wenqi Zhao^1^, Gaohui Du^1^, Qingmei Su^1^, Bingshe Xu^1,4,5^, Zhen Xu^3*^, Wei Wang^6*^

^1^ School of Physics & Information Science, Shaanxi University of Science and Technology, Xi’an 710021, China

^2^ School of Materials Science & Engineering, Shaanxi University of Science and Technology, Xi’an 710021, China

^3^ School of Chemistry and Chemical Engineering, Northwestern Polytechnical University, Xi'an 710072, China

^4^ Key Laboratory of Interface Science and Engineering in Advanced Materials, Ministry of Education, Taiyuan University of Technology, Taiyuan, Shanxi 030024, China

^5^ Shanxi-Zheda Institute of Advanced Materials and Chemical Engineering, Taiyuan, Shanxi 030024, China

^6^ School of Metallurgical and Ecological Engineering, University of Science and Technology Beijing, Beijing, 100083, China,

* Corresponding author:

A/Prof. Shukai Ding, Email: [dingshukai@sust.edu.cn](mailto:dingshukai@sust.edu.cn);

A/Prof. Guoquan Suo, Email: guoquansuo@sust.edu.cn;

Prof. Zhen Xu, Email: zhen1@nwpu.edu.cn;

Prof. Wei Wang, Email: [wwang@ustb.edu.cn](mailto:wwang@ustb.edu.cn);

^The ┼ symbol represents that these authors made equal contributions.^

***Methods***

***Materials:*** Labrafac WL 1349 (Gattefosse S.A., France), Kolliphor ELP (BASF, Germany). Tri-(propylene glycol) diacrylate (TPGDA), 1-Hydroxycyclohexyl phenyl ketone (HCPK, 99 % purity), 3,4,9,10-Perylenetetracarboxylic dianhydride (PTCDA), and sodium dodecyl benzene sulfonate (SDBS, Macklin Biochemical Co., Ltd., China), and SiO_2_ nanoparticles were purchased by Brofos Nanotechnology (Ningbo) Co., Ltd. All reagents used in testing batteries were purchased by DodoChem Co. Commercial sodium ion cathode of Na_3_V_2_(PO4)_3_ were purchased by Jiangsu Yilimao Co.. All experiments were performed with Milli-Q water.

**Synthesis of ONCS:** Firstly, tripropylene glycol diacrylate monomer (TPGDA), 1-hydroxycyclohexyl phenyl ketone (HCPK) (5 wt.% based on TPGDA), medium-chain triglycerides (Labrafac WL 1349) and polyoxyethylene castor oil (Kolliphor ELP) were quickly and uniformly mixed using a vortex apparatus at a ratio of TPGDA: Labrafac WL 1349: Kolliphor ELP = 0.8:0.8:1 (mass ratio) for an oil phase. Then, a certain amount of deionized water was quickly poured into the oil phase in a ratio of oil phase: water phase = 4:3 (mass ratio) and vortexed to form a nanoemulsion. Finally, the nanoemulsion was UV-polymerized by silica tube using a UV point light source and a syringe pump at a rate of 0.2 ml/min, and the excess Kolliphor ELP was removed by dialysis to obtain an ONCS suspension.

**Preparation of GNS-based materials by Tri-NPs system:** The mass ratio of SiO_2_ nanoparticles at different sizes (20, 100, and 500 nm) to ONCS was kept at 1:20, and the mass ratio of sodium dodecylbenzene sulfonate (SDBS) to ONCS was kept at 1:50 to prepare all GNS architectures except special explanation. SiO_2_ nanoparticles and SDBS were uniformly dispersed in the ONCS suspension according to the above ratio, and freeze-dried to remove water. The dried gel was heated to 800 ºC at 5 ºC/min in a tubular furnace and calcined in an argon atmosphere for 2 h. Subsequently, the calcined black powder was placed in a hydrofluoric acid aqueous solution for 24 h to remove SiO_2_ nanoparticles, and finally washed with water until the pH value was neutral, and dried in a vacuum drying oven at 80 ºC to obtain the final product.

**Characterization and physicochemical properties:** XRD (Bruker D8 Advance, Cu Kα radiation (λ = 1.5406 Å)), Raman spectrum (Renishaw InVia, He−Ne laser (532 nm)), XPS (Thermo Scientific ESCALAB 250Xi), TGA (Netzsch STA 449F3, 10 °C/min up to 1000 °C in air environment), N2 adsorption/desorption (ASAP 2460 volumetric sorption analyzer), Brunauer-Emmett-Teller (BET) surface area is calculated from the isotherm by the BET equation. Pore size distribution is calculated according to the Barrett-Joyner-Halenda (BJH) model. TEM (JEOL JEM-2100 plus, accelerating 200 kV voltage), NMR (Bruker Avance NEO 600MHz) and SEM (JEOL JIB-4700F) are implemented for the characterization of physicochemical properties. Fourier Transform Infrared (FTIR) spectra of the samples were obtained using the KBr pellet method by a Bruker Vector 22 FTIR spectrometer with a resolution of 0.07 cm^-1^.

Gas Chromatography-Mass Spectrometry (GC-MS) is performed using an Agilent 7890B gas chromatograph equipped with a mass spectrometry detector. A HP-l capillary column is used for component separation, and compound identification is conducted based on the NIST 14 mass spectral database. Instrumental parameters for the analysis were as follows:

| Injection Port Temperature | 300 ℃ |
| --- | --- |
| Iniection Volume | 0.1uL |
| Split Ratio | 1:10 |
| Carrier Gas | Helium |
| Initial Oven Temperature | 50 ℃ |
| Initial Hold Time | 5 min |
| Temperature Ramp Rate | 10 ℃ min^−1^ |
| Final Oven Temperature | 320 ℃ |
| Final Hold Time | 5 min |
| Mass Scan Range (m/z) | 50-750 |

**Electrochemical measurements:** For anode testing, the electrode slurry is obtained by mixing active compound, carbon black, and polyvinylidene fluoride (PVDF) binder at the weight ratio of 7:2:1 in N-methyl pyrrolidone (NMP), and is coated and dried on Cu foil collector by the vacuum drying at 100 ℃ for 10 h for the electrode. For cathode testing, the electrode slurry is obtained by mixing active compound, carbon black, and polyvinylidene fluoride (PVDF) binder at the weight ratio of 7:2:1 in N-methyl pyrrolidone (NMP), and is coated and dried on Al foil collector by the vacuum drying at 100 ℃ for 10 h for the electrode.

Without special instruction, the CR-2032 coin-type half-cell is assembled with the potassium metal as the counter electrode and 3 M KTFSI in DME as the electrolyte in a glove box for PIBs, the sodium plate the counter electrode and 1M NaClO_4_ in DEC:EC= 1:1 Vol % with 5% FEC for SIBs, respectively. The CR-2032 coin-type full-cell is assembled by N/P ratio of 1.05. The cell is tested by a battery-testing system (Neware Co. Ltd, Shenzhen) under the voltage range from 0.01 to 2.6 V at a constant temperature of 25 ºC. For high current density, testing is implemented after 10 cycles at 50 mA g^-1^ to stabilize the solid electrolyte interface (SEI). Cyclic voltammograms is conducted by an electrochemical workstation (Shanghai Chenhua Co. Ltd., China) with a different scan rate of 0.2 ─ 1 mV s^−1^. Electrochemical impedance spectroscopy (EIS) is performed from 10^−2^ to 10^5^ Hz. All results are analyzed from the average value of three samples at least. The average mass loading of active materials is kept at around 1 mg cm^-2^ in one cell.

**Figure S1** Characterization of 3D GNS-sieves prepared by Tri-NPs system.

**Figure S2** Characterization of 1D GNS-hollow sphere prepared by Tri-NPs system.

**Figure S3** Characterization of 2D GNS-holey nanosheet prepared by Tri-NPs system.

**Figure S4** Depiction and characterization of series of GNS-materials prepared by Dual-NPs systems (a and d) 3D GNS-sieve, (b and e) 1D GNS-hollow sphere, and (c, f) 1D GNS-nanotube.

**Figure S5** Characterization of 3D GNS-sieve prepared by Dual-NPs system.

**Figure S6** Comparison of 3D GNS-sieve prepared by (a) Dual-NPs system and (b) Tri-NPs system.

**Figure S7** Characterization of 1D GNS-hollow sphere prepared by Dual-NPs system.

**Figure S8** Characterization of 1D GNS-nanotube prepared by Dual-NPs system.

**Figure S9** Characterization of 2D GNS-holey nanosheet prepared by Tri-NPs system (a-c) before HF etching (d-f) after HF etching, 1D-GNS nanotube prepared by Dual-NPs system (g-i) before HF etching, and (j-l) after HF etching.

**Figure S10** Characterization of carbon nanomaterials prepared by Dual-NPs system composing of SDBS micelle and SiO_2_ nanoparticles (100 nm).

**Figure S11** Characterization of carbon nanomaterials prepared by Dual-NPs system.

**Figure S12** Characterization of carbon materials prepared by calcination of pure SDBS micelle for (a-d) the concentration is same as in Tri-NPs system, and (e-h) the concentration of SDBS at 10 times.

**Figure S13** Characterization of carbon nanomaterials prepared by Dual-NPs system (a-c) the same concentration as others experiments and (d-f) the concentration of SDBS at 10 times.

**Figure S14** (a-b) ONCS direct characterized by TEM and (c-d) ONCS characterized by TEM after ethanol dialysis.

**Figure S15** Molecular analysis of GC-MS spectrum in **Figure 2b** (**main text**) of calcinated ONCS, calcinated Labrafac WL 1349, and calcinated CTN.

**Figure S16** FTIR spectrum of calcinate at different calcination temperature (the label "calcinate-number" denotes the calcinate obtained after calcination at the corresponding temperature (e.g., Calcinate-600 represents the sample calcined at 600 ℃)).

**Figure S17** ^1^H NMR of Labrafac WL 1349.

**Figure S18** ^1^H NMR of monomer TPGDA.

**Figure S19** Pyrolysis reaction of labrafac WL 1349 during 200 ℃.

**Table S1** Evolution of ¹H NMR spectra of ONCS extracts during calcination.

| Protons  Ratio of H_Area_ | 3,26 | 4,5 | Methylene protons after polymerization | 6,7 | acetic acid | 8,9,10 | aliphatic protons | 1 |
| --- | --- | --- | --- | --- | --- | --- | --- | --- |
| ONCS-fresh-extract | 0.029 | 0.030 | 0.30 | 0.09 | / | 0.09 | 0.36 | 0.1 |
| ONCS-200-extract | 0.02 | 0.025 | 0.28 | 0.06 | 0.14 | 0.06 | 0.31 | 0.1 |

**Figure S20** Micro-polariscope photographs of ONCS.

**Laplace pressure calculation:**

Laplace's law for a sphere states:

$$\Delta P= \frac{r}{2\gamma}$$

If initial state radius=r_1_ → Final state radius=r_2_

The pressure-increase ratio is:

$$\Delta P_{i}= \frac{\Delta P_{1}}{\Delta P_{2}}=\frac{r_{1}}{r_{2}}$$

**Figure S21** Calculated pressure-increase ratio of the sphere at radius from 1000 to 1 nm.

**Figure S22** (a) Cycling performance of GNS-based materials prepared by different nanoparticles system at 50 mAh g^-1^ and (b) Magnification scale of **Figure a**.

**Figure S23** Cycling and rate performance of different GNS-based materials anodes prepared by Tri-NPs system in current density of (a) 500 mA g^-1^ and (b) 1000 mA g^-1^.

**Table S2** Comparison of charge time for different carbonaceous materials in PIBs

| Specific capacity (mAh g^-1^) | Current density (mA g^-1^) | Number of cycles | Charge time (min) | Reference |
| --- | --- | --- | --- | --- |
| 140 | 500 | 500 | 16.8 (3.57C) | ^5^ |
| / | 1C | 1000 | 60 | ^6^ |
| 121 | 100 | 100 | 72.6 (0.8C) | ^7^ |
| 212 | 55.8 | 100 | 228 (0.26C) | ^8^ |
| 100.3 | 200 | 100 | 30 (2C) | ^9^ |
| 230 | 200 | 1000 | 69 (0.87C) | ^10^ |
| <200 | 1C | 500 | 60 | ^11^ |
| <250 | 100 | 400 | 150 (0.4C) | ^12^ |
| <250 | 100 | 200 | 150 (0.4C) | ^13^ |
| 240 | 558 | 1000 | 24 (2.41C) | ^14^ |
| 258.2 | 2000 | 6000 | 7.75 (7.7C) | ^15^ |
| 188 | 3000 | 1250 | 3.76 (15.96C) | This work |

**Figure S24** Voltage vs specific capacity curve of 1D GNS-hollow sphere in PIBs in current density of 3000 mA g^-1^ and SIBs in current density of 2000 mA g^-1^, respectively.

**Figure S25** (a) Structure of half-cell in PIBs for in-situ cycling experiment and (b) photograph cell.

***Electrochemical mechanism of 1D GNS-hollow sphere and 2D GNS-holey nanosheet in PIBs***

**Figure S26** Comparison of 2D GNS-holey nanosheet and 1D GNS-hollow sphere by (a) Raman spectrum, (b) XRD pattern, (c) survey XPS spectrum, (d) C1s XPS spectrum, (e) absorption and desorption curve of N_2_ gas, (f) pore width distribution, and cyclic voltammograms at various sweep rates, contribution ratio of the capacitive and diffusion-controlled process at various sweep rates, and separation of the total current and capacitive currents at 0.2 mV s^-1^ for 1D GNS-hollow sphere (g-i) and for 2D GNS-holey nanosheet (j-l).

**Figure S27** (a, b) Determination of the b value using the relationship between the peak current and the sweep rate of 1D GNS-hollow sphere and 2D GNS-holey nanosheet, respectively, and (c) ESI for the 1D GNS-hollow sphere and 2D GNS-holey nanosheet at fresh state and 10^th^ cycle.

**Formula**

Formula S1.

$i={av}^{b}$

Formula S2.

$$\log\left( i \right)=loga+blog(v)$$

Formula S3.

$$i=k_{1}v{+ k}_{2}v^{1/2}$$

Formula S4.

$$i/{v^{1/2}}={k_{1}v}^{1/2}+k_{2}$$

*i─*Current (mA)

*ν─*Sweep rate (mV s^-1^)

*k_1_─*Constant for the capacitive-controlled process

*k_2_─*Constant for the diffusion-controlled process

**Table S3** D and G peak information in Raman spectrum for different discharge and charge state of 1D GNS-hollow sphere in half-cell at first cycle.

|  | D Peak Position | Intensity | G Peak Position | Intensity | ID/IG |
| --- | --- | --- | --- | --- | --- |
| 1V-discharge | 1351.7 | 66.6 | 1587.3 | 73.3 | 0.909 |
| 0.5V-discharge | 1347.7 | 98.5 | 1596.8 | 105.6 | 0.933 |
| 0.05V-discharge | 1347.7 | 89.6 | 1595.7 | 95.0 | 0.943 |
| 0.3V-charge | 1346.6 | 188.3 | 1592.6 | 206.6 | 0.912 |
| 0.5V-charge | 1348.7 | 106.9 | 1593.7 | 113.1 | 0.945 |
| 2.7V-charge | 1343.5 | 82.3 | 1593.7 | 90.5 | 0.909 |

**Table S4** D and G peak information in Raman spectrum for different discharge and charge state of 1D GNS-hollow sphere in half-cell after 10 cycles

|  | D Peak Position | Intensity | G Peak Position | Intensity | I_D_/I_G_ |
| --- | --- | --- | --- | --- | --- |
| Fresh | 1348.4 | 73.5 | 1581 | 70.6 | 1.041 |
| 1V-discharge | 1349.8 | 85.7 | 1590.6 | 87.4 | 0.980 |
| 0.5V-discharge | 1342.3 | 96.68 | 1589.5 | 108.4 | 0.892 |
| 0.05V-discharge | 1348.9 | 78.2 | 1590.6 | 84.4 | 0.927 |
| 0.3V-charge | 1346.2 | 69 | 1588.55 | 70.4 | 0.98 |
| 0.5V-charge | 1336.62 | 67.65 | 1584.9 | 70.32 | 0.962 |
| 2.7V-charge | 1349.84 | 70.86 | 1585.81 | 71.1 | 0.997 |

**Figure S28** comparison of electrochemical performance of 1D GNS-hollow sphere with Carbon quantum dots,^5^ Soft carbon,^6^ Carbon microspheres,^7^ Graphitic carbon nanocages,^8^ Activated carbon,^16^ Porous carbon microspheres,^17^ graphene microspheres,^10^ and rGO aerogel.^18^

***Simulation for GNS stacking***

Python programs are design to simulate the stacking of GNS for unveiling the topological geometry structure. Based on the experimental crystalline and structure, the GNS model compose of 135 atoms at 1.842*1.702 nm in **Figure S29**. The program named by graphene_sheet_stacking.py stack the GNS onto a spherical surface at the diameter of 32.5 nm. Herein, the GNS is treated as a rigid sheet, modeling their accumulation with a focus on rigid-body alignment and overlap avoidance.

In detail, using a Fibonacci sphere algorithm, it generates uniformly distributed points on the sphere's surface as potential placement sites. Each GNS is rigidly aligned to the sphere's normal at these points using rotation matrices derived from Rodrigues' formula. The simulation checks for overlaps between sheets using a combination of centroid distance and KDTree-based atom-atom distance queries, ensuring a threshold-based precision (> 0.35 nm for the Van der Waals radius). It calculates the spherical projected area of each valid patch and tracks total surface coverage until reaching 105% of the sphere's area. Finally, it visualizes the 3D model with cross-sections and exports patch coordinates to a graphene_patches.CSV file for further analysis. The 3D model can be checked by 3D_view_for_graphene_sheet_stacking.py.

Then, the GrapheneAnalyzer class in Calculating_Intercrossion_Line.py analyzes GNS placements on spherical surfaces using parallel computing. It loads the data in graphene_patches.CSV file for rebuilding the 3D GNS model. It then creates evenly spaced 20 sampling points on the sphere’s equatorial plane (Y=0) in Figure 3c. For each sampling point (e.g., (X₀, 0, Z₀)), the program defines a square region on the XZ plane centered at (X₀, Z₀) with dimensions -2.5 to +2.5 nm in both X and Z directions (total size: 5×5 nm). This region is set by the analysis_size parameter (default: 2.5 nm, leading to a 5×5 nm area). The program checks if carbon-carbon bonds cross the XZ plane within the 5×5 nm region. For each bond (line segment between two atoms), it calculates the intersection point if the bond straddles Y=0, gives the 3D graph in folder of graphene_plots. Each sampling point is plotted on the XZ plane in **Figure S31**, showing intersection lines colored by graphene patch. A gray square outlines the 5×5 nm area, and a dashed orange arc represents the sphere’s circumference for spatial context.

**Figure S29** Simulated GNS by GOPY.py.^19^

**Figure S30** simulated stacking GNS with the Van der Waals radius limit in 2D Plane

**Figure S31** Calculated distance of two GNS in 20 random 5×5 nm XZ areas.

**Figure S32** Cycling performance of 1D GNS-hollow sphere in PIBs at current density of 3000 mA g^-1^ and in SIBs at current density of 2000 mA g^-1^, respectively for the active materials loading of 2 mg cm^-2^.

**Figure S33** Voltage-capacity curve of (a) PTCDA in the half cell of PIBs, (b) Na_3_V_2_(PO_4_)_3_ in the half cell of SIBs, (c) full cell of 1D GNS-hollow sphere and PTCDA at N/P of 1.05 in PIBs, and (d) full cell of 1D GNS-hollow sphere and Na_3_V_2_(PO_4_)_3_ at N/P of 1.05 in PIBs at current density of 10 mA g^-1^.

**Reference**

(1)	Acik, M.; Lee, G.; Mattevi, C.; Pirkle, A.; Wallace, R. M.; Chhowalla, M.; Cho, K.; Chabal, Y. The Role of Oxygen during Thermal Reduction of Graphene Oxide Studied by Infrared Absorption Spectroscopy. *J. Phys. Chem. C* **2011**, 19761–19781.

(2)	Mo, Y.; Zhou, W.; Wang, K.; Yang, W.; Liu, Z.; Chen, S.; Gao, P.; Liu, J. Uncovering the Role of Organic Species in the SEI on Graphite for Fast K+ Transport and Long-Life Potassium-Ion Batteries. *Energy Environ. Sci.* **2024**, *18*, 1418–1427. https://doi.org/10.1039/d4ee04698f.

(3)	Chi, C.; Liu, Z.; Wang, G.; Qi, B.; Qiu, Z.; Yan, Y.; Huangfu, C.; Lu, X.; Yang, X.; Gong, M.; Cao, K.; Wei, T.; Fan, Z. Graphene Oxide Block Derived Edge-Nitrogen Doped Quasi-Graphite for High K+ Intercalation Capacity and Excellent Rate Performance. *Adv. Energy Mater.* **2023**, *13* (46), 2302055. https://doi.org/10.1002/aenm.202302055.

(4)	Zhang, K. Y.; Liu, H. H.; Su, M. Y.; Yang, J. L.; Wang, X. T.; Huixiang Ang, E.; Gu, Z. Y.; Zheng, S. H.; Heng, Y. L.; Liang, H. J.; Wang, Y.; Li, S.; Wu, X. L. Defect Engineering Unveiled: Enhancing Potassium Storage in Expanded Graphite Anode. *J. Colloid Interface Sci.* **2024**, *664*, 607–616. https://doi.org/10.1016/j.jcis.2024.03.084.

(5)	Guo, Y.; Feng, Y.; Li, H.; Wang, Y.; Wen, Z.; Zhou, G. Carbon Quantum Dots in Hard Carbon: An Approach to Achieving PIB Anodes with High Potassium Adsorption. *Carbon N. Y.* **2022**, *189*, 142–151. https://doi.org/10.1016/j.carbon.2021.12.038.

(6)	Liu, Y.; Lu, Y. X.; Xu, Y. S.; Meng, Q. S.; Gao, J. C.; Sun, Y. G.; Hu, Y. S.; Chang, B. B.; Liu, C. T.; Cao, A. M. Pitch-Derived Soft Carbon as Stable Anode Material for Potassium Ion Batteries. *Adv. Mater.* **2020**, *32* (17), 2000505. https://doi.org/10.1002/adma.202000505.

(7)	Wang, S.; Li, Y.; Ma, F.; Wu, X.; Zhou, P.; Miao, Z. Phenolic Resin-Based Carbon Microspheres for Potassium Ion Storage. *Appl. Surf. Sci.* **2020**, *506* (November 2019), 144805. https://doi.org/10.1016/j.apsusc.2019.144805.

(8)	Cao, B.; Zhang, Q.; Liu, H.; Xu, B.; Zhang, S.; Zhou, T.; Mao, J.; Pang, W. K.; Guo, Z.; Li, A.; Zhou, J.; Chen, X.; Song, H. Graphitic Carbon Nanocage as a Stable and High Power Anode for Potassium-Ion Batteries. *Adv. Energy Mater.* **2018**, *8* (25), 1801149. https://doi.org/10.1002/aenm.201801149.

(9)	Tai, Z.; Zhang, Q.; Liu, Y. Y.; Liu, H. H.; Dou, S. Activated Carbon from the Graphite with Increased Rate Capability for the Potassium Ion Battery. *Carbon N. Y.* **2020**, *189* (17), 144805. https://doi.org/10.1016/j.carbon.2017.07.041.

(10)	Qingfeng Zhang; Cheng, X.; Wang, C.; M.Rao, A.; Lu, B. Sulfur-Assisted Large-Scale Synthesis of Graphene Microspheres for Superior Potassium-Ion Batteries. *Energy Environ. Sci.* **2021**, *14*, 965–974. https://doi.org/10.1039/D0EE03203D.Energy.

(11)	Bin, D.; Lin, X.; Sun, Y.; Xu, Y.; Zhang, K.; Cao, A.; Wan, L.; Bin, D.; Lin, X.; Sun, Y.; Xu, Y.; Zhang, K.; Cao, A. Engineering Hollow Carbon Architecture for High-Performance K-Ion Battery Anode. *J. Am. Chenical Soc.* **2018**, *140* (23), 7127–7134. https://doi.org/10.1021/jacs.8b02178.

(12)	Ma, X.; Fu, H.; Xia, H.; He, Y.; Zhang, D.; Wu, Y.; Zhou, J.; Zhou, J.; Fan, L.; Lu, B. Synergistic Kinetics Modulation at Graphite Interface Enables Ultrafast and Durable Potassium-Ion Batteries. *Adv. Funct. Mater.* **2025**, 2504576. https://doi.org/10.1002/adfm.202504576.

(13)	Lim, C.; Bong, G.; Ha, S.; Hyo, J.; Myeong, S.; Yu, Y.; Yang, X.; Ouk, S.; Lee, Y. Fluorination Swing Reaction Derived Porous Carbon for Effective Potassium Ion Battery Anode Material. *Carbon N. Y.* **2025**, *234* (January), 120007. https://doi.org/10.1016/j.carbon.2025.120007.

(14)	Gao, Y.; Ma, X.; Yan, Y.; Zhang, S.; Liang, J.; Li, B. Unleashing the Underestimated Rate Capability of Graphite Anode for Potassium-Ion Batteries by Sn ( OTf ) 2 Electrolyte Additive. *Adv. Energy Mater.* **2025**, 2404913. https://doi.org/10.1002/aenm.202404913.

(15)	Jiang, Y.; Xiao, N.; Yu, K.; Qiu, J. N-Doped Porous Graphite-like Carbon Armored with Dense Amorphous Shell through a Trojan Horse Strategy for High Performance Potassium-Ion Battery Anode. *Small* **2025**, 07641.

(16)	Tai, Z.; Zhang, Q.; Liu, Y.; Liu, H.; Dou, S. Activated Carbon from the Graphite with Increased Rate Capability for the Potassium Ion Battery. *Carbon N. Y.* **2017**, *123*, 54–61. https://doi.org/10.1016/j.carbon.2017.07.041.

(17)	Ho, S.; Baucom, J.; Li, X.; Shen, L.; Seong, Y.; Sub, I.; Ju, Y.; Na, Y.; Jin, H.; Lu, Y. Porous Carbon Microspheres with Highly Graphitized Structure for Potassium-Ion Storage. *J. Colloid Interface Sci.* **2020**, *577*, 48–53. https://doi.org/10.1016/j.jcis.2020.05.051.

(18)	Liu, L.; Lin, Z.; Chane-Ching, J. Y.; Shao, H.; Taberna, P. L.; Simon, P. 3D RGO Aerogel with Superior Electrochemical Performance for K – Ion Battery. *Energy Storage Mater.* **2019**, *19*, 306–313. https://doi.org/10.1016/j.ensm.2019.03.013.

(19)	Muraru, S.; Burns, J. S.; Ionita, M. GOPY: A Tool for Building 2D Graphene-Based Computational Models. *SoftwareX* **2020**, *12*, 100586. https://doi.org/10.1016/j.softx.2020.100586.
