# Supplementary material for: A Triple‐Nanoparticle System for Controlled Graphene Nanosheet Stacking: Enabling K/Na‐Ion Battery Anodes with Ultra‐Fast Charging Exceeding Petroleum Vehicle Refueling
Source: Adv Sci (Weinh). 2026 May 10;13(44):e24370. doi: 10.1002/advs.202524370 (PMC13335946; doi:10.1002/advs.202524370)
Supplement: Supplementary file 3 [file ADVS-13-e24370-s003.zip › Python_Stacking_GNS(Multi-layer)/ReadMe.docx]

# Python_Stacking_GNS Program Description

## Program Functions

## Calculating_Intercrossion_Line.py

A core tool for graphene stacking analysis with parallel computing support. It loads graphene coordinate data, extracts boundary points of graphene patches using either extremum or adjacency methods, identifies atomic bonds and hexagonal rings, calculates intersections between graphene planes and specified planes, and generates various analytical plots and reports. Results (including 3D distributions, intersection lines, and angle distributions) are saved to a designated directory.

## GOPY.py

A library for constructing 2D graphene computational models. It defines an Atom class to represent atomic properties (coordinates, residue info, etc.) and a Typical_Bond class for bond lengths. The key function generate_pristine_graphene generates pristine graphene sheets with specified dimensions and exports them as PDB files, using helper functions to fill hexagonal lattices and format PDB entries.

## graphene_sheet_stacking.py

Simulates graphene sheet stacking on a spherical surface. It reads graphene atom coordinates from files (e.g., graphene_1.687nm.txt), generates uniform spherical surface points via Fibonacci sampling, aligns graphene sheets to the sphere surface using normal vector matching and rotation matrices, checks for overlaps between sheets, and calculates valid stacked configurations. Outputs include valid graphene patches with their coordinates and projected areas.

## 3D_view_for_graphene_sheet_stacking.py

Visualizes the 3D spatial distribution of stacked graphene sheets. It reads stacking results (e.g., graphene_patches.csv) and renders 3D scatter plots to display the position and arrangement of graphene patches on the sphere.

Program Relationships and Plot Generation Workflow

## Data Source：

GOPY.py generates pristine graphene structures (PDB files), which serve as input for graphene_sheet_stacking.py. Alternatively, precomputed graphene coordinates (e.g., graphene_1.687nm.txt, containing PDB-formatted atomic positions) can be directly used.

## Stacking Simulation：

graphene_sheet_stacking.py processes graphene coordinates (from GOPY.py or text files), simulates stacking on a sphere, and outputs results (e.g., graphene_patches.csv) containing patch coordinates and metadata.

## Analysis and Visualization：

Calculating_Intercrossion_Line.py analyzes the stacking results to generate quantitative plots (intersection lines, angle distributions, etc.) in the output directory (default: graphene_analysis_plots).

3D_view_for_graphene_sheet_stacking.py directly visualizes the 3D distribution of patches from the CSV output.

To obtain plots in the plots directory: Run graphene_sheet_stacking.py to generate stacking data, then execute Calculating_Intercrossion_Line.py to produce analytical plots. For quick 3D visualization, run 3D_view_for_graphene_sheet_stacking.py with the stacking results.
