# Supplementary figures and images for: A Triple‐Nanoparticle System for Controlled Graphene Nanosheet Stacking: Enabling K/Na‐Ion Battery Anodes with Ultra‐Fast Charging Exceeding Petroleum Vehicle Refueling
Source: Adv Sci (Weinh). 2026 May 10:e24370. Online ahead of print. doi: 10.1002/advs.202524370 (PMC13335946; doi:10.1002/advs.202524370)

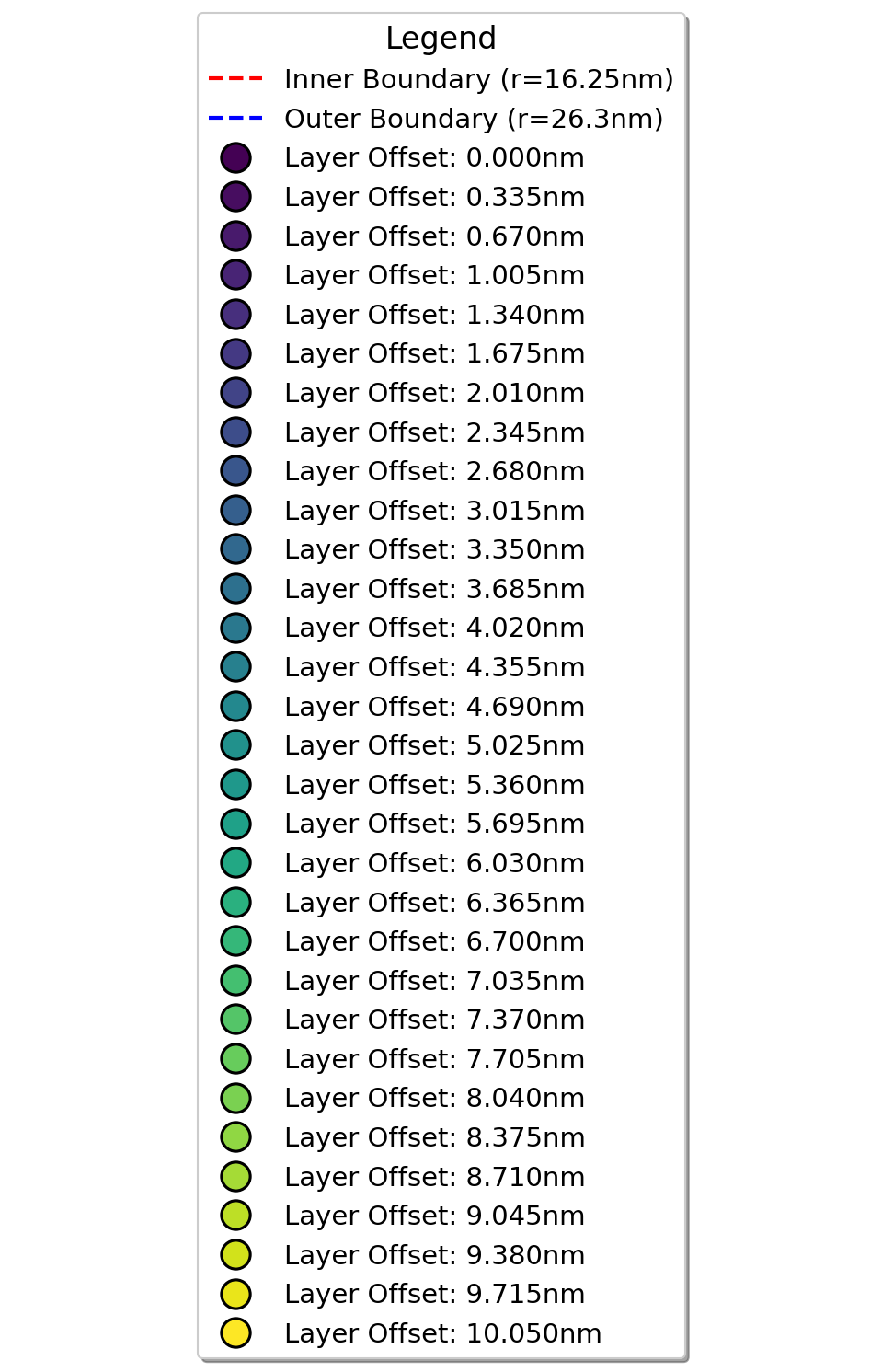

Supplement: Supplementary file 2 — Supporting File 2: advs75661‐sup‐0002‐Python_Stacking_GNS.zip. [file ADVS-9999-e24370-s003.zip › Python_Stacking_GNS(Multi-layer)/graphene_intersection_final_results/2d_distribution_legend.png]

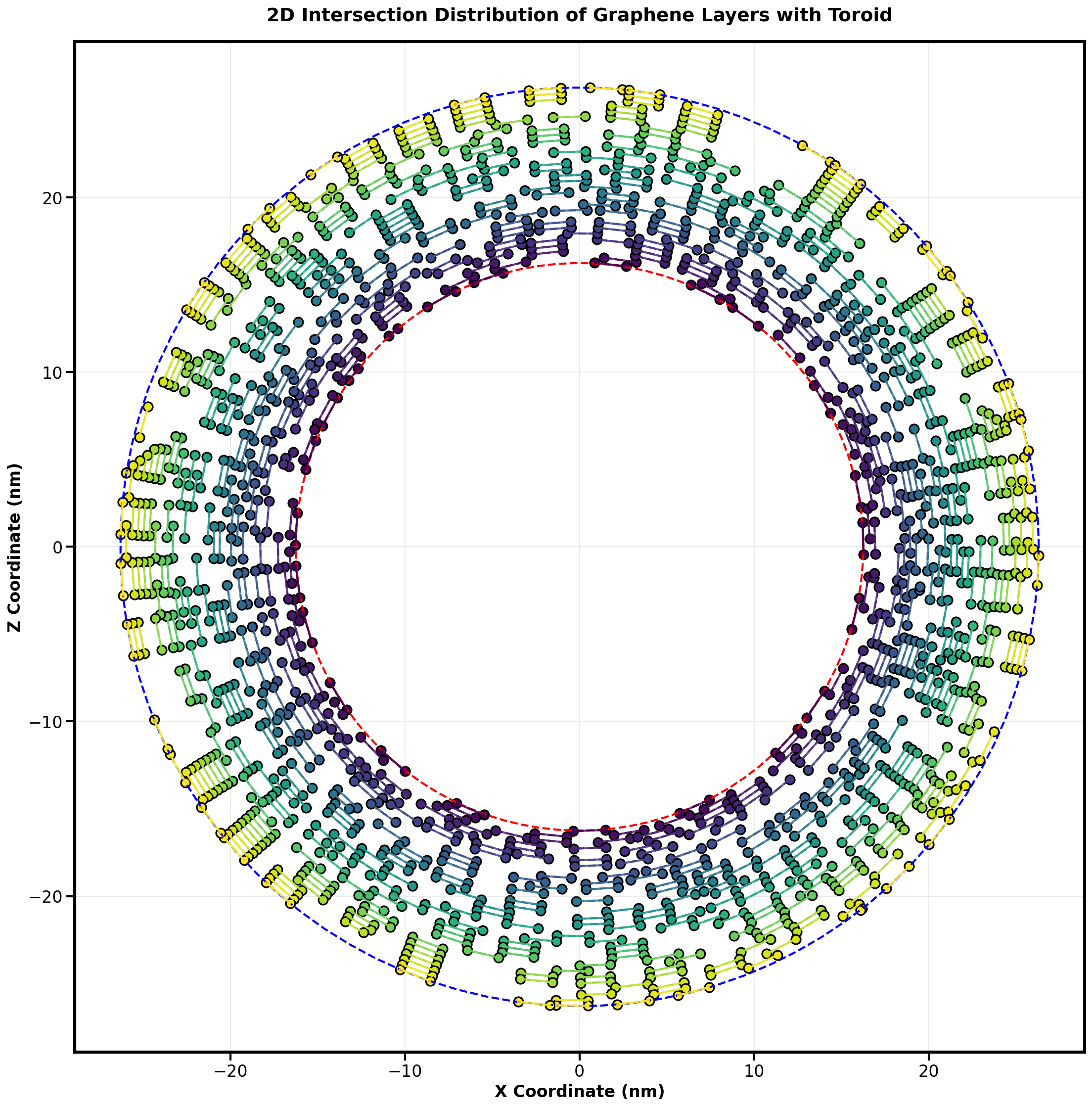

Supplement: Supplementary file 2 — Supporting File 2: advs75661‐sup‐0002‐Python_Stacking_GNS.zip. [file ADVS-9999-e24370-s003.zip › Python_Stacking_GNS(Multi-layer)/graphene_intersection_final_results/2d_distribution_main.png]

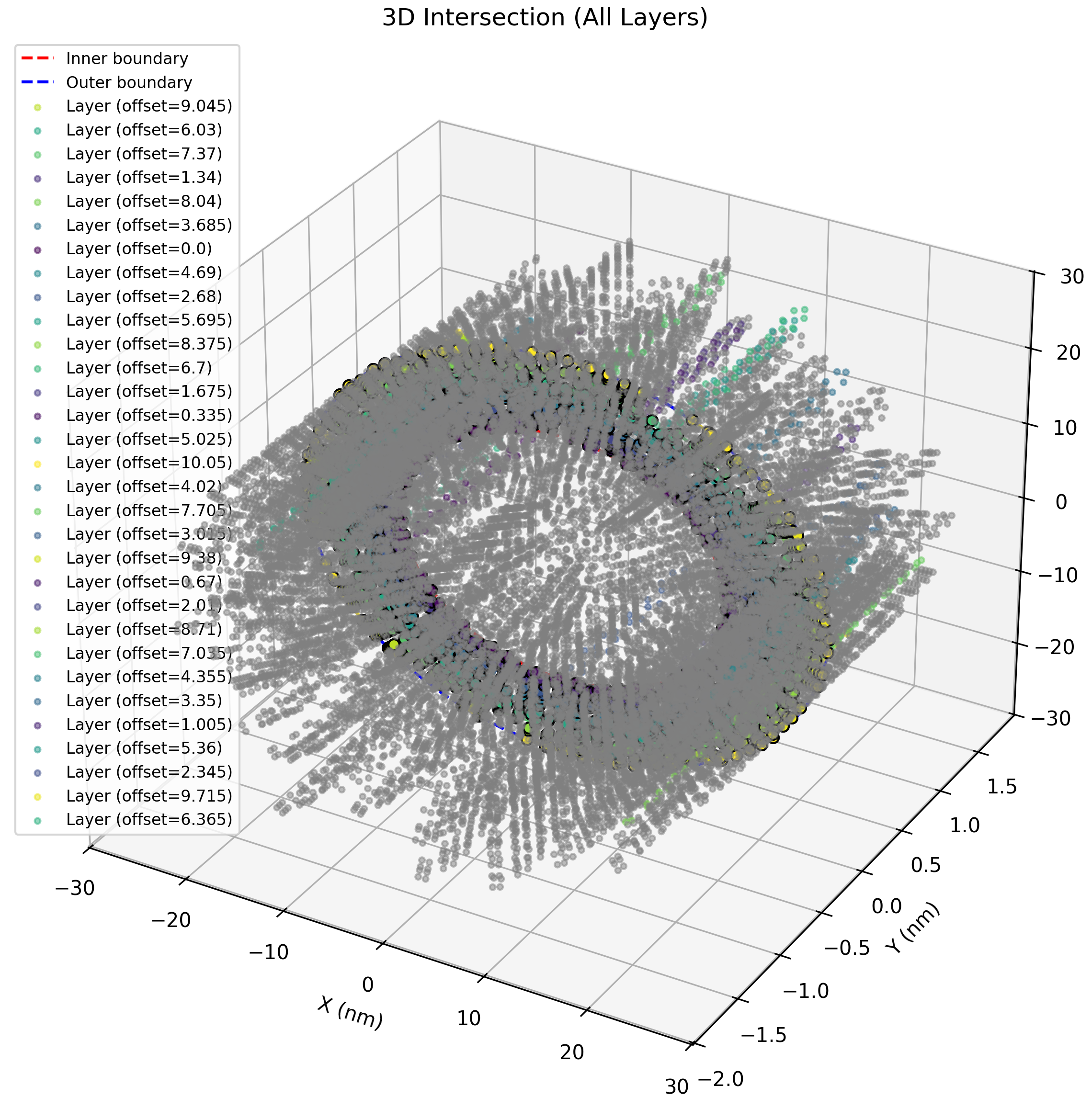

Supplement: Supplementary file 2 — Supporting File 2: advs75661‐sup‐0002‐Python_Stacking_GNS.zip. [file ADVS-9999-e24370-s003.zip › Python_Stacking_GNS(Multi-layer)/graphene_intersection_final_results/3d_visualization.png]

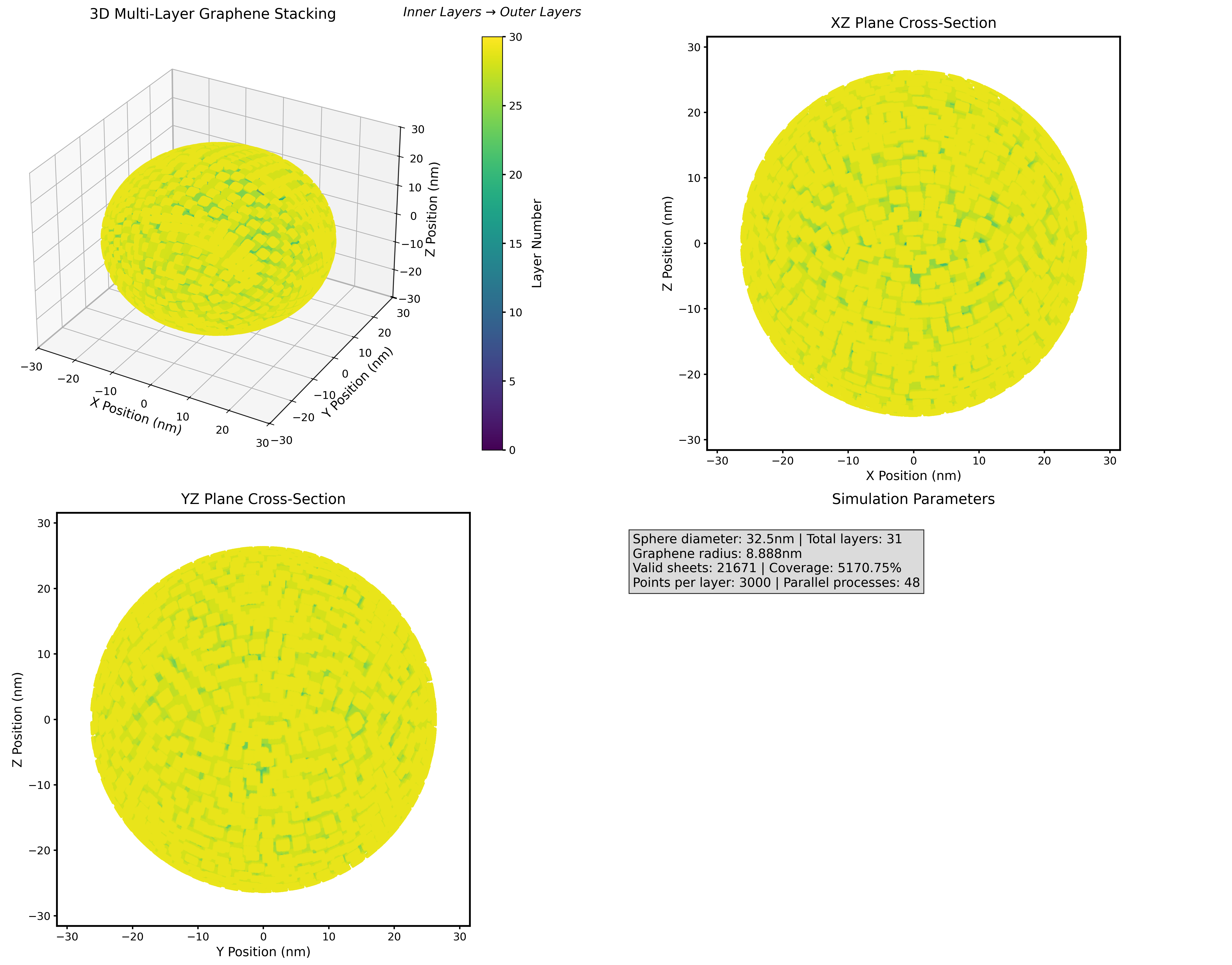

Supplement: Supplementary file 2 — Supporting File 2: advs75661‐sup‐0002‐Python_Stacking_GNS.zip. [file ADVS-9999-e24370-s003.zip › Python_Stacking_GNS(Multi-layer)/graphene_stacking_fibonacci_per_layer.png]

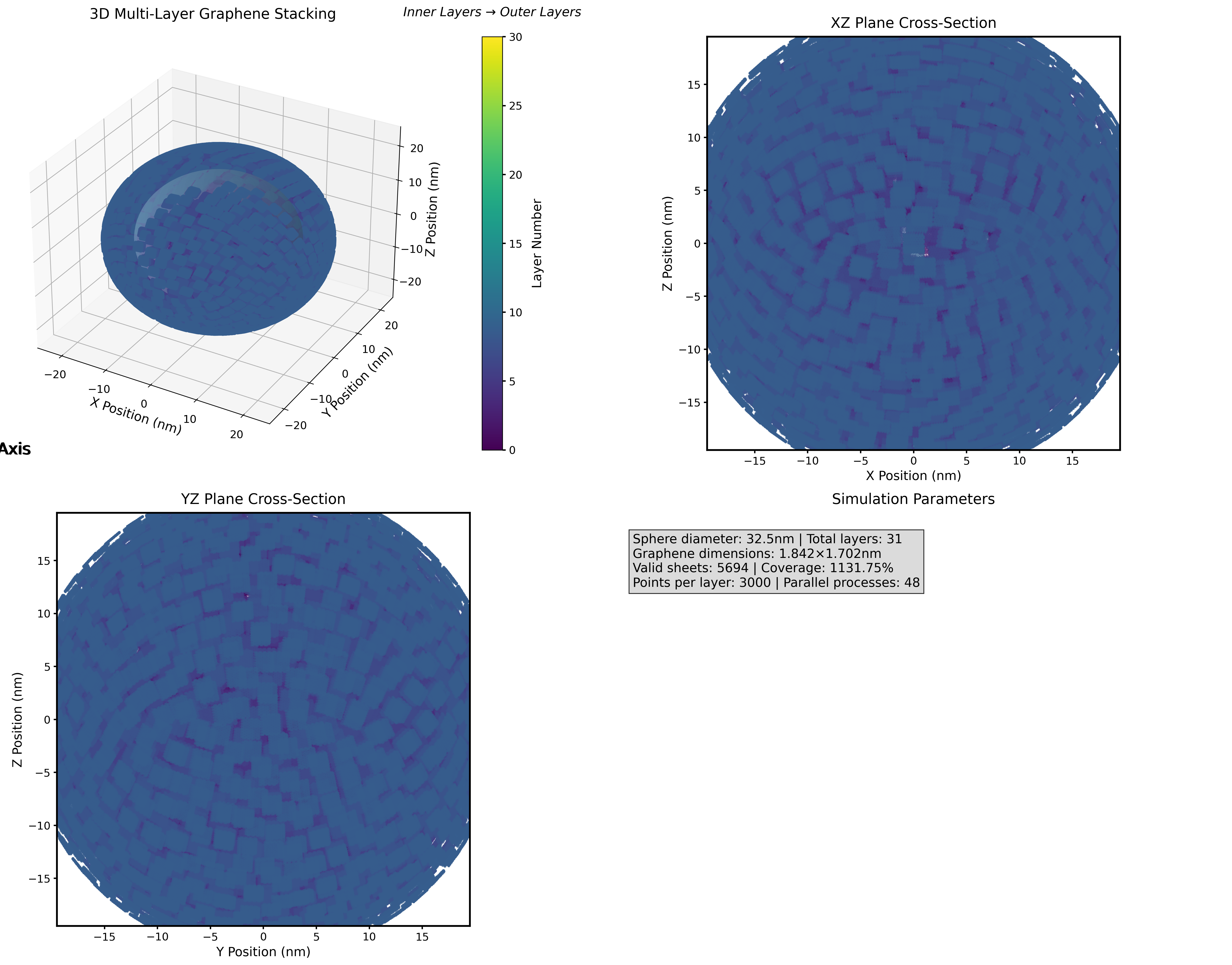

Supplement: Supplementary file 2 — Supporting File 2: advs75661‐sup‐0002‐Python_Stacking_GNS.zip. [file ADVS-9999-e24370-s003.zip › Python_Stacking_GNS(Multi-layer)/graphene_stacking_with_progress.png]

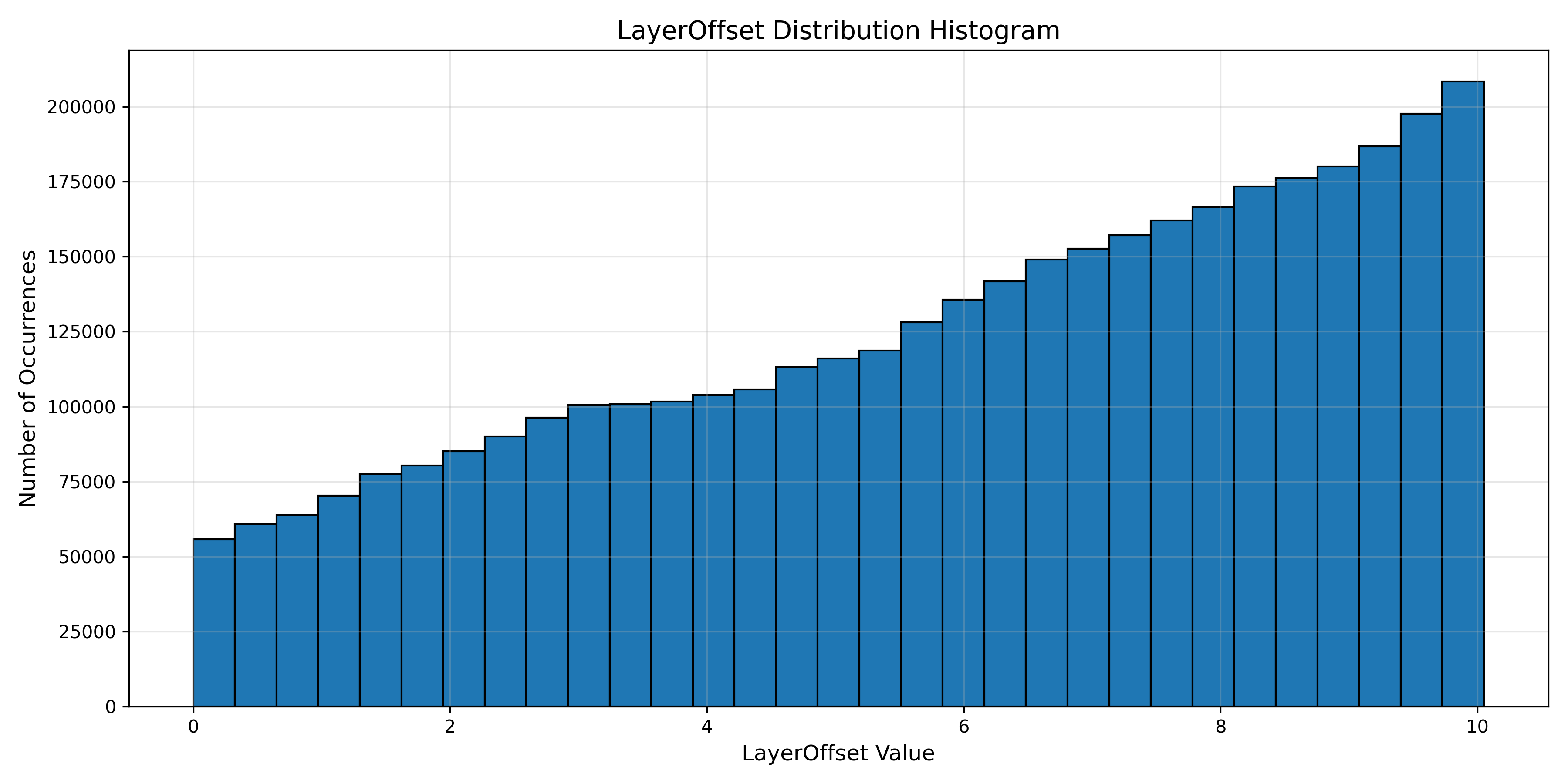

Supplement: Supplementary file 2 — Supporting File 2: advs75661‐sup‐0002‐Python_Stacking_GNS.zip. [file ADVS-9999-e24370-s003.zip › Python_Stacking_GNS(Multi-layer)/layer_offset_distribution.png]

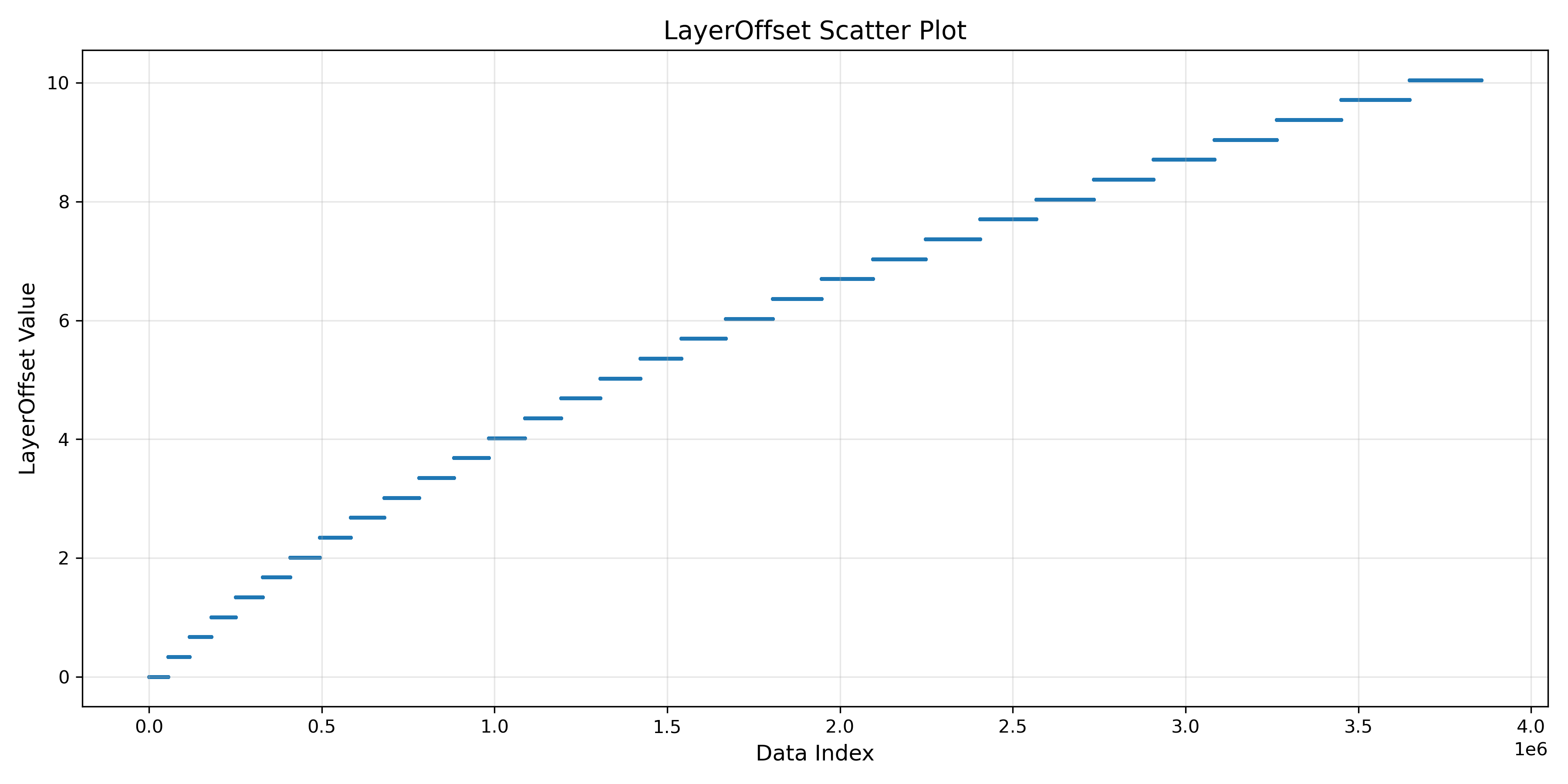

Supplement: Supplementary file 2 — Supporting File 2: advs75661‐sup‐0002‐Python_Stacking_GNS.zip. [file ADVS-9999-e24370-s003.zip › Python_Stacking_GNS(Multi-layer)/layer_offset_scatter.png]

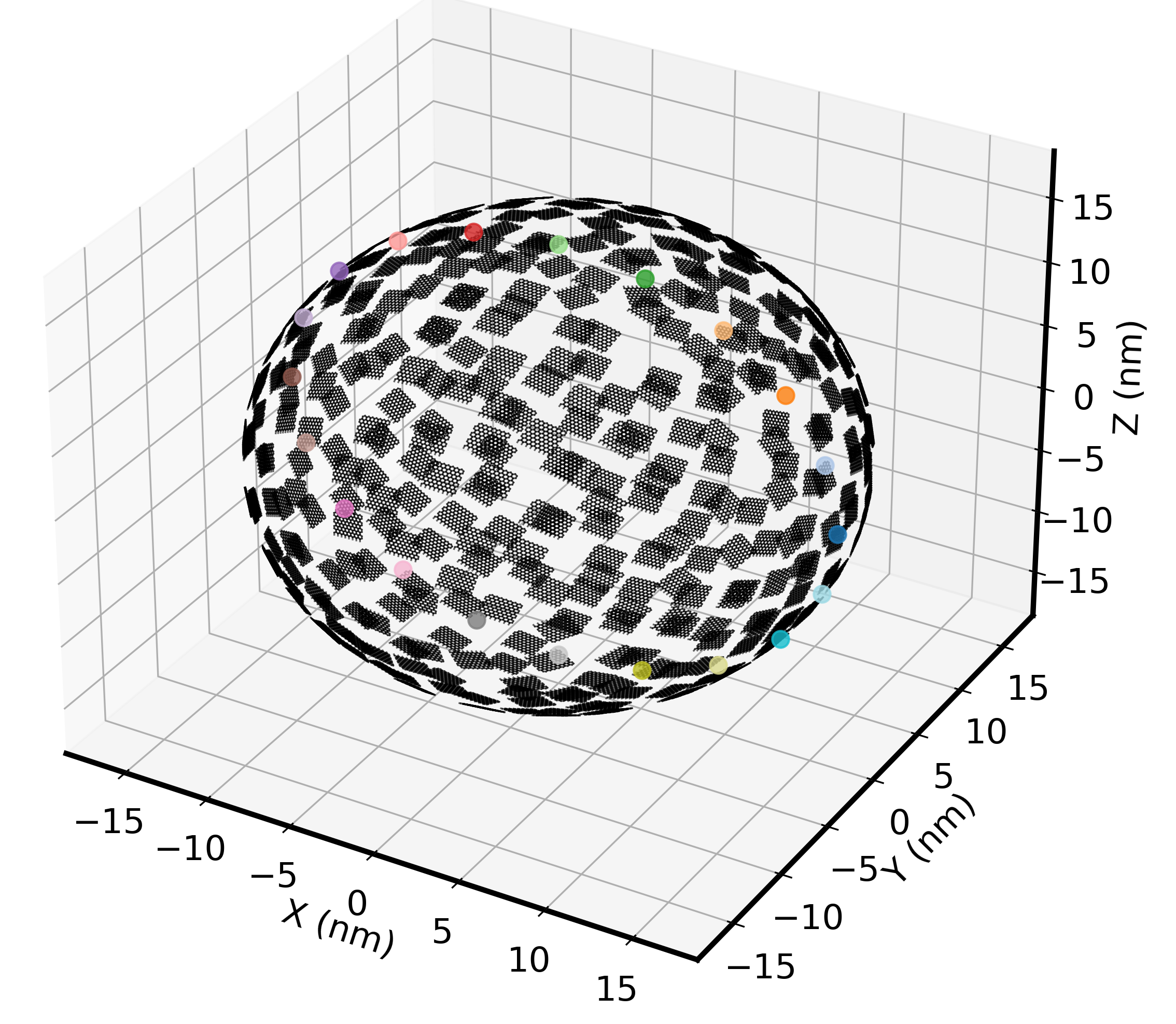

Supplement: Supplementary file 2 — Supporting File 2: advs75661‐sup‐0002‐Python_Stacking_GNS.zip. [file ADVS-9999-e24370-s003.zip › Python_Stacking_GNS(Single-layer)/graphene_plots/graphene_plots/3d_distribution.png]

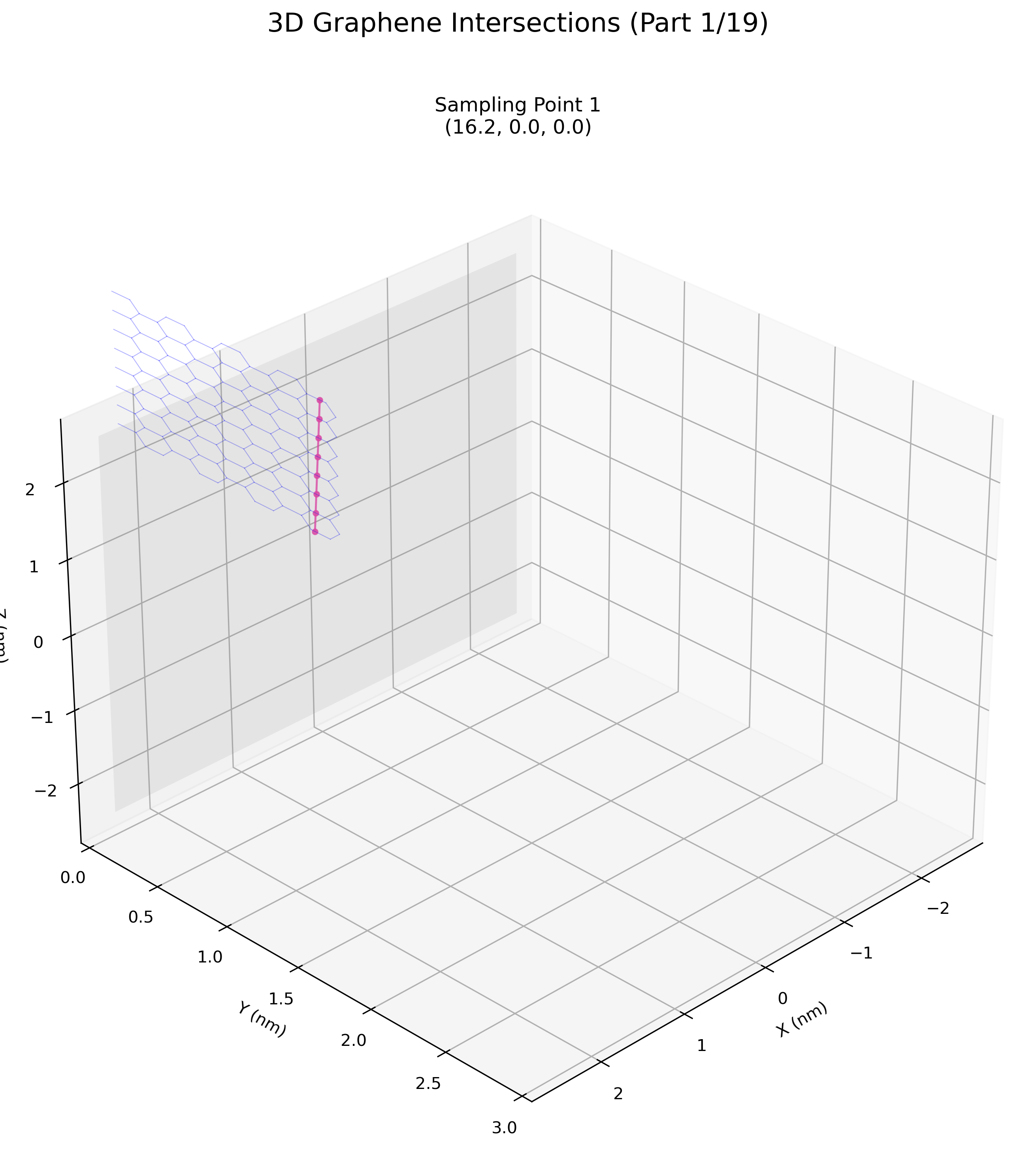

Supplement: Supplementary file 2 — Supporting File 2: advs75661‐sup‐0002‐Python_Stacking_GNS.zip. [file ADVS-9999-e24370-s003.zip › Python_Stacking_GNS(Single-layer)/graphene_plots/graphene_plots/3d_intersection_lines_detailed_1.png]

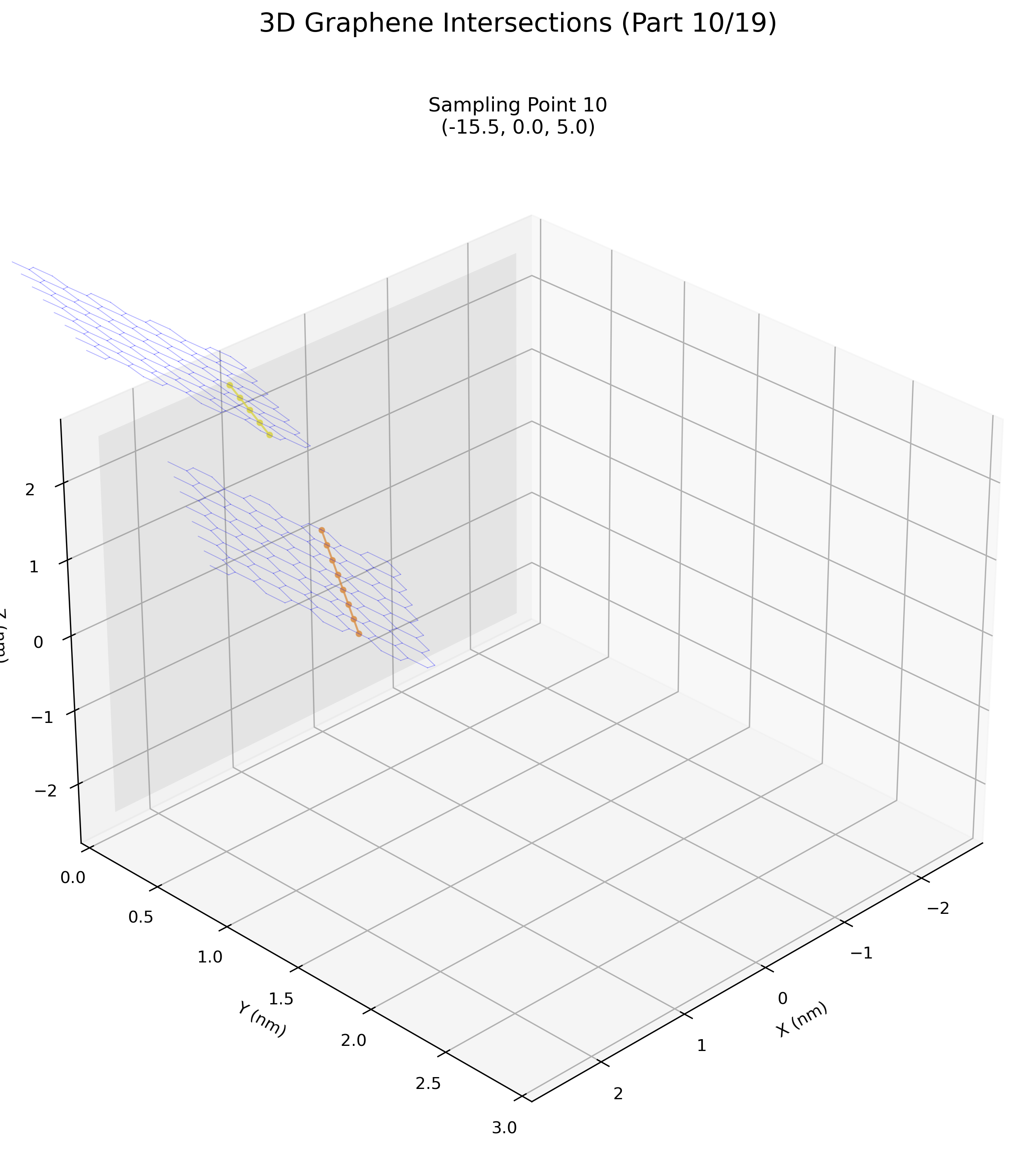

Supplement: Supplementary file 2 — Supporting File 2: advs75661‐sup‐0002‐Python_Stacking_GNS.zip. [file ADVS-9999-e24370-s003.zip › Python_Stacking_GNS(Single-layer)/graphene_plots/graphene_plots/3d_intersection_lines_detailed_10.png]

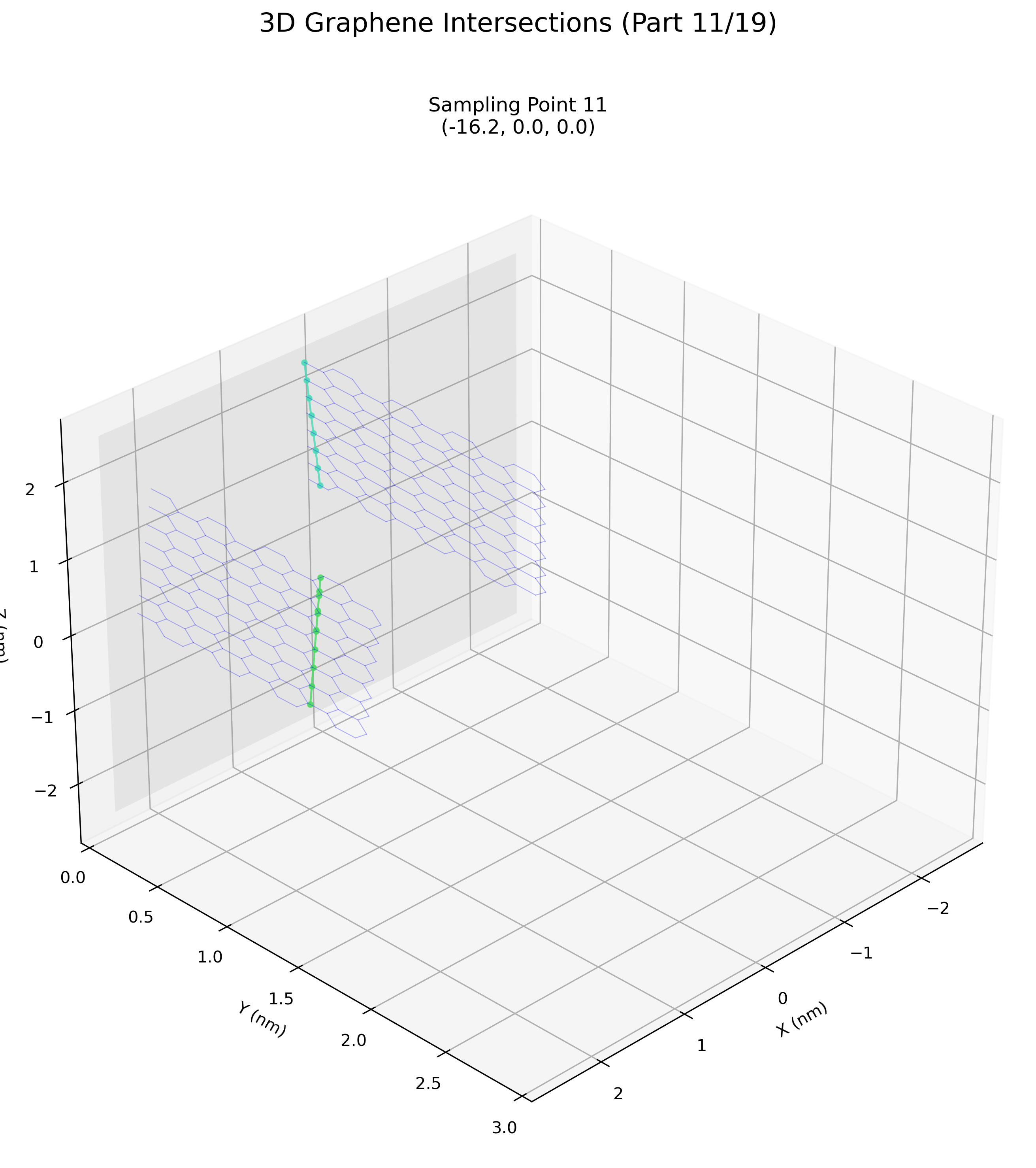

Supplement: Supplementary file 2 — Supporting File 2: advs75661‐sup‐0002‐Python_Stacking_GNS.zip. [file ADVS-9999-e24370-s003.zip › Python_Stacking_GNS(Single-layer)/graphene_plots/graphene_plots/3d_intersection_lines_detailed_11.png]

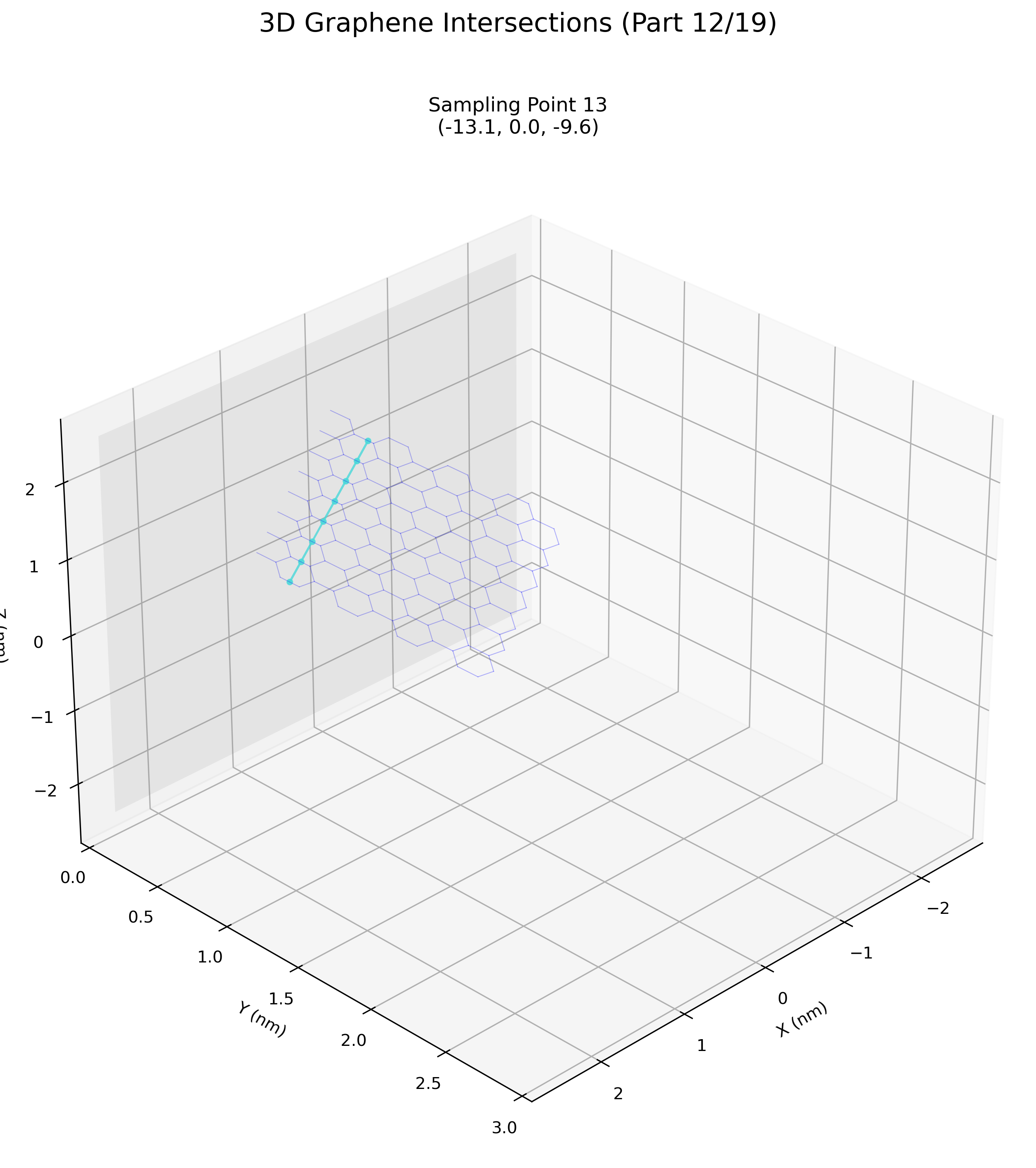

Supplement: Supplementary file 2 — Supporting File 2: advs75661‐sup‐0002‐Python_Stacking_GNS.zip. [file ADVS-9999-e24370-s003.zip › Python_Stacking_GNS(Single-layer)/graphene_plots/graphene_plots/3d_intersection_lines_detailed_12.png]

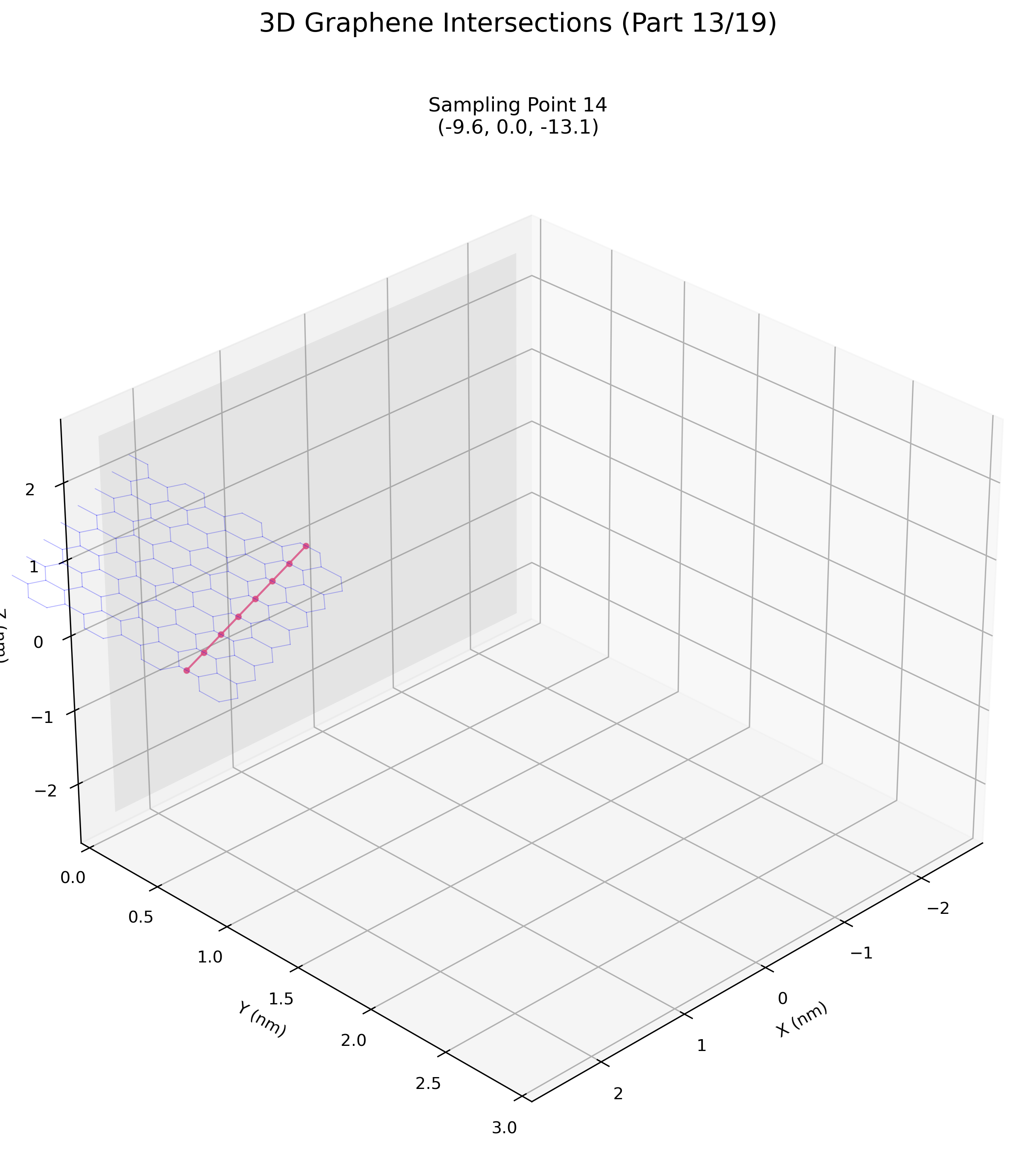

Supplement: Supplementary file 2 — Supporting File 2: advs75661‐sup‐0002‐Python_Stacking_GNS.zip. [file ADVS-9999-e24370-s003.zip › Python_Stacking_GNS(Single-layer)/graphene_plots/graphene_plots/3d_intersection_lines_detailed_13.png]

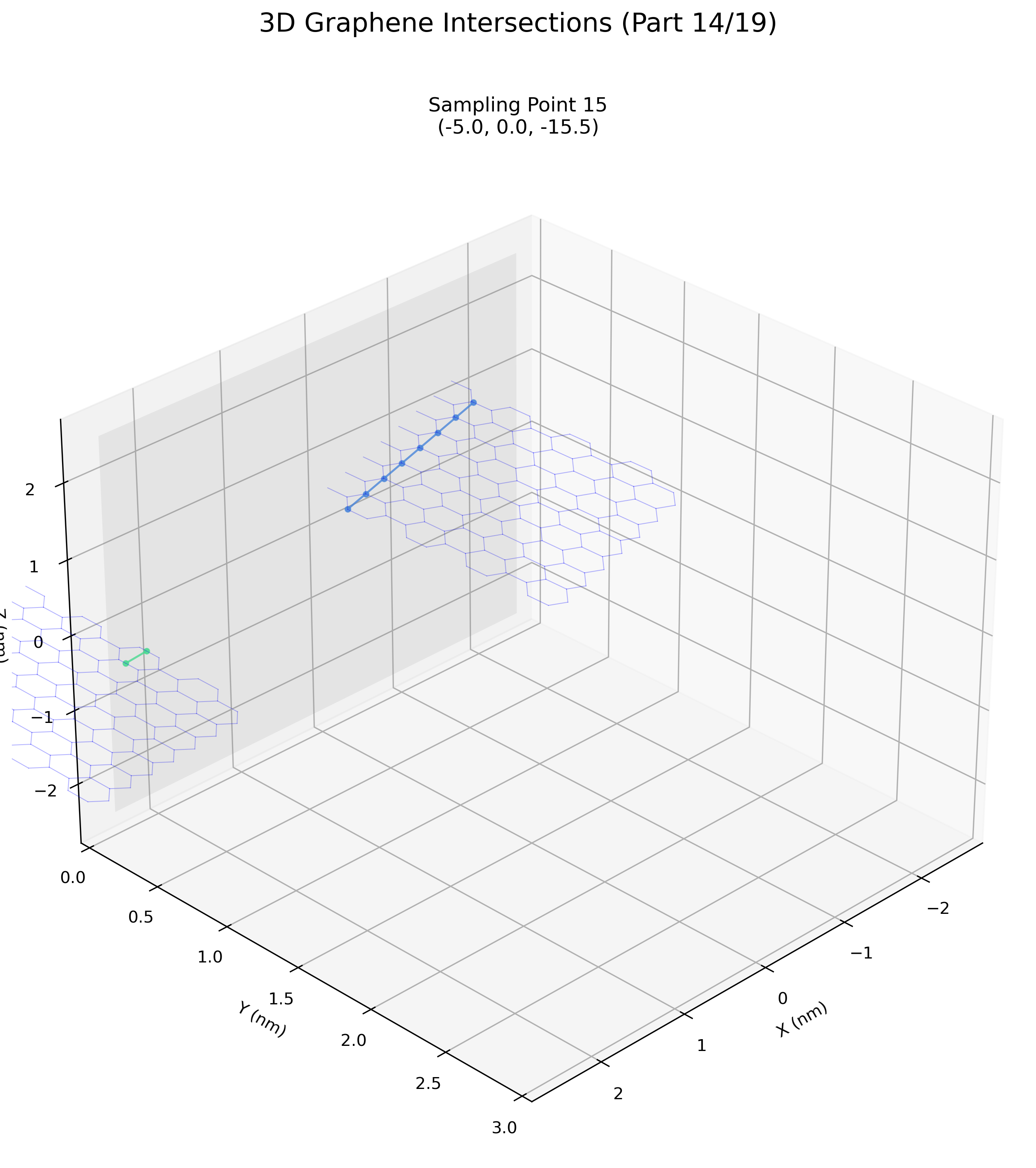

Supplement: Supplementary file 2 — Supporting File 2: advs75661‐sup‐0002‐Python_Stacking_GNS.zip. [file ADVS-9999-e24370-s003.zip › Python_Stacking_GNS(Single-layer)/graphene_plots/graphene_plots/3d_intersection_lines_detailed_14.png]

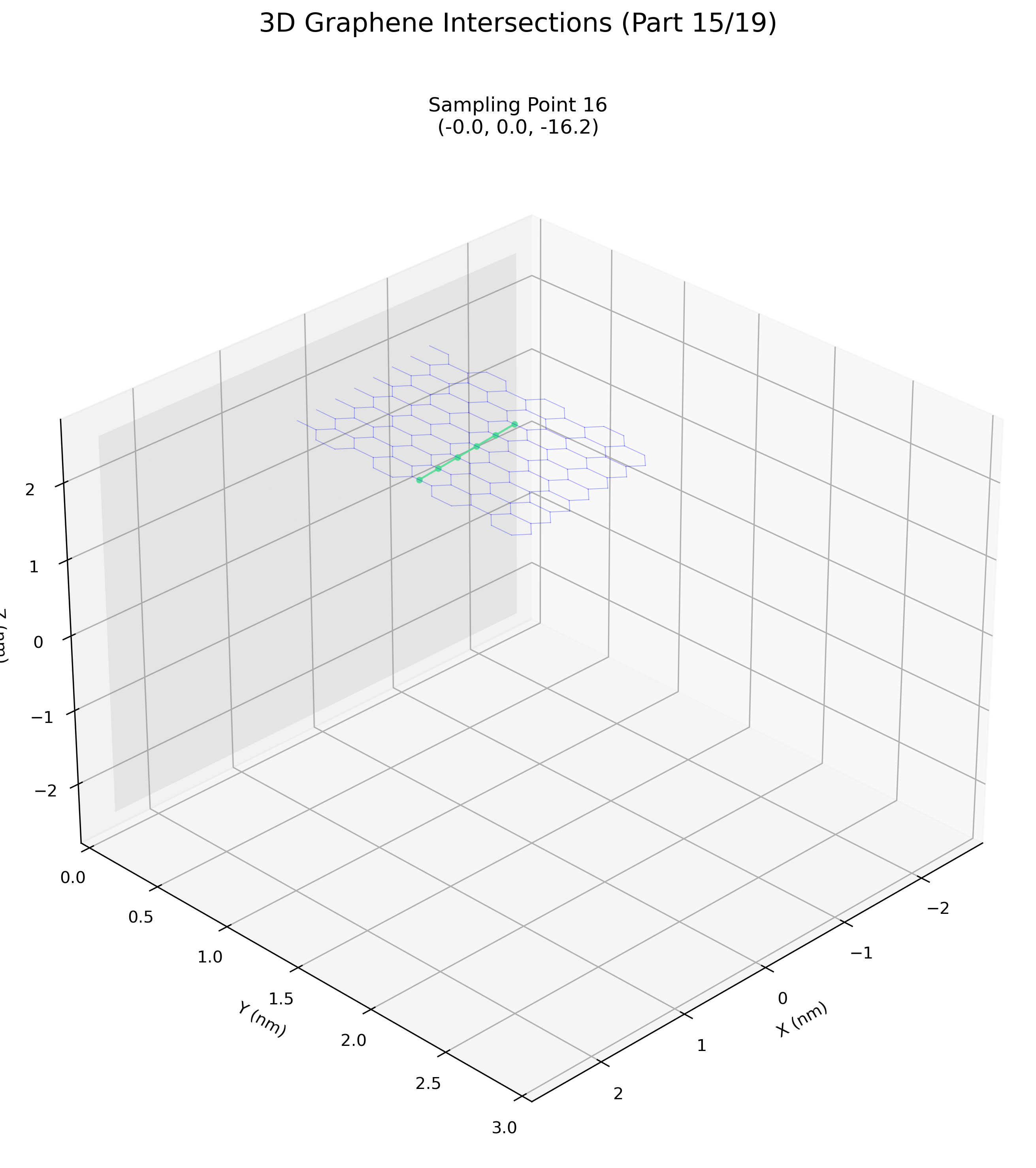

Supplement: Supplementary file 2 — Supporting File 2: advs75661‐sup‐0002‐Python_Stacking_GNS.zip. [file ADVS-9999-e24370-s003.zip › Python_Stacking_GNS(Single-layer)/graphene_plots/graphene_plots/3d_intersection_lines_detailed_15.png]

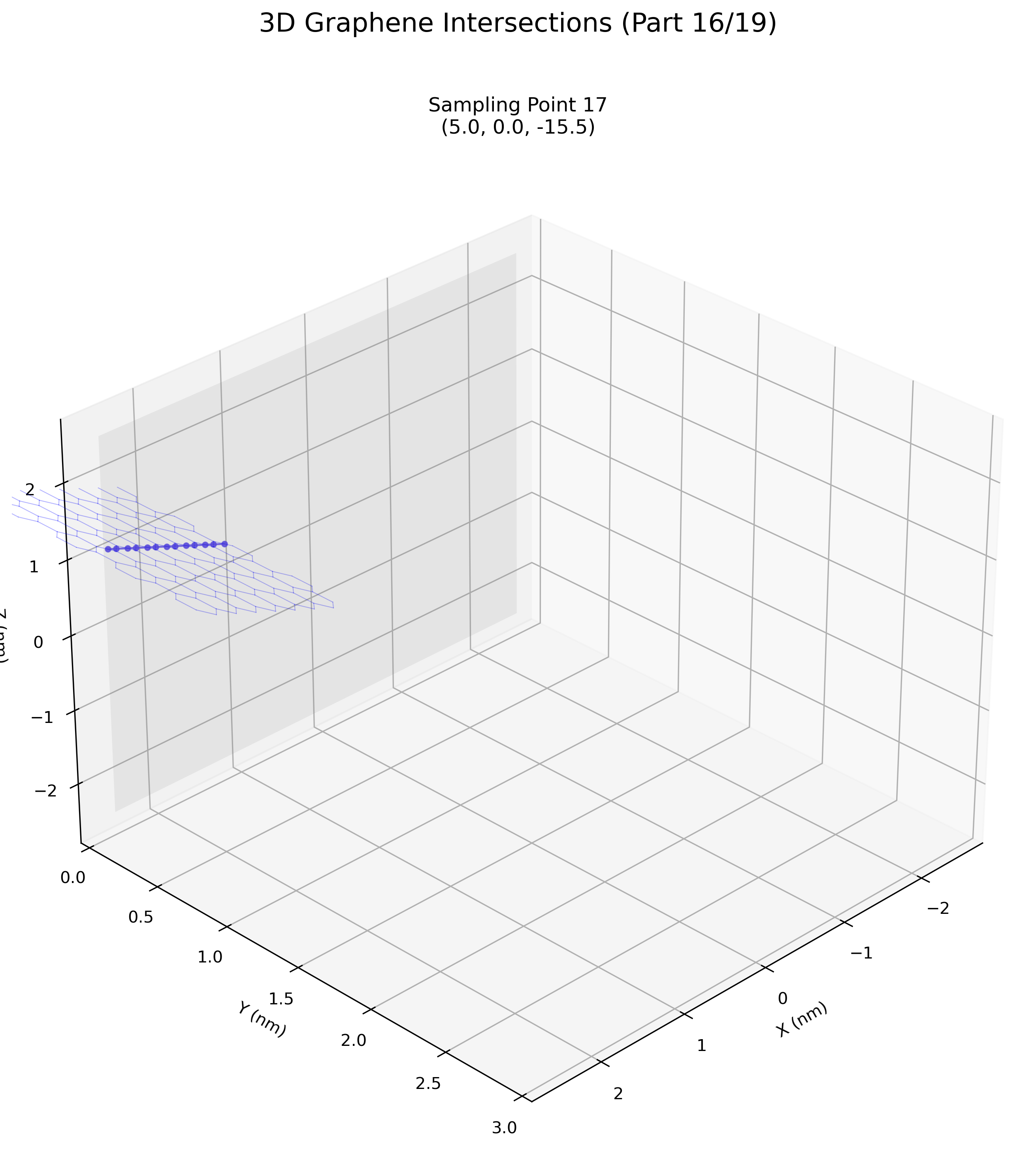

Supplement: Supplementary file 2 — Supporting File 2: advs75661‐sup‐0002‐Python_Stacking_GNS.zip. [file ADVS-9999-e24370-s003.zip › Python_Stacking_GNS(Single-layer)/graphene_plots/graphene_plots/3d_intersection_lines_detailed_16.png]

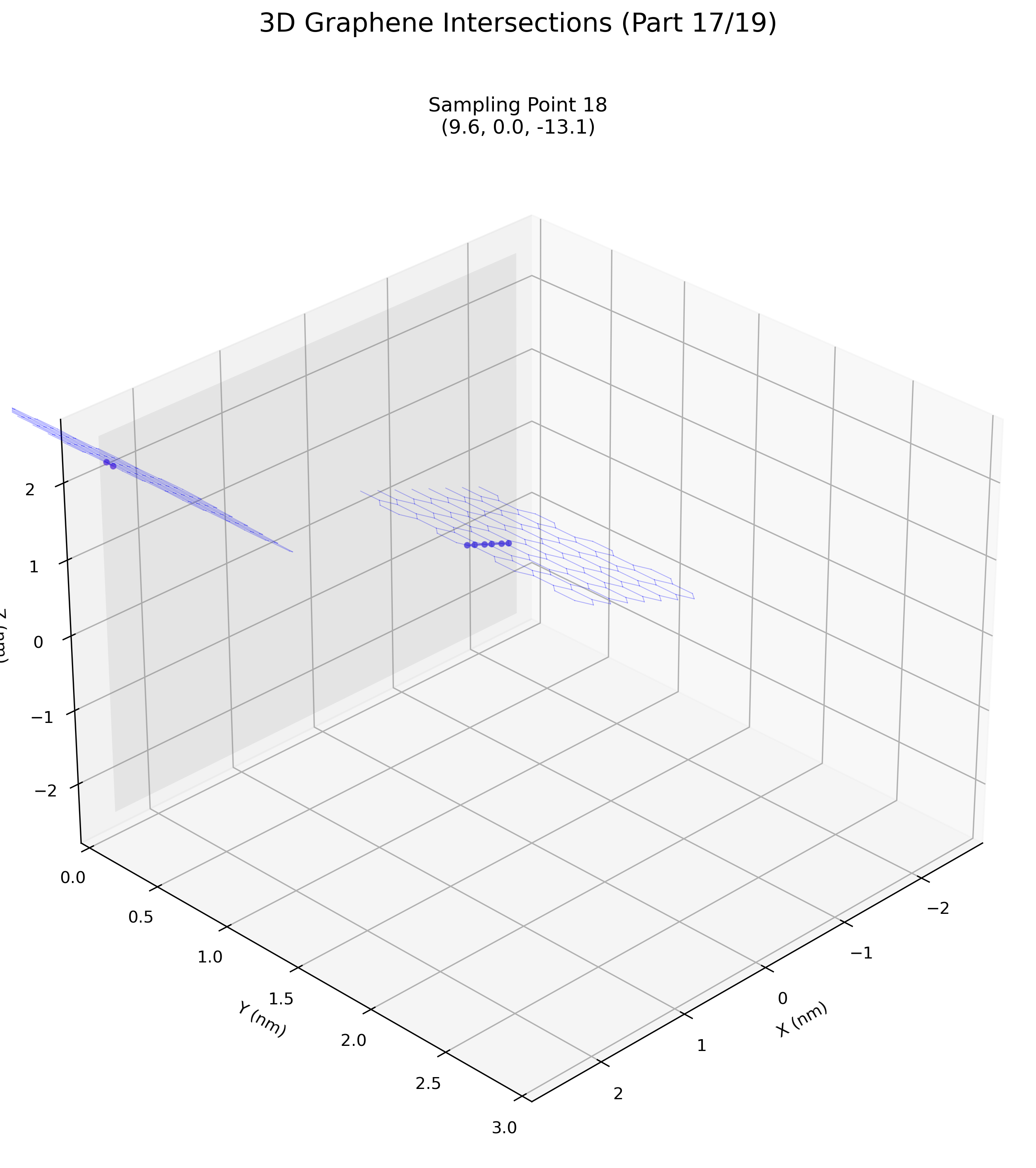

Supplement: Supplementary file 2 — Supporting File 2: advs75661‐sup‐0002‐Python_Stacking_GNS.zip. [file ADVS-9999-e24370-s003.zip › Python_Stacking_GNS(Single-layer)/graphene_plots/graphene_plots/3d_intersection_lines_detailed_17.png]

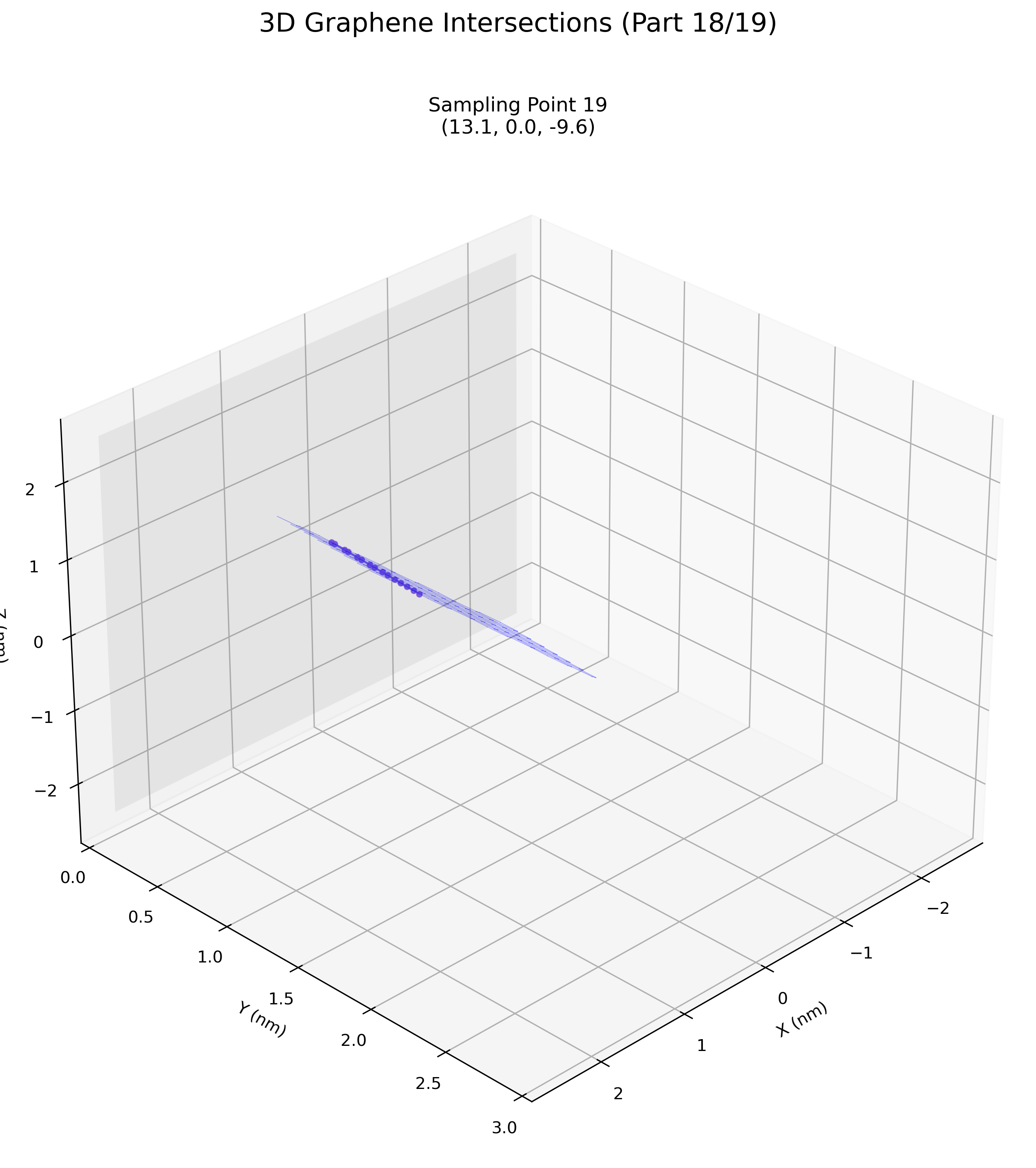

Supplement: Supplementary file 2 — Supporting File 2: advs75661‐sup‐0002‐Python_Stacking_GNS.zip. [file ADVS-9999-e24370-s003.zip › Python_Stacking_GNS(Single-layer)/graphene_plots/graphene_plots/3d_intersection_lines_detailed_18.png]

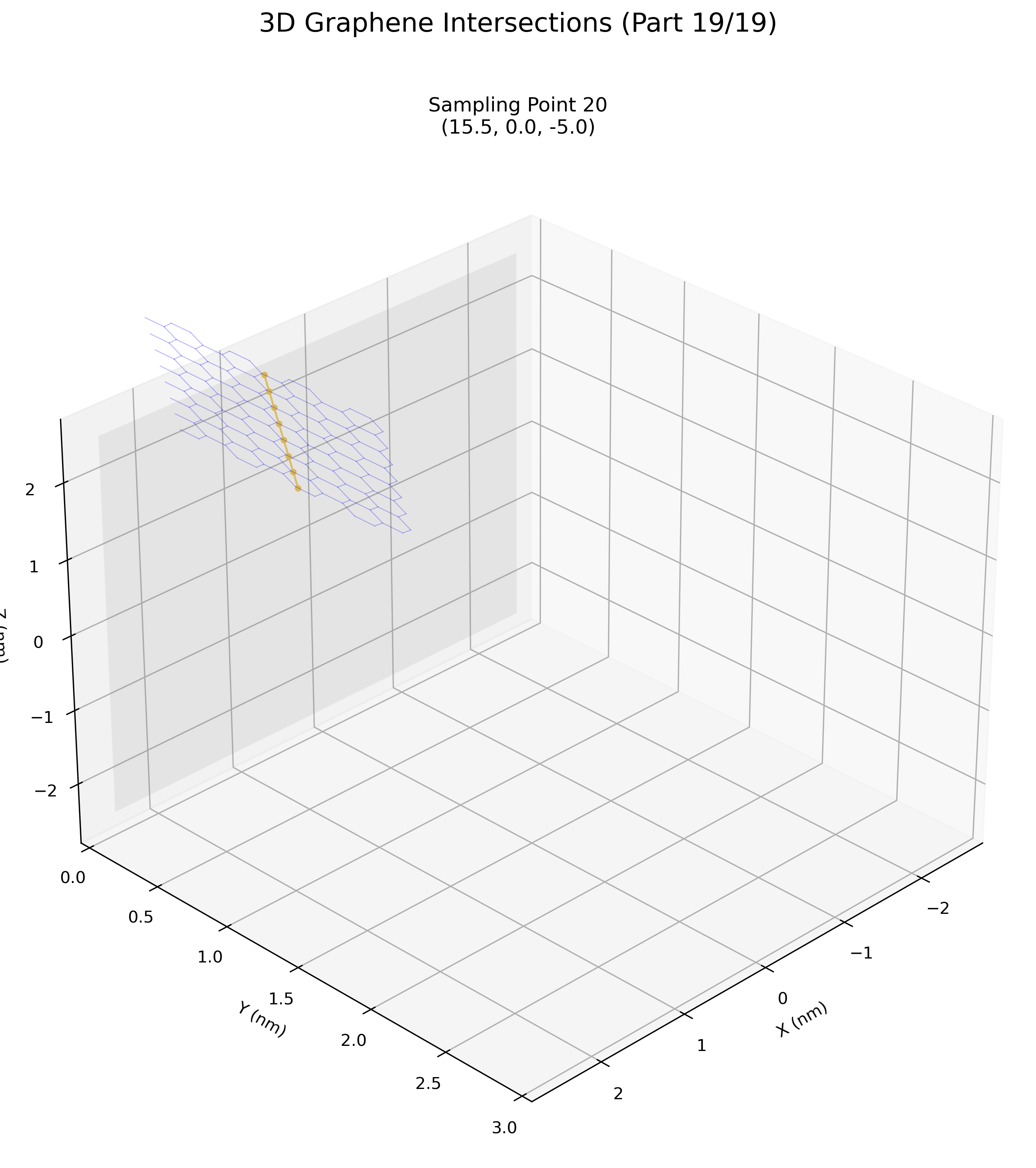

Supplement: Supplementary file 2 — Supporting File 2: advs75661‐sup‐0002‐Python_Stacking_GNS.zip. [file ADVS-9999-e24370-s003.zip › Python_Stacking_GNS(Single-layer)/graphene_plots/graphene_plots/3d_intersection_lines_detailed_19.png]

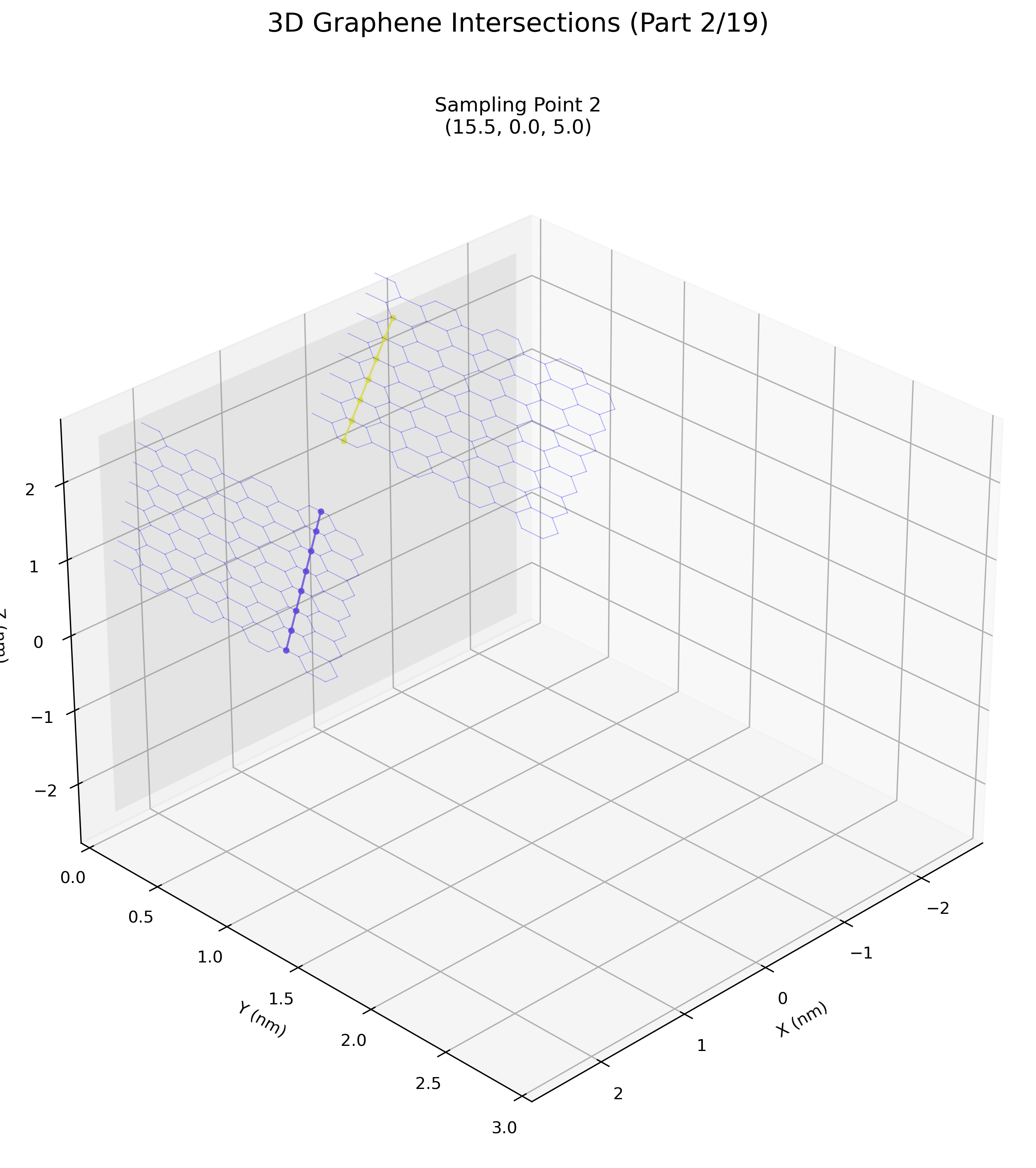

Supplement: Supplementary file 2 — Supporting File 2: advs75661‐sup‐0002‐Python_Stacking_GNS.zip. [file ADVS-9999-e24370-s003.zip › Python_Stacking_GNS(Single-layer)/graphene_plots/graphene_plots/3d_intersection_lines_detailed_2.png]

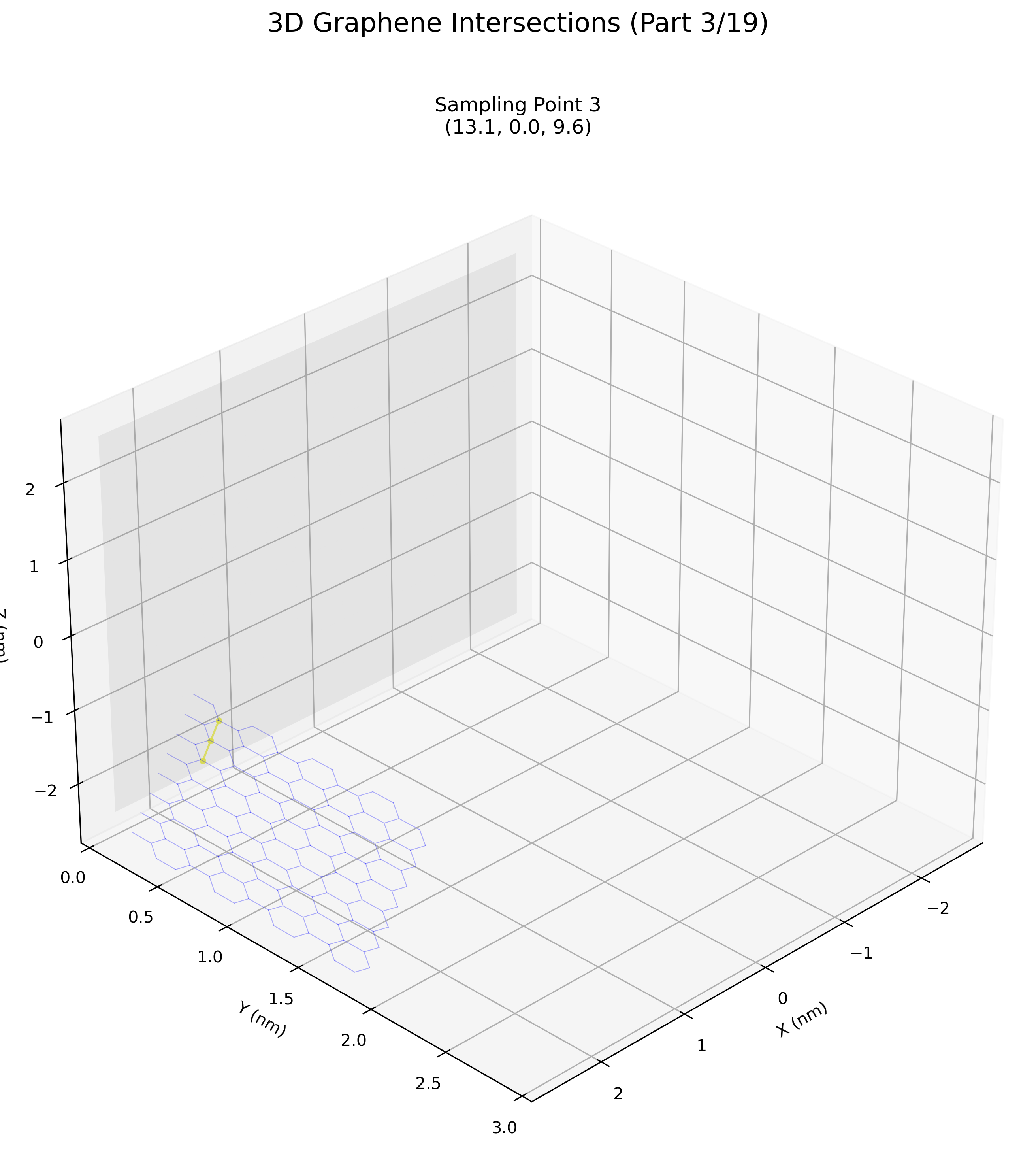

Supplement: Supplementary file 2 — Supporting File 2: advs75661‐sup‐0002‐Python_Stacking_GNS.zip. [file ADVS-9999-e24370-s003.zip › Python_Stacking_GNS(Single-layer)/graphene_plots/graphene_plots/3d_intersection_lines_detailed_3.png]

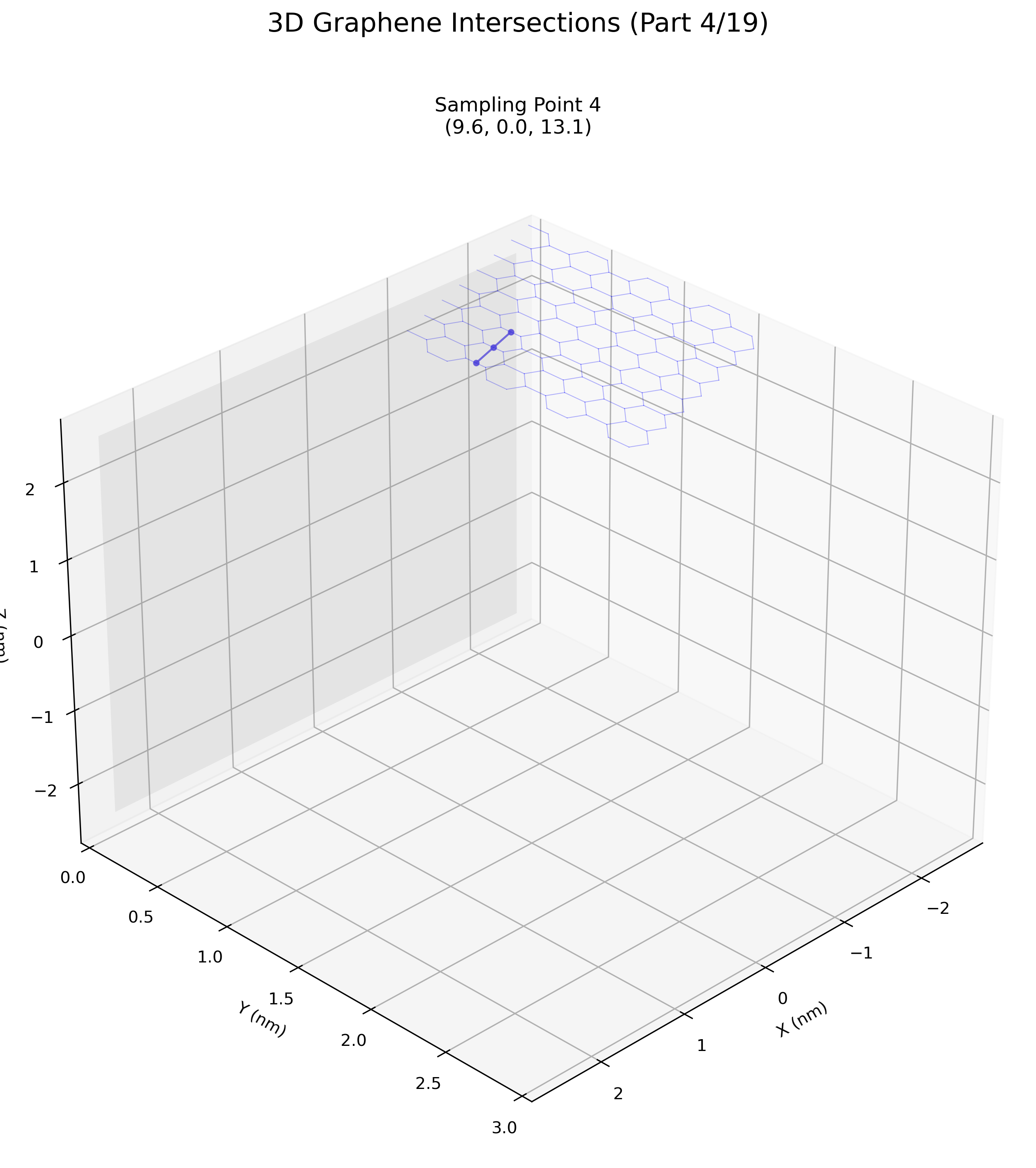

Supplement: Supplementary file 2 — Supporting File 2: advs75661‐sup‐0002‐Python_Stacking_GNS.zip. [file ADVS-9999-e24370-s003.zip › Python_Stacking_GNS(Single-layer)/graphene_plots/graphene_plots/3d_intersection_lines_detailed_4.png]

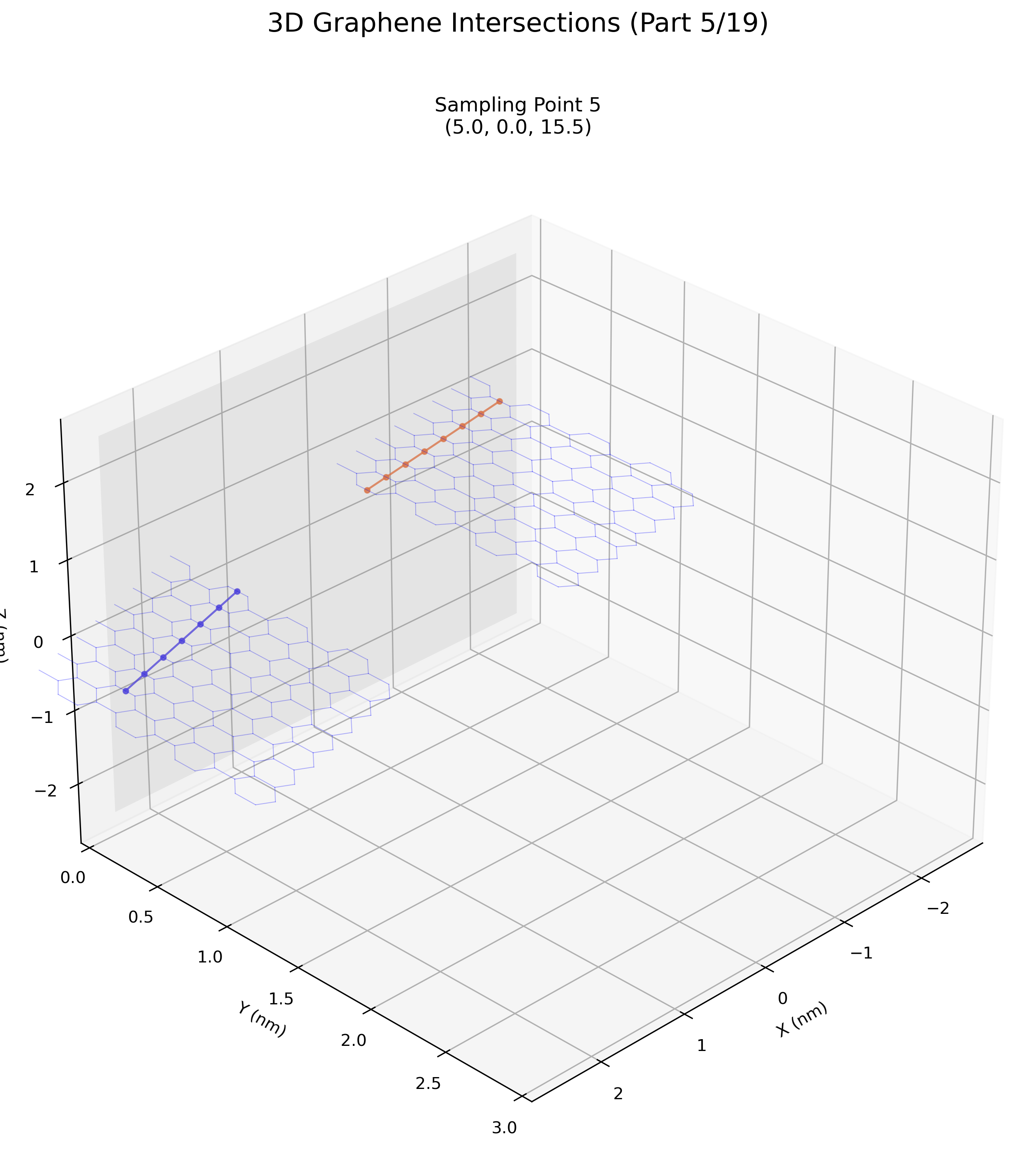

Supplement: Supplementary file 2 — Supporting File 2: advs75661‐sup‐0002‐Python_Stacking_GNS.zip. [file ADVS-9999-e24370-s003.zip › Python_Stacking_GNS(Single-layer)/graphene_plots/graphene_plots/3d_intersection_lines_detailed_5.png]

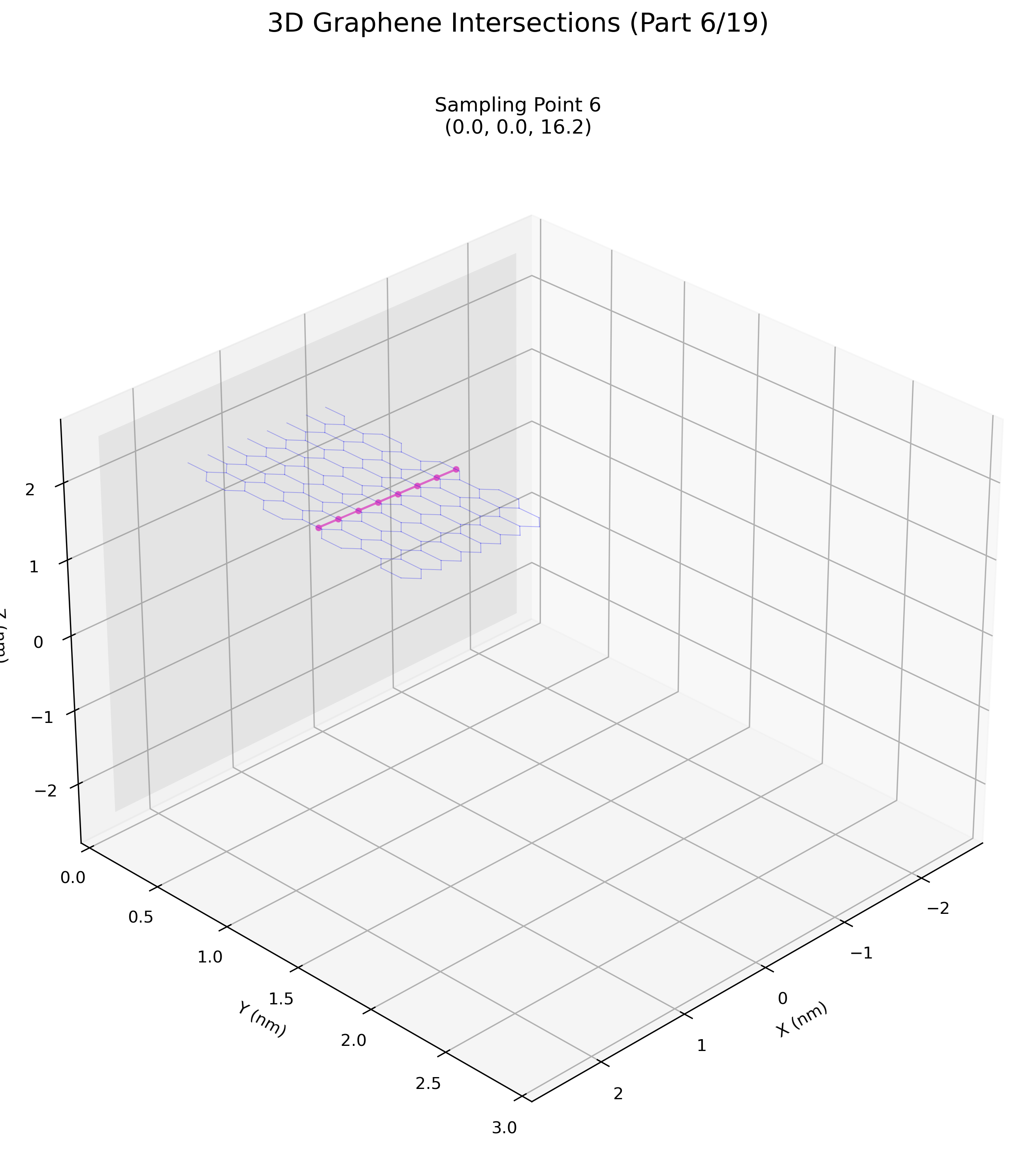

Supplement: Supplementary file 2 — Supporting File 2: advs75661‐sup‐0002‐Python_Stacking_GNS.zip. [file ADVS-9999-e24370-s003.zip › Python_Stacking_GNS(Single-layer)/graphene_plots/graphene_plots/3d_intersection_lines_detailed_6.png]

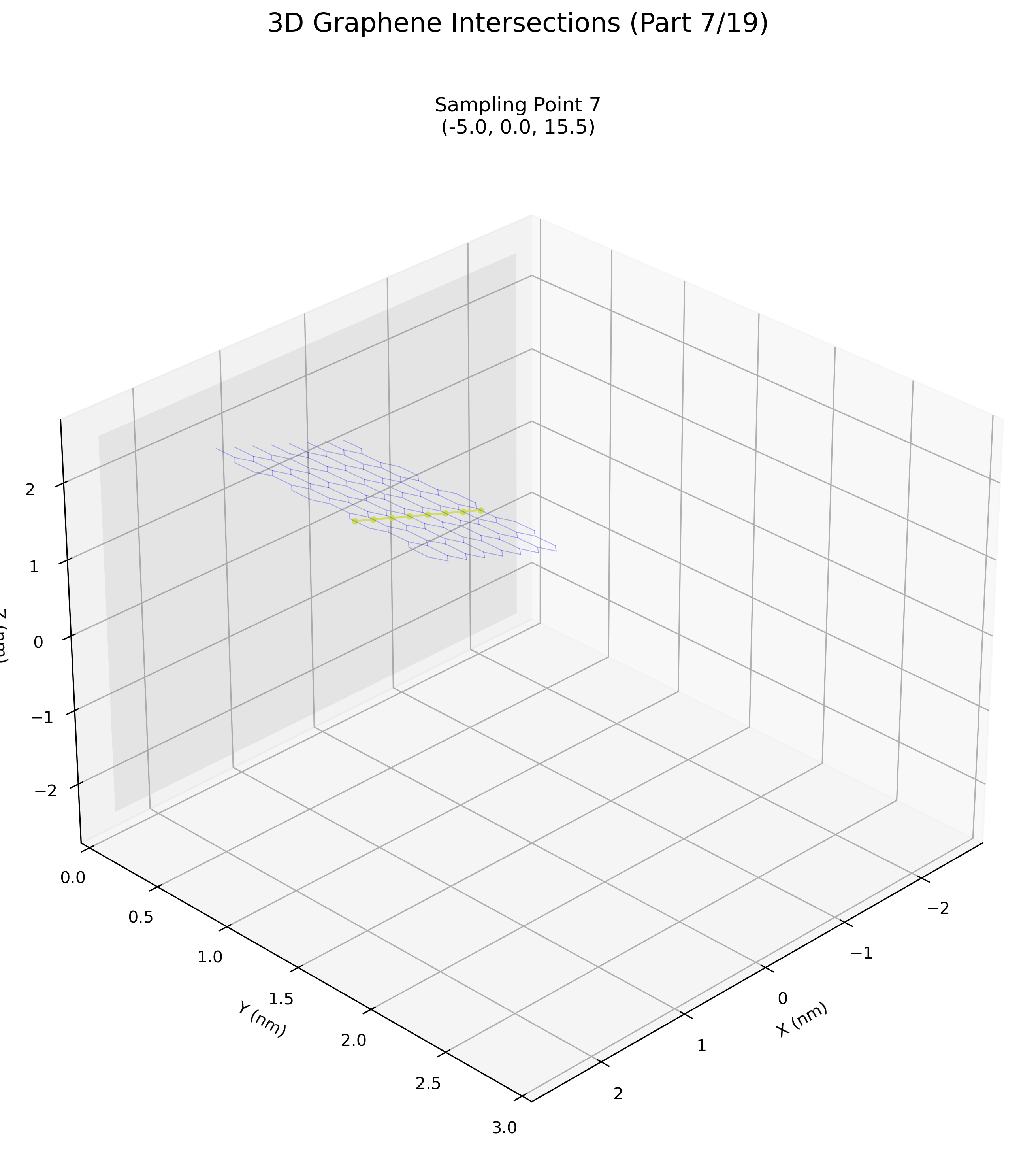

Supplement: Supplementary file 2 — Supporting File 2: advs75661‐sup‐0002‐Python_Stacking_GNS.zip. [file ADVS-9999-e24370-s003.zip › Python_Stacking_GNS(Single-layer)/graphene_plots/graphene_plots/3d_intersection_lines_detailed_7.png]

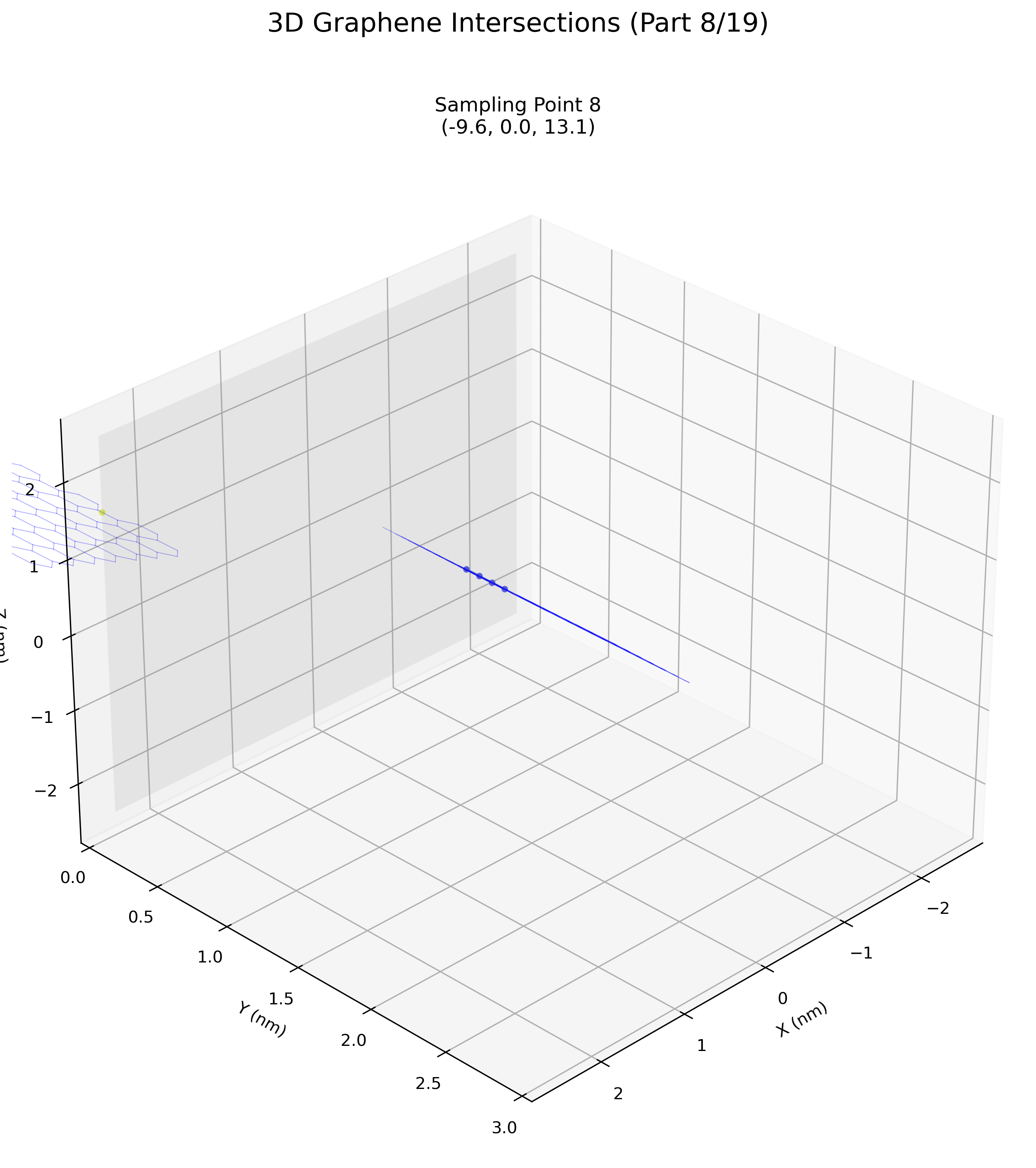

Supplement: Supplementary file 2 — Supporting File 2: advs75661‐sup‐0002‐Python_Stacking_GNS.zip. [file ADVS-9999-e24370-s003.zip › Python_Stacking_GNS(Single-layer)/graphene_plots/graphene_plots/3d_intersection_lines_detailed_8.png]

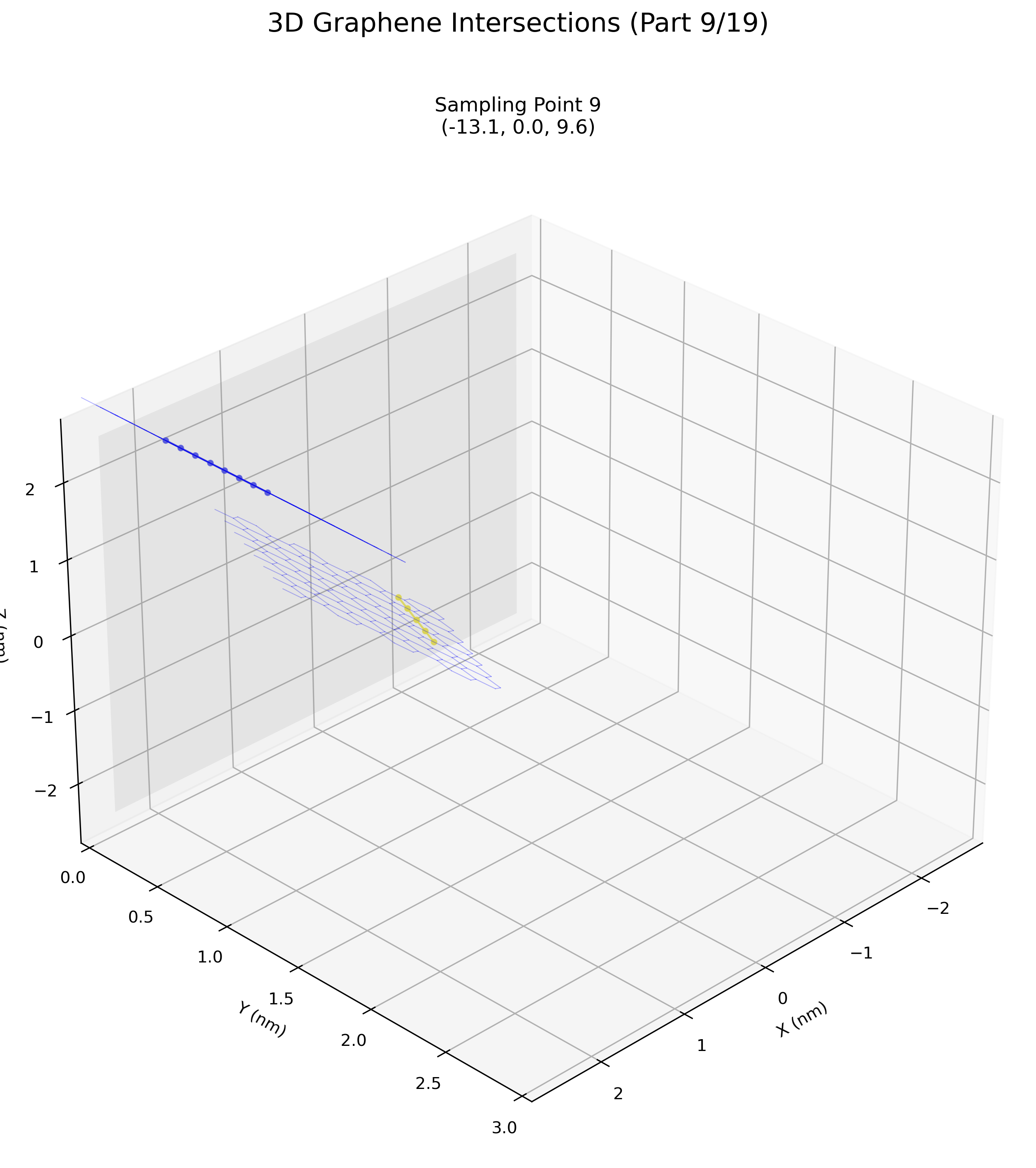

Supplement: Supplementary file 2 — Supporting File 2: advs75661‐sup‐0002‐Python_Stacking_GNS.zip. [file ADVS-9999-e24370-s003.zip › Python_Stacking_GNS(Single-layer)/graphene_plots/graphene_plots/3d_intersection_lines_detailed_9.png]

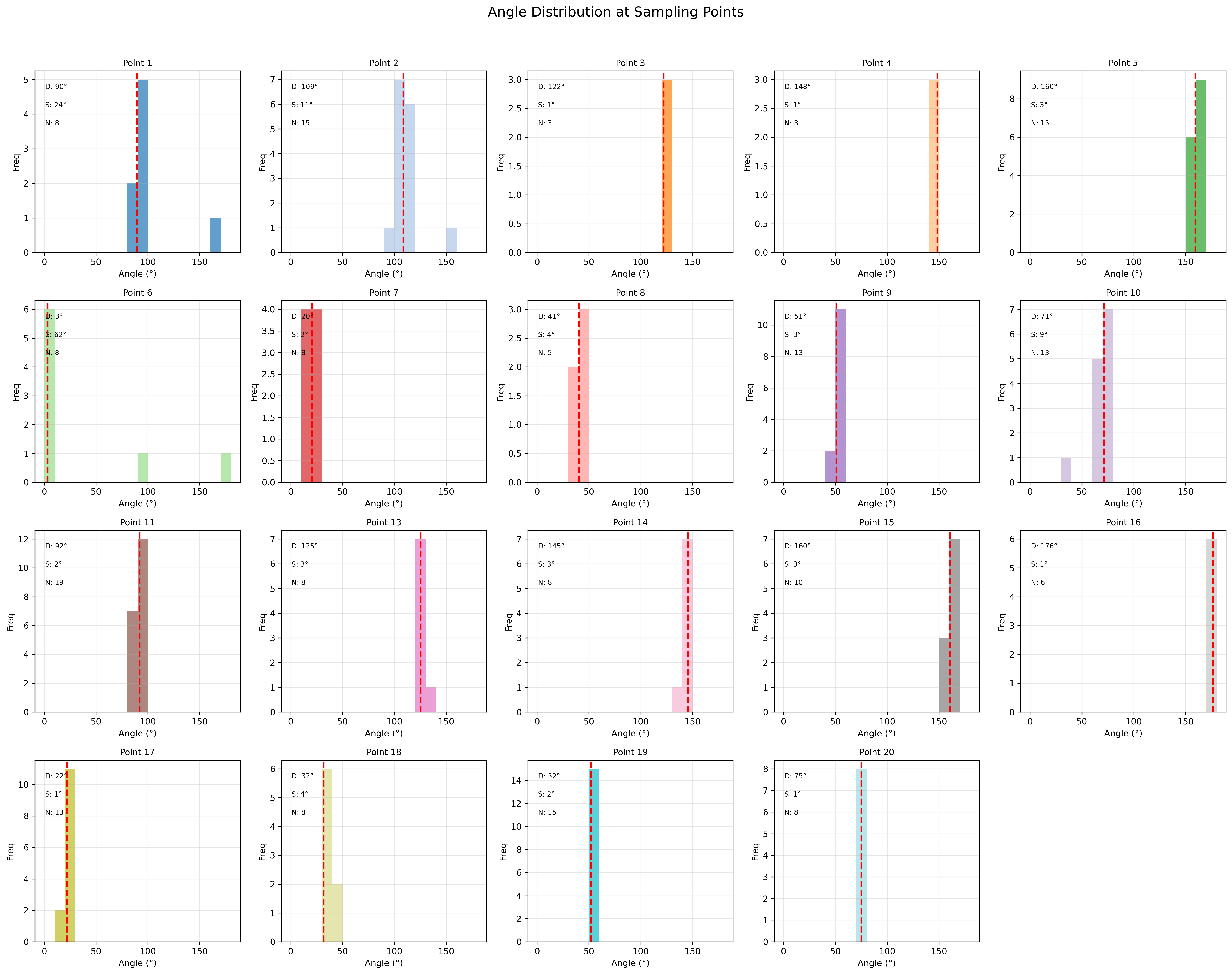

Supplement: Supplementary file 2 — Supporting File 2: advs75661‐sup‐0002‐Python_Stacking_GNS.zip. [file ADVS-9999-e24370-s003.zip › Python_Stacking_GNS(Single-layer)/graphene_plots/graphene_plots/angle_distribution.png]

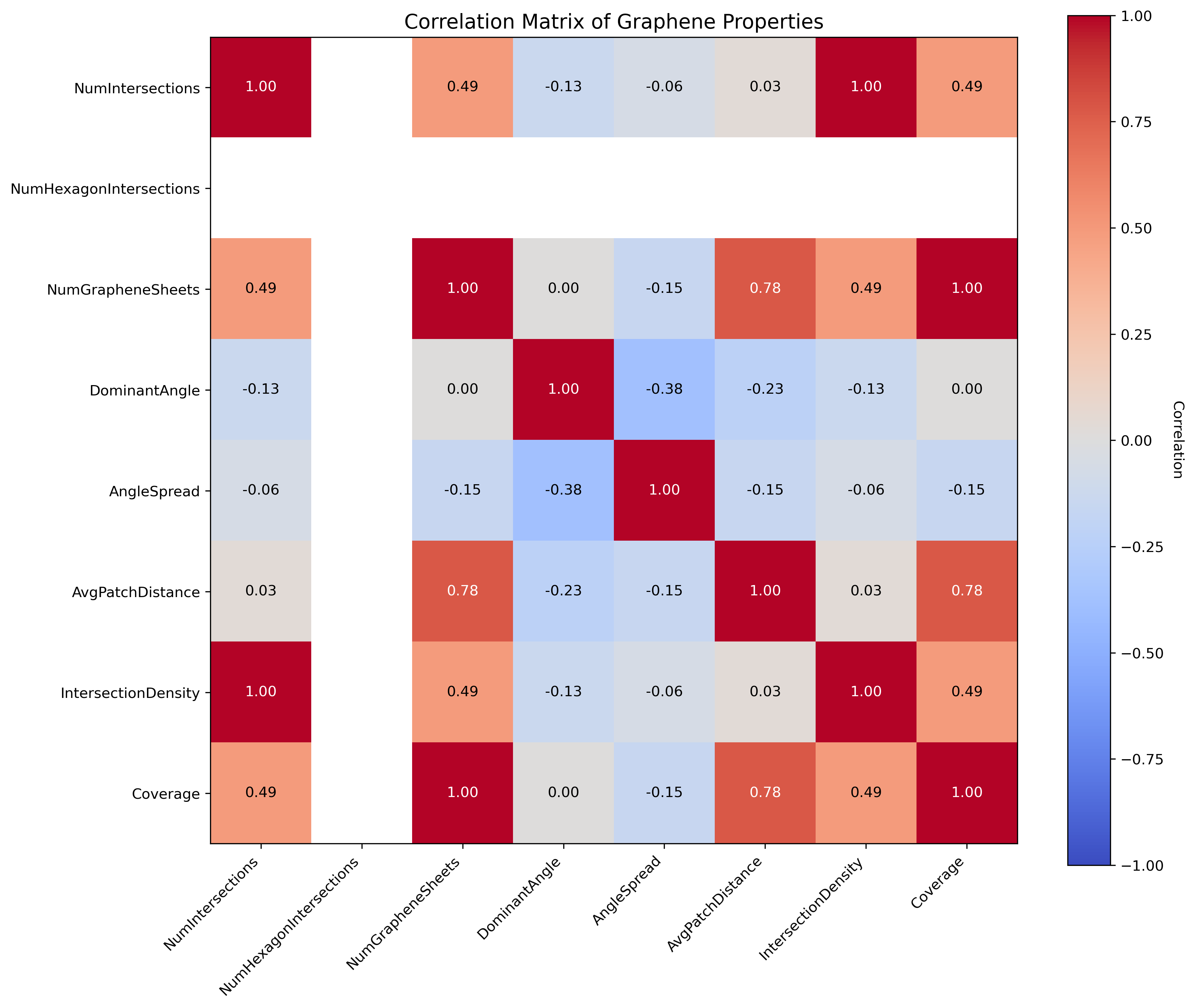

Supplement: Supplementary file 2 — Supporting File 2: advs75661‐sup‐0002‐Python_Stacking_GNS.zip. [file ADVS-9999-e24370-s003.zip › Python_Stacking_GNS(Single-layer)/graphene_plots/graphene_plots/correlation_matrix.png]

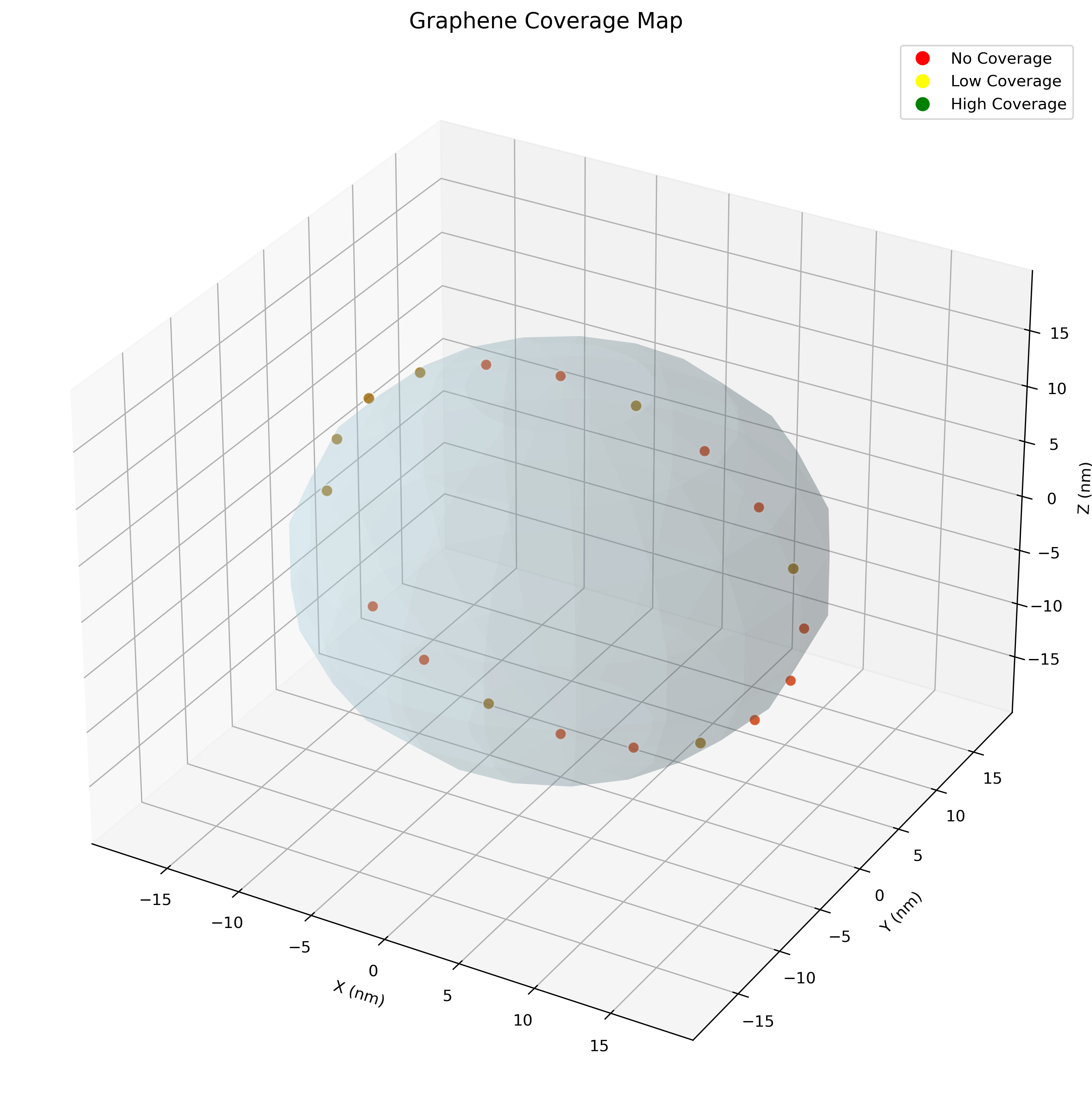

Supplement: Supplementary file 2 — Supporting File 2: advs75661‐sup‐0002‐Python_Stacking_GNS.zip. [file ADVS-9999-e24370-s003.zip › Python_Stacking_GNS(Single-layer)/graphene_plots/graphene_plots/coverage_map.png]

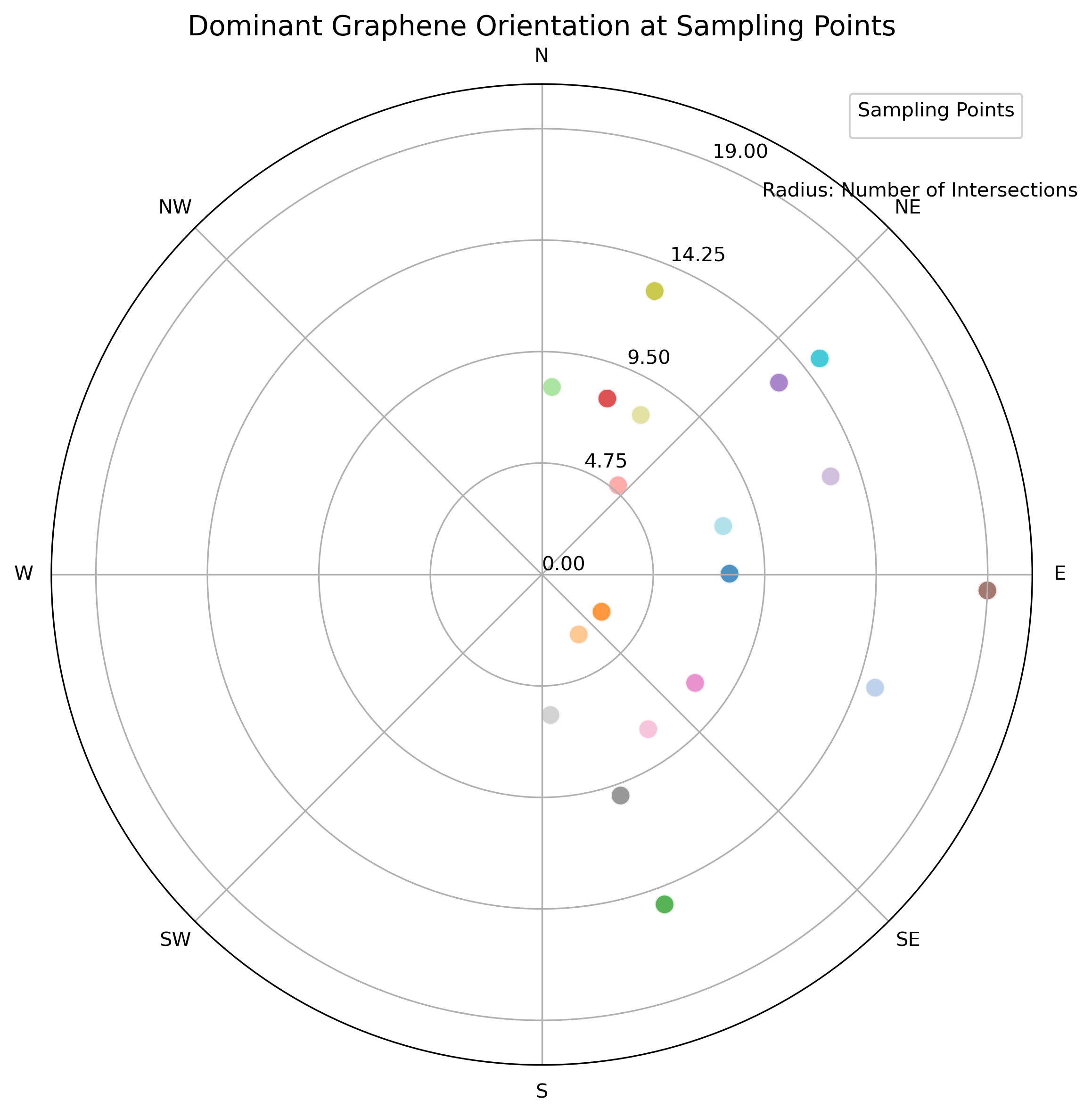

Supplement: Supplementary file 2 — Supporting File 2: advs75661‐sup‐0002‐Python_Stacking_GNS.zip. [file ADVS-9999-e24370-s003.zip › Python_Stacking_GNS(Single-layer)/graphene_plots/graphene_plots/dominant_angles.png]

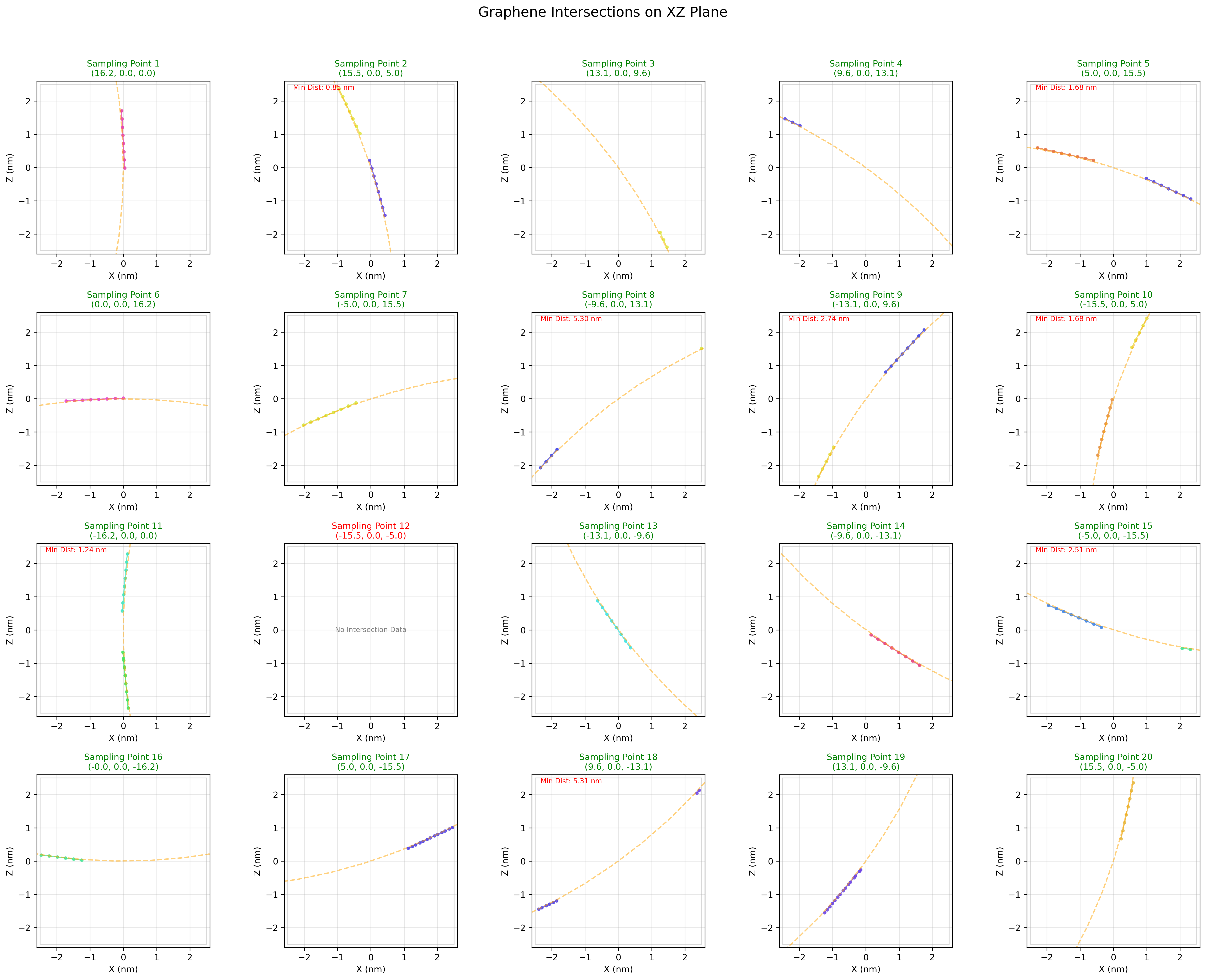

Supplement: Supplementary file 2 — Supporting File 2: advs75661‐sup‐0002‐Python_Stacking_GNS.zip. [file ADVS-9999-e24370-s003.zip › Python_Stacking_GNS(Single-layer)/graphene_plots/graphene_plots/intersection_lines.png]

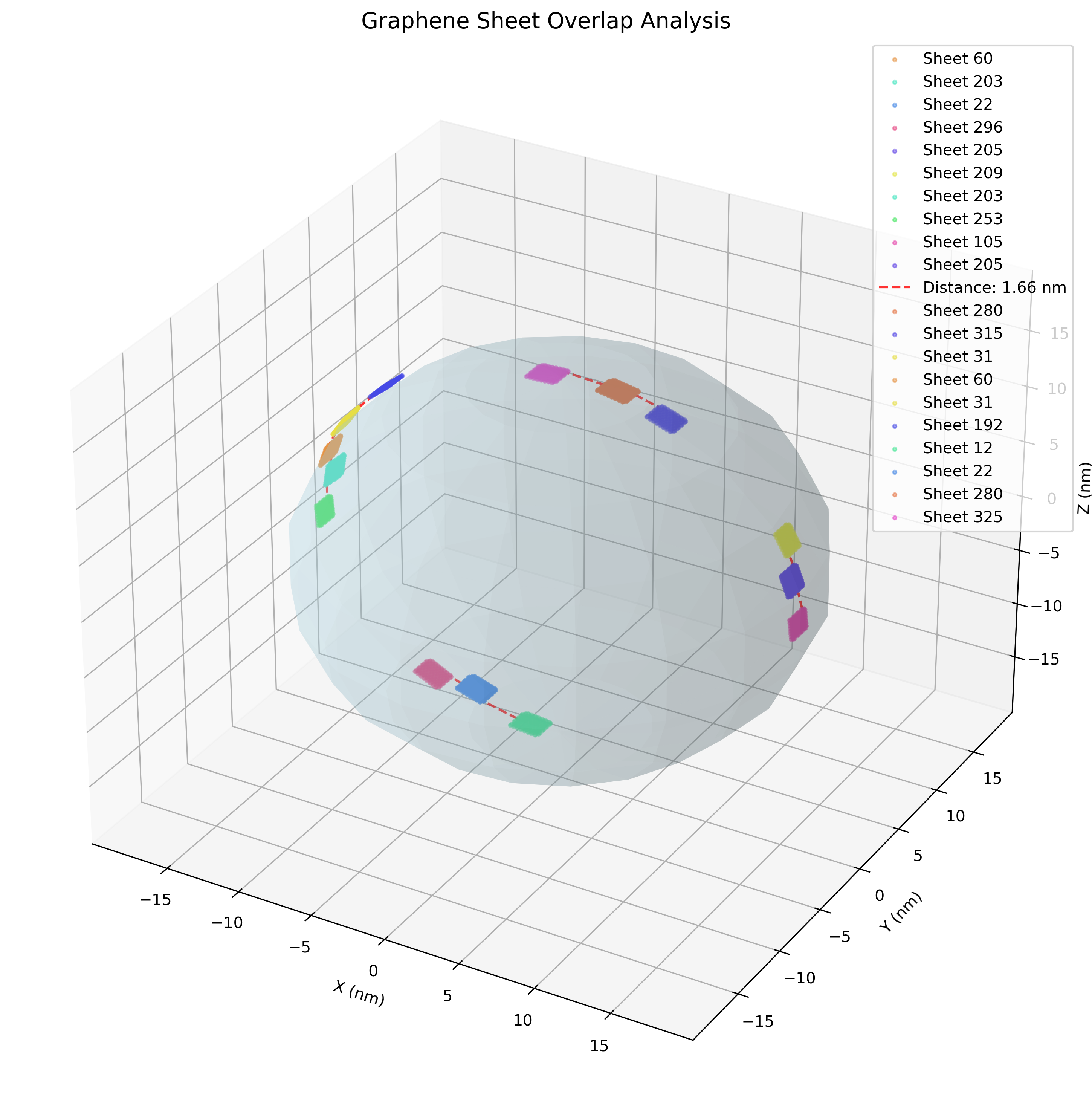

Supplement: Supplementary file 2 — Supporting File 2: advs75661‐sup‐0002‐Python_Stacking_GNS.zip. [file ADVS-9999-e24370-s003.zip › Python_Stacking_GNS(Single-layer)/graphene_plots/graphene_plots/sheet_overlap.png]

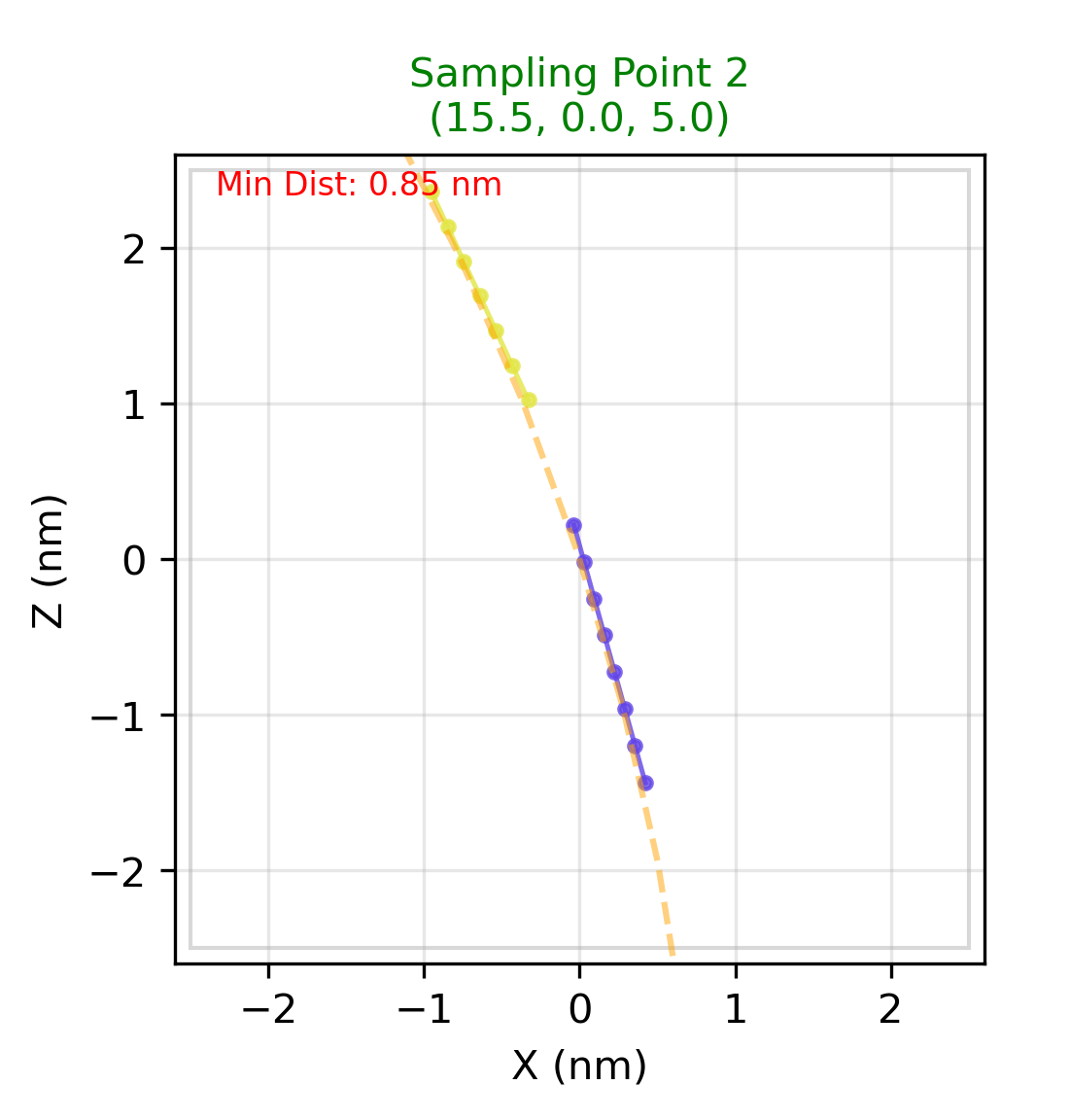

Supplement: Supplementary file 2 — Supporting File 2: advs75661‐sup‐0002‐Python_Stacking_GNS.zip. [file ADVS-9999-e24370-s003.zip › Python_Stacking_GNS(Single-layer)/point2.png]

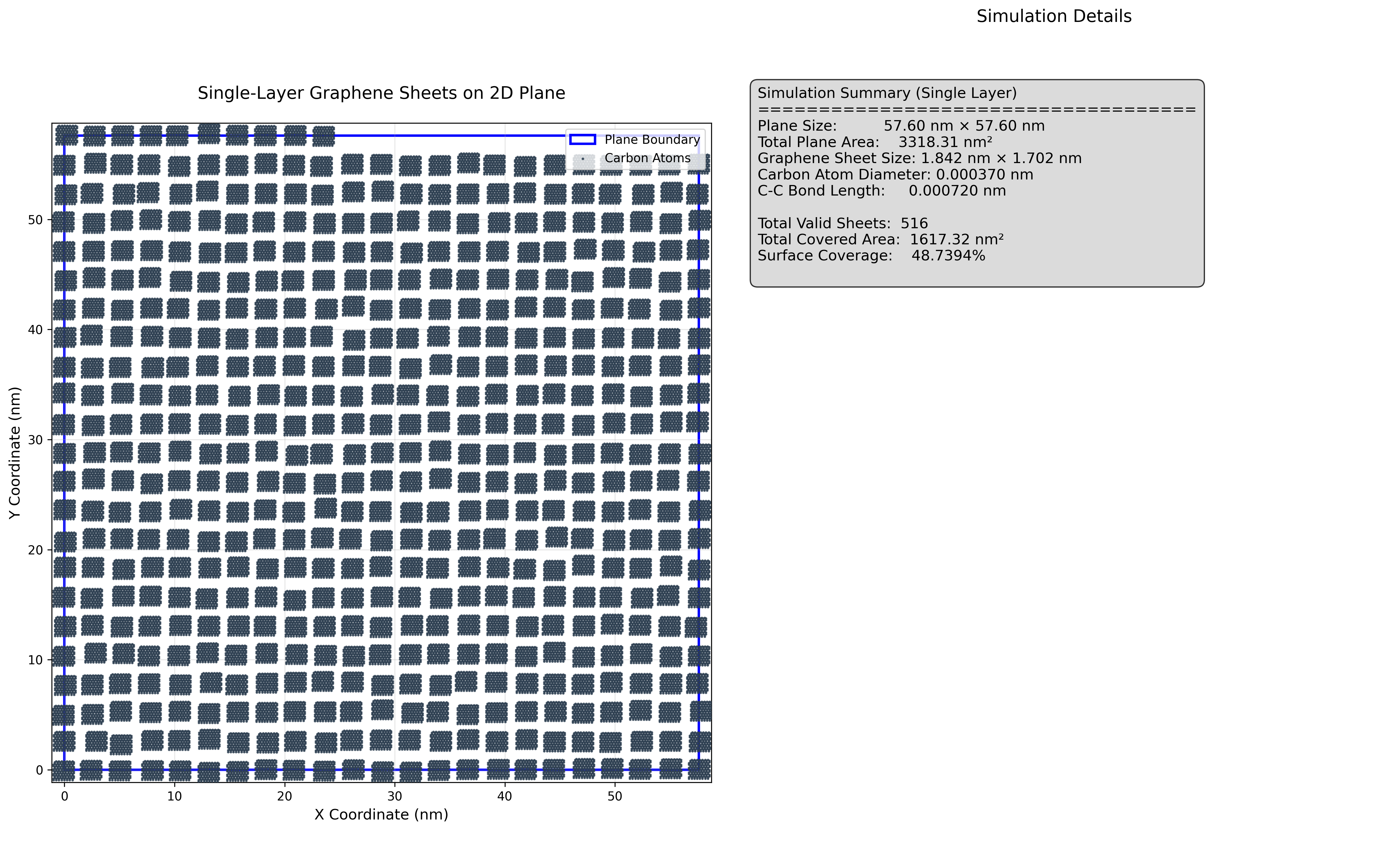

Supplement: Supplementary file 2 — Supporting File 2: advs75661‐sup‐0002‐Python_Stacking_GNS.zip. [file ADVS-9999-e24370-s003.zip › Python_Stacking_GNS(2DPlane)/single_layer_graphene_2d.png]
